# Integrated analyses of miRNA and mRNA profiles in leukocytes and serums in traditional Chinese medicine (TCM)-defined Pi-qi-deficiency syndrome and Pi-wei damp-heat syndrome resulting from chronic atrophic gastritis

**DOI:** 10.1186/s13020-020-00416-9

**Published:** 2021-01-06

**Authors:** Leiming You, Shen Zhang, Ting’an Li, Xiaopu Sang, Kunyu Li, Wei Wang, Xinhui Gao, Jiarui Wu, Guangrui Huang, Ting Wang, Anlong Xu

**Affiliations:** 1 School of Life Sciences, Beijing University of Chinese Medicine, Beijing, 100029 China; 2 School of Chinese Materia Medica, Beijing University of Chinese Medicine, Beijing, 100029 China; 3 State Key Laboratory of Bio-Control, Guangdong Province Key Laboratory of Pharmaceutical Functional Genes, School of Life Sciences, Sun Yat-Sen University, Higher Education Mega Center, Guangzhou, 510006 China

**Keywords:** Chronic atrophic gastritis, Pi-qi-deficiency syndrome, Pi-wei damp-heat syndrome, Leukocyte, Serum, miRNA biomarker

## Abstract

**Background:**

To investigate the microRNA (miRNA)-gene interactions underlying leukocyte functions and characteristics, especially the potential serum biomarkers, implicated in the traditional Chinese medicine (TCM)-defined Pi-qi-deficiency syndrome (PQDS) and Pi-wei damp-heat syndrome (PDHS) resulting from chronic atrophic gastritis (CAG).

**Methods:**

Using RNA/miRNA-sequencing approach, compared with healthy control population, we identified the PDHS- or PQDS*-*specific miRNAs and genes in leukocytes or serums, especially the *Zheng* (syndrome)-specific miRNA-gene interactions, and further decoded their functions and pathways.

**Results:**

Despite being the TCM-defined *Zhengs* resulting from the same disease of CAG, the *Zheng*-specific genes and miRNAs were not same. The PDHS-specific leukocyte genes were mainly involved in defense and immune responses, including NOD-like receptor signaling and several synapses-related pathways. The expression upregulation of PDHS-specific genes enriched in the neutrophil degranulation pathway, indicated the enhanced leukocyte degranulation activation. The PQDS-specific genes in leukocytes were implicated in inflammatory response, extracellular matrix (ECM) organization and collagen catabolism. They could be enriched in MAPK and IL17 signaling and helper T cell differentiation pathways, especially the pathways associated with cell-to-cell adhesion/junction and communication such as cell adhesion molecules, ECM organization and ECM-receptor interaction, probably contributing to the characteristics and functions of leukocytes. Also, the experimentally-supported miRNA-gene interactions, concerned with *COL4A2*, *COL26A1*, *SPP1* and *PROCR*, were implicated in the regulation of pathways related to cell-to-cell adhesion/junction and communication, suggesting the potential roles of the PQDS-specific miRNA-gene interactions for the characteristic and functional changes of leukocytes. Interestingly, the PQDS-specific miRNAs in the serums and the corresponding leukocytes, seemed to have the common roles in contributing to the characteristics and functions of leukocytes. Importantly, the hsa-miR-122-5p could be a potential biomarker, capable of being contained and carried in plasma exosomes and much higher expression in both the leukocytes and corresponding serums in the CAG patients with PQDS rather than PDHS.

**Conclusions:**

These results may provide new insights into the characteristic and functional changes of leukocytes in the two *Zhengs*, PDHS and PQDS, especially the miRNA-mediated gene regulation underlying leukocyte characteristics and functions, with potential leukocyte and serum biomarkers for future application in integrative medicine.

*Trial registration* ClinicalTrials.gov, NCT02915393. Registered on September 17, 2016.

## Background

Traditional Chinese medicine (TCM) is an ancient medical practice system with the longest history in Asia, playing an important role in people’s healthcare and getting more popular in western countries nowdays [5]. The basic theories of TCM contain the rich integrated thoughts, and dialectical thinking is just the essence of TCM. *Zheng* (meaning TCM syndrome), is an integral and essential part of TCM theory [23, 24]. It is a thousand-year-old key diagnostic concept in TCM, defined as a pattern of symptoms and physical signs in a patient at a specific stage during the course of a disease [4, 23, 24]. A TCM-defined *Zheng* of disease is identified through the four-diagnostic methods, with a certain degree of subjectivity and ambiguity from the TCM practitioners [5]. It is the pattern of syndrome that determines the TCM diagnosis of diseases, illuminating what treatment options may be available and should be prescribed for patients.

Interestingly, most patients with chronic atrophic gastritis (CAG) were usually diagnosed with two different TCM syndromes, Pi-qi-deficiency syndrome (PQDS) or Pi-wei damp-heat syndrome (PDHS) [20, 26]. Thus, PQDS and PDHS seem to be two commonly occurring TCM syndrome among CAG patients [2, 10, 13]. However, little is known about the biological basis of TCM-defined PQDS and PDHS, especially the molecular characteristics of the two *Zhengs*. Recent advances in next-generation sequencing (NGS)-based technology, enable the multi-omics analyses of human diseases, notably the discovery of disease-related biomarkers by analyzing tissue or cell-specific transcripts including coding RNAs and non-coding RNAs [9, 15]. MicroRNA (miRNA), a type of endogenous small (20–24 nt) non-coding RNAs, mediates regulation of gene expression at the post-transcriptional level via inhibiting translation of messenger RNA (mRNA) or by inducing the degradation of specific mRNA [1, 3]. Many miRNAs were identified as biomarkers for different human diseases [17, 27, 28], but it is not clear whether miRNAs can serve as the potential prognostic and diagnostic biomarkers for TCM-defined syndromes and diseases, particular the possible miRNA-mRNA interaction networks implicated in maintaining the clinical TCM syndromes.

Thus, in this work, based on the NGS-based miRNA sequencing (miRNA-seq) and RNA sequencing (RNA-seq) approach, using the *control population* (healthy individuals, n = 5), we not only analyzed the differentially expressed miRNAs and genes in the leukocytes of individuals from different patient populations, but also identified the differential circulating miRNAs in serums of them. The patient populations consisted of *case population* 1 (CAG patients with PDHS, n = 5) and *case population* 2 (CAG patients with PQDS, n = 5). The specific miRNAs and genes identified in a certain case population are probably *Zheng* (syndrome)-specific, and we explore their potential roles in contributing to the characteristics and functions of leukocytes, which may be implicated in the TCM diagnosis syndromes (PQDS or PDHS) among CAG patients. In addition, the interaction network analyses of the *Zheng*-specific genes-corresponding proteins were performed to detail the physical and functional protein–protein associations. We especially detailed the *Zheng*-specific miRNAs-mediated regulation of *Zheng*-specific genes in leukocytes based on the experimentally-supported miRNA-target interactions. Also, we evaluated the possibility of location of *Zheng*-specific miRNAs into a plasma exosome, and analyzed their validated targets for the exosome-contained miRNAs to reveal their potential roles when traveling throughout the body.

## Materials and methods

### Ethics approval

The project has been registered at ClinicalTrials.gov (NCT02915393). The protocols have been approved (JDF-IRB-2016031002) by the Institutional Review Board of Dongfang hospital, the affiliated hospital of Beijing University of Chinese Medicine (Beijing, China). All the methods were performed in accordance with the relevant guidelines and regulations. Participants were informed of the purpose, general contents, and data use of the study, and they all signed the informed consent.

### Participants

All the subjects, detailed in Additional file 1: Table S1, were recruited at the hepatobiliary and gastroenterological outpatient’s department of Dongfang hospital. The modern medical diagnosis of CAG was dependent on the CAG pathological diagnosis and grading standards, “*China Chronic Gastritis Consensus*”, which was proposed in Shanghai, 2012 [8]. The TCM syndrome (PQDS or PDHS) diagnosis of CAG patients were based on the *“Guiding Principle for Clinical Research on New Drugs of Traditional Chinese Medicine”* published in 2002 [29]. The experimental design and route, including inclusion and exclusion criteria for the subjects in this study, were detailed in the supplementary methods.

### Leukocytes and serums

Following overnight fasting, blood samples (10 mL) were obtained from each individual by venipuncture into the additive-free blood collection tubes between 8 and 9 _AM_. Blood samples (5 mL) were incubated at 25℃ for 2 h, and the supernatant (serum) was collected and preserved at -80℃. Also, the rest blood samples (5 mL) were used to isolate leukocytes using the lymphocyte separation reagent (Solarbio) according to the manufacture’s instruction.

### RNA sequencing and miRNA sequencing

RNA sequencing and miRNA-sequencing for the leukocyte and serum samples in this study, were performed by OEbiotech company (Shanghai, China). The detailed descriptions for the NGS-based sequencing are provided in the Additional file 1: Methods.

### Identification of the differentially expressed miRNAs and genes

The expression levels of miRNAs and genes, including the novel miRNAs discovered in this study, were standardized and respectively indicated using TPM (transcripts per million, the number of reads per miRNA alignment/the total number of mapped reads × 10^6^) and FPKM (fragments per kilobase of exon model per million mapped reads). The identification of differential miRNAs and genes between groups and the *P*-value calculations were performed using the R package of DESeq [14]. The differential miRNAs and genes among groups were filtered (*P*-value < 0.05 & |log_2_(fold change)| ≥ 1).

### Expression pattern clustering

Hierarchical clustering (HCL)-based expression analyses of differential miRNAs and genes were performed by the well-known Cluster software (v3.0), and the TreeView package (v1.1.6) was used to preview and generate the HCL analysis-based heatmaps [7].

### Target prediction of miRNA

The validated target genes of the differential miRNAs were directly retrieved from the TarBase v7.0, a released database collecting the experimentally supported miRNA-gene interactions, both in vivo and in vitro [22].

### Function and pathway enrichment analysis

The gene ontology (GO) function enrichment analysis of differential genes, were performed using the popular DAVID tool v6.8 [11]. The pathway enrichment analysis of differential genes was conducted using the well-updated KOBAS v3.0 [25]. Particularly, the function and pathway enrichment analyses of the validated target genes of miRNAs, were used by the DIANA tool which is based on the cooperation of the previously-mentioned database (TarBase v7.0) and the mirPath v3.0 (a miRNA pathway analysis web server deciphering miRNA function with experimental support) [16, 21].

### Interaction network analysis

The interaction network of the differential genes-corresponding proteins, were created using the online STRING database v11.0 [19]. Especially, the obtained interaction networks were further modified and integrated using the popular Cytoscape (package v3.7.1) [18].

### Evaluating the possibility of location of a miRNA into a plasma exosome

We especially analyzed weather a miRNA could be encapsuled and carried in a human plasma exosome to function in more recipient cells throughout the body, based on the well-updated *ExoCarta* exosome database that collecting and characterizing the human plasma-derived exosomal RNAs by deep sequencing [12].

## Results

### Differential genes and miRNAs identified in leukocytes and serums

**Fig. 1:**
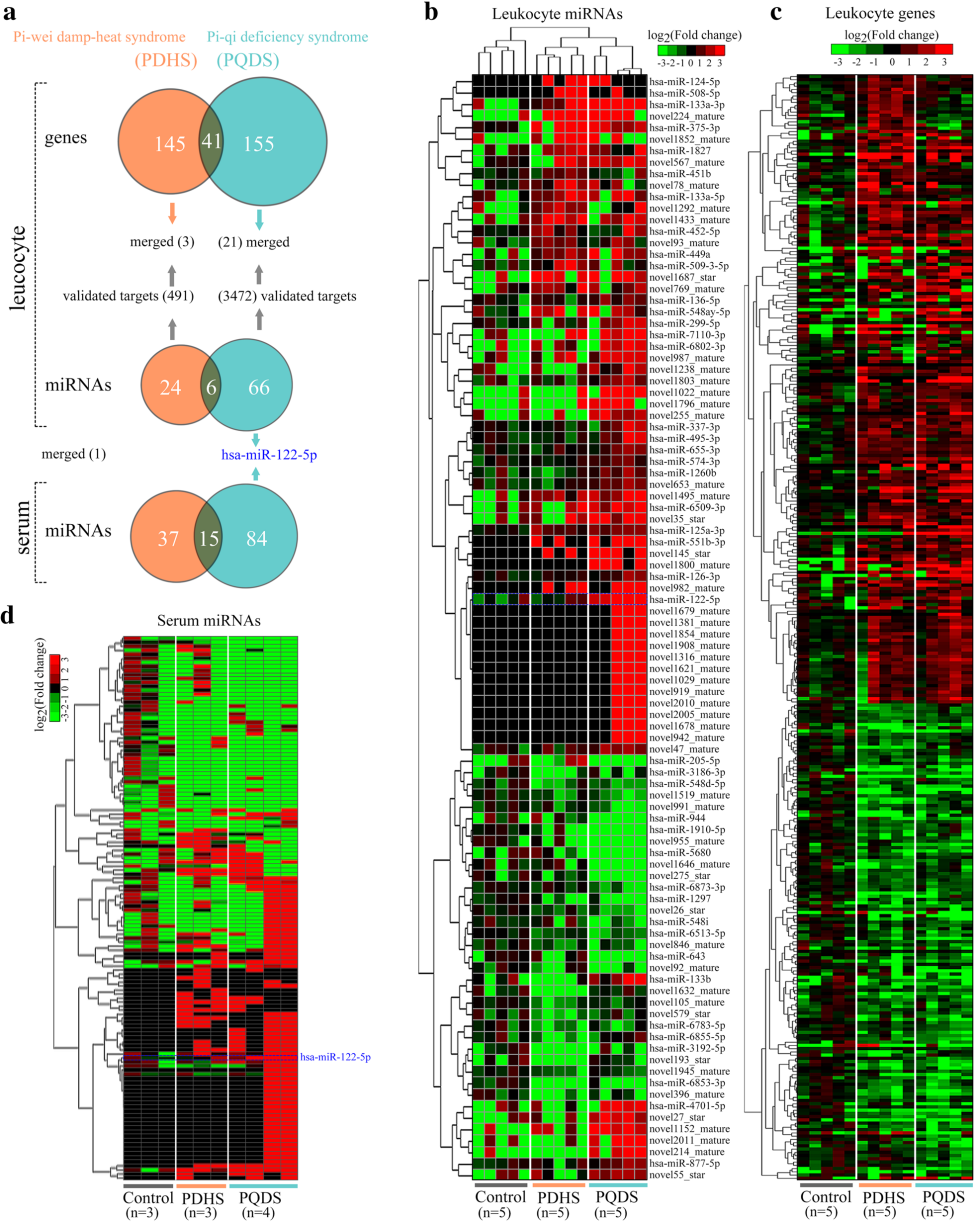
Expression pattern clustering analyses of the differential genes and miRNAs identified in the PDHS and PQDS populations. **a** Veen diagrams detailing the differential genes and miRNAs found in the leukocytes and serums. **b** hierarchical clustering (HCL) analyses of expression profiles of the differential miRNAs in leukocytes. **c** HCL analyses of expression profiles of the differential genes in leukocytes. **d** HCL analyses of expression profiles of the differential miRNAs in the serums. *Control* healthy individuals, *PQDS* chronic atrophic gastritis patients with Pi-qi-deficiency syndrome, *PDHS* chronic atrophic gastritis patients with Pi-wei damp-heat syndrome

Compared with the *control population* (healthy individuals, n = 5), 186 and 196 differential genes, as well as 30 and 72 differential miRNAs, were respectively identified in the leukocytes from two case populations, including the CAG patients with PDHS (n = 5) and CAG patients with PQDS (n = 5) (Fig. 1a; Additional file 1: Table S2-5). Besides, total 52 and 99 differential miRNAs were respectively found in the serums of the CAG patients with PDHS and PQDS (Fig. 1a; Additional file 1: Table S6 and 7). We particularly performed hierarchical clustering (HCL) expression analyses of the differential genes and miRNAs which were discovered in the leukocytes and serums from the three populations (Fig. 1b-d). The HCL showed that the expression profiles of differential miRNAs in the leukocytes of individuals in the same population clustered well together, distinguished from those in other populations (Fig. 1b). Interestingly, the miRNA termed hsa-miR-122-5p, was observed to be the common differential miRNAs in the leukocytes and serums of the CAG patients with PQDS (Fig. 1b and d).

### The *Zheng*-specific genes and miRNAs

The *Zheng*-specific genes in this study mean the differential genes and miRNAs which were observed in the individuals only with the TCM-defined PQDS or PDHS, excluding their common differential genes and miRNAs. Thus, the PQDS-specific genes and miRNAs were found only in the CAG patients with PQDS rather than PDHS. As indicated (Fig. 1a), 155 genes and 66 miRNAs (40 were novel), were PQDS-specific in the leukocytes, including the additional 84 PQDS-specific miRNAs (51 were novel) in the serums. Also, 145 genes and 24 miRNAs (12 were novel) were PDHS-specific in the leukocytes, and 37 PDHS-specific miRNAs (21 were novel) were discovered in the serums (Fig. 1a).

### Gene ontology functions of the *Zheng*-specific genes and miRNAs

**Fig. 2:**
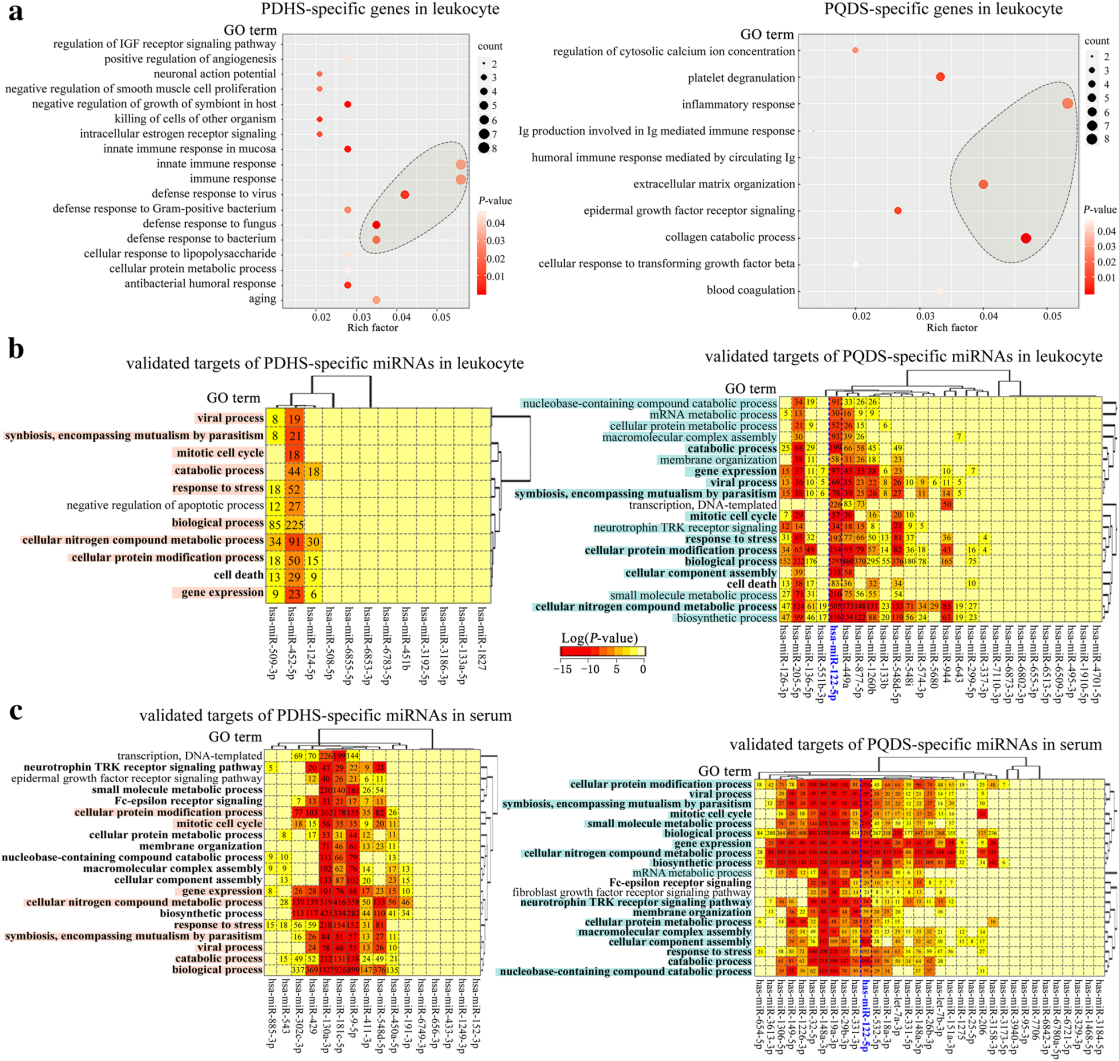
Gene ontology (GO) function enrichment analyses of the *Zheng*-specific genes and the targets of the *Zheng*-specific miRNAs discovered in the different case populations. **a** Bubble diagrams showing the enriched GO function terms of PDHS-specific genes (left) and PQDS-specific genes (right) in leukocytes. **b** Heatmaps indicating the GO function enrichment results of the validated targets of PDHS-specific miRNAs (left) and PQDS-specific miRNAs (right) in leukocytes. The common GO terms between the two *Zhengs* were shown in bold font. **c** Heatmaps displaying the enriched GO function terms of the validated targets of PDHS-specific miRNAs (left) and PQDS-specific miRNAs (right) in serums. The common GO terms between the two *Zhengs* were shown in bold font. The pink highlighted GO terms indicate the commonly enriched GO terms of the validated targets of the PDHS-miRNAs in the leukocytes and corresponding serums. The indigo highlighted GO terms mark the commonly enriched GO terms of the validated targets of the PQDS-miRNAs in the leukocytes and corresponding serums. *PQDS* chronic atrophic gastritis patients with Pi-qi-deficiency syndrome, *PDHS* chronic atrophic gastritis patients with Pi-wei damp-heat syndrome

The gene ontology (GO) function-based enrichment analyses were performed to investigate the possible functions of the above-mentioned *Zheng*-specific genes and miRNAs. As revealed (rich factor ≥ 0.035 & count ≥ 3), the PDHS-specific leukocyte genes were associated with the biological processes including defense response to virus, immune and innate immune response (Fig. 2a left). The PQDS-specific genes were mainly related to the biological processes such as inflammatory response, collagen catabolism and extracellular matrix (ECM) organization (Fig. 2a right).

Moreover, for the biological processes of the validated targets of *Zheng*-specific leukocyte miRNAs, it was observed that the validated targets of the PDHS-specific and PQDS-specific miRNAs could be implicated in the common biological processes including response to stress, gene expression, mitotic cell cycle, cell death, catabolic process, cellular protein modification process and cellular nitrogen compound metabolic process (Fig. 2b). The targets of the PDHS-specific leukocyte miRNAs were also involved in negative regulation of apoptotic process (Fig. 2b left). Notably, the targets of the PQDS-specific leukocyte miRNAs were enriched in more additional biological processes such as mRNA metabolism, neurotrophin biosynthesis, neurotrophin tyrosine kinase (TRK) receptor signaling, macromolecular complex assembly, membrane organization, nucleobase-containing compound catabolism, cellular protein metabolic process, small molecule metabolic process (Fig. 2b right).

In particular, for the biological processes enriched by the validated targets of *Zheng*-specific serum miRNAs, we discovered that the targets of the PDHS-specific or PQDS-specific serum miRNAs were also associated with the above-mentioned processes which were enriched by the corresponding PDHS-specific or PQDS-specific leukocyte miRNAs (Fig. 2b, c), but the PDHS-specific serum miRNAs seemed to be involved in more additional biological processes than the PDHS-specific leukocyte miRNAs (Fig. 2b, c left). These results suggested the potential common roles of *Zheng*-specific miRNAs in the leukocytes or serums in contributing to the characteristics and functions of leukocytes in the TCM-defined syndrome of PDHS and PQDS.

### Enriched pathways of the *Zheng*-specific genes and miRNAs

**Fig. 3:**
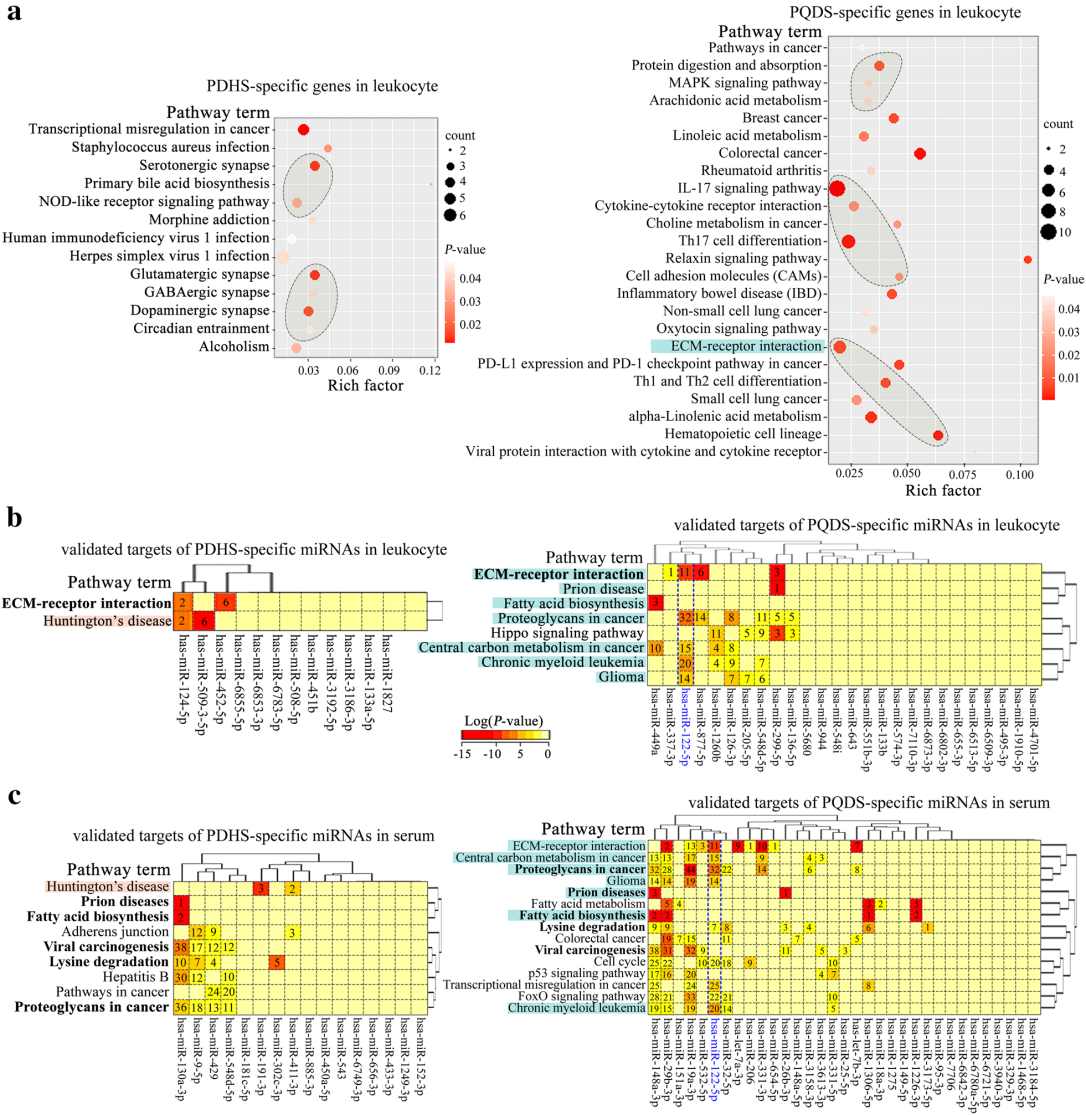
Pathway enrichment analyses of the *Zheng*-specific genes and the targets of the *Zheng*-specific miRNAs identified in the different case populations. **a** Bubble diagrams showing the enriched KEGG pathways of PDHS-specific genes (left) and PQDS-specific genes (right) in leukocytes. **b** Heatmaps indicating the KEGG pathway enrichment results of the validated targets of PDHS-specific miRNAs (left) and PQDS-specific miRNAs (right) in leukocytes. The common pathway terms were specially shown in bold font. **c** Heatmaps displaying the enriched KEGG pathways of the validated targets of PDHS-specific miRNAs (left) and PQDS-specific miRNAs (right) in serums. The common pathway terms were specially shown in bold font. The pink highlighted pathway terms indicate the commonly enriched pathways of the validated targets of the PDHS-miRNAs in leukocytes and serums. The indigo highlighted pathway terms mark the commonly enriched pathways of the validated targets of the PQDS-miRNAs in leukocytes and serums. *PQDS* chronic atrophic gastritis patients with Pi-qi-deficiency syndrome, *PDHS* chronic atrophic gastritis patients with Pi-wei damp-heat syndrome

The Kyoto Encyclopedia of Genes and Genomes (KEGG) pathway-based enrichment analyses were performed to decode the potential pathways of the *Zheng*-specific genes and miRNAs. The results showed that the PDHS-specific genes in the leukocytes were enriched in the pathways related to serotonergic, glutamatergic and dopaminergic synapse, including the nucleotide-binding oligomerization domain (NOD)-like receptor signaling pathway (Fig. 3a left; Additional file 1: Fig. S1–4). The PQDS-specific genes in the leukocytes were mainly involved in the pathways containing ECM-receptor interaction, cell adhesion molecules (CAMs), helper T (Th)1 and Th2 cell differentiation, Th17 cell differentiation, Interleukine (IL)17 signaling, cytokine-cytokine receptor interaction, mitogen-activated protein kinase (MAPK) signaling, arachidonic acid metabolism and protein digestion and absorption (Fig. 3a right; Additional file 1: Fig. S5–12).

In addition, concerning the enriched pathways of the validated targets of *Zheng*-specific miRNAs in the leukocytes, we found that the targets of the PDHS-specific and PQDS-specific miRNAs were both implicated in ECM-receptor interaction pathway (Fig. 3b), but the targets of the PQDS-specific miRNAs were involved in the additional pathways such as fatty acid biosynthesis, chronic myeloid leukemia, hippo signaling, proteoglycans in cancer and central carbon metabolism in cancer (Fig. 3b right).

Especially, regarding the potential pathways of the validated targets of *Zheng*-specific miRNAs in the serums, the targets of the PDHS-specific and PQDS-specific miRNAs were both associated with the pathways including fatty acid biosynthesis, lysine degradation and proteoglycans in cancer (Fig. 3c). The targets of PDHS-specific miRNAs were also involved in other pathways related to adherent junction and cancer (Fig. 3c left). The targets of PQDS-specific miRNAs were specially implicated in several additional pathways such as ECM-receptor interaction, fatty acid metabolism, forkhead box protein O (foxO) signaling, chronic myeloid leukemia, cell cycle, p53 signaling, central carbon metabolism in cancer, colorectal cancer and transcriptional misregulation in cancer (Fig. 3c right). Interestingly, the targets of the PQDS-specific serum miRNAs were enriched in more pathways, covering almost all the enriched pathways of the targets of PQDS-specific leukocyte miRNAs (Fig. 3b and c right), which suggested the possible common roles of these miRNAs in contributing to the characteristics and functions of leukocytes in the CAG patients with PQDS.

### Interaction networks of Zheng-specific genes

**Fig. 4:**
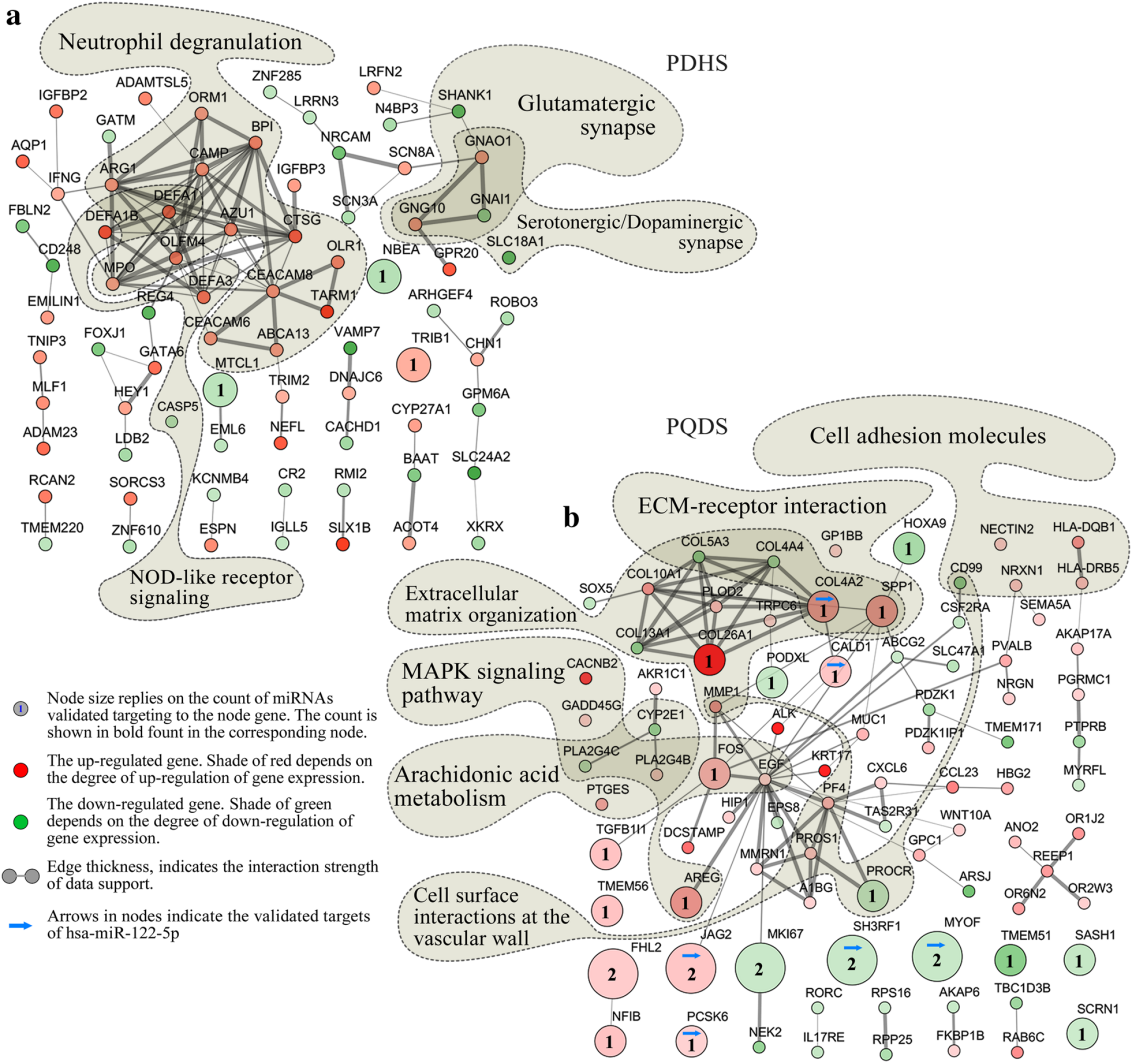
Interaction network analyses of the PDHS-specific and PQDS-specific genes in leukocytes. **a** The network detailing the interactions of PDHS-specific genes. **b** The network generated to show interactions of PQDS-specific genes. The number marked in a node indicates the count of the PQDS-specific or PDHS-specific miRNAs which were validated targeting to the corresponding node gene. The node genes-enriched pathways were specially marked in the interaction networks. *PQDS* chronic atrophic gastritis patients with Pi-qi-deficiency syndrome, *PDHS* chronic atrophic gastritis patients with Pi-wei damp-heat syndrome

The interaction networks of the *Zheng*-specific leukocyte genes were carefully created (Fig. 4). The generated networks not only detail the possible interactions between each node gene (edge thickness indicates interaction strength of data support), but also visually present the expression pattern of each node gene (node shade of green or red relies on degree of down-regulation or up-regulation of gene expression). Node size depends on the number of miRNAs which were experimentally validated targeting to the corresponding node gene, and the number is particularly displayed in the corresponding gene node. Especially, several enriched pathways were highlighted and annotated in the resultant networks. As shown (Fig. 4a; Additional file 1: Fig. S13; Additional file 1: Table S8), several pathways, including neutrophil degranulation (reactome), NOD-like receptor signaling, serotonergic synapse, dopaminergic synapse and glutamatergic synapse, were specially marked in the interaction network of the PDHS-specific genes. Obviously, 15 up-regulated genes were enriched in neutrophil degranulation pathway, indicating the active and enhanced neutrophil degranulation in the CAG patients with TCM-defined PDHS. Each of the genes, *NBEA* (neurobeachin), *MTCL1* (microtubule cross-linking factor 1) and *TRIB1* (TRIBbles homolog 1), kept a corresponding PDHS-specific miRNA regulator.

In addition, in the interaction network of the PQDS-specific genes (Fig. 4b; Additional file 1: Fig. S14; Additional file 1: Table S9), several genes were related to arachidonic acid metabolism pathway. In particular, the genes keeping more complex interaction relationships with each other, were specially enriched in MAPK signaling pathway and Th cell differentiation pathway, especially the pathways implicated in cell-to-cell adhesion/junction and communication such as CAMs, ECM-receptor interaction, ECM-organization and cell surface interactions at the vascular wall. Notably, the genes for the cross-talking between these pathways, including *COL4A2* (collagen, type IV, alpha 2), *COL26A1* (collagen, type XXVI, alpha 1), *SPP1* (secreted phosphoprotein 1), *FOS* (proto-oncogene c-Fos) and *PROCR*, underwent regulation of the PQDS-specific leukocyte miRNAs. These results suggested that the PQDS-specific miRNAs had potential roles in the regulation of cell-to-cell adhesion/junction and communication, contributing to the characteristics and functions of leukocytes in the CAG patients with TCM-defined PQDS.

### The *Zheng*-specific miRNA-gene interactions

**Fig. 5:**
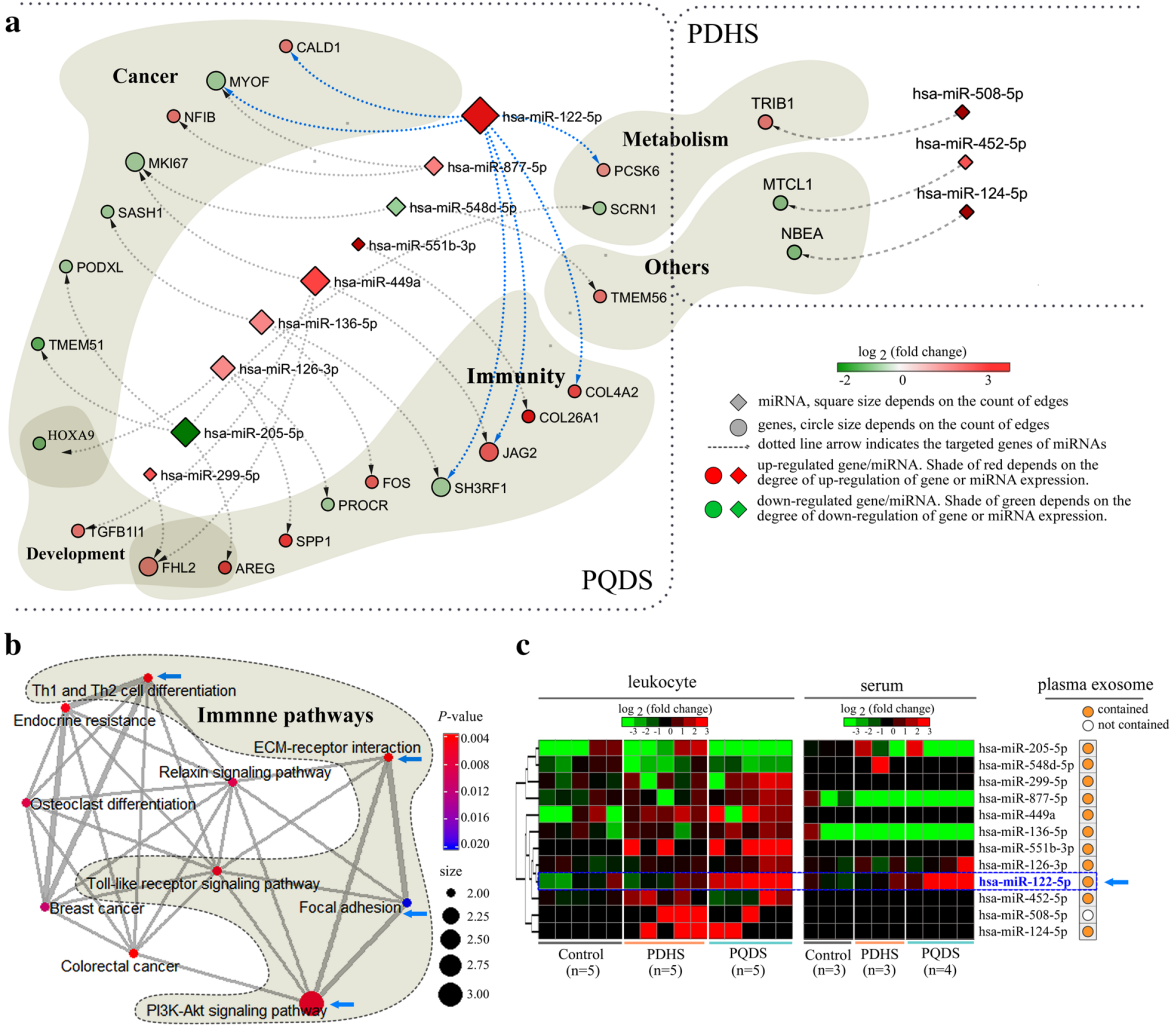
Function and pathway analyses for the experimentally supported miRNA-gene interactions in the leukocytes from the PQDS and PDHS populations. **a** The network detailing the PQDS-specific and PDHS-specific miRNA-gene interactions in leukocytes. The target genes having similar functions were specially marked and grouped together. The expression levels of miRNAs and genes were visually presented in the form of colorful nodes showing the color change (green to red) in brightness and chromaticity. **b** The relationship network of the enriched pathways of genes belonging to the PQDS-specific miRNA-gene interaction pairs. The edge between two nodes indicate there are several common genes implicated in the nodes-labeled pathways, thus the edge thickness depends on the number of the existed common genes. The blue arrows denoted the immune pathways containing the validated targets of the has-miR-122-5p. **c** Clustering analyses of the expression profiles of the miRNAs belonging to the experimentally supported miRNA-gene interaction pairs in different populations. Two heatmaps were generated to profile the miRNAs expression in the leukocytes and serums of individuals from different populations. Especially, if there are additional experimental evidences supporting that a listed miRNA can be contained and carried in the plasma exosomes, it was thus specially marked with an orange circle. *Control* healthy individuals, *PQDS* chronic atrophic gastritis patients with Pi-qi-deficiency syndrome, *PDHS* chronic atrophic gastritis patients with Pi-wei damp-heat syndrome

Based on the *Zheng*-specific genes and miRNAs discovered in this work (Fig. 1a), especially the experimentally-supported miRNA-gene interactions, the interaction networks were particularly generated to detail the *Zheng*-specific miRNA-gene interaction pairs and visually presented the expression patterns of miRNA and gene in each interaction pair (Fig. 5a). As shown, three miRNA-gene interaction pairs were contained in the PDHS-specific miRNA-gene interaction network (Fig. 5a right; Additional file 1: Table S10), but there were 21 miRNA-gene interaction pairs for the PQDS-specific miRNA-gene interaction network (Fig. 5a left; Additional file 1: Table S11). Based on the gene functional information from the *GeneCards* human gene database (https://www.genecards.org), we found that the PQDS-specific miRNA-gene pairs seemed to be implicated in immunity, cancer, development and mentalism (Fig. 5a left). The further pathway enrichment analyses also indicated that these PQDS-specific miRNA-gene interactions could link with the pathways related to cancer and immunity, and notably there was multiple crosstalk mediated by the common targets among these pathways (Fig. 5b). The enriched pathways included the immune pathways, such as Th1 and Th2 cell differentiation, toll-like receptor signaling, and PI3K (phosphatidylinositol 3-kinase)-Akt (serine/threonine-protein kinase) signaling. Especially, the focal adhesion and ECM-receptor interaction pathways were involved in cell-to-cell adhesion/junction and communication, probably contributing to the characteristics and functions of leukocytes in the CAG patients with TCM-defined PQDS.

In addition, for the miRNAs belonging to the miRNA-gene interaction pairs, two heatmaps were specially generated to profile their expression in the leukocytes and serums of individuals from different populations. Particularly, using the ExoCarta exosome database [12], we analyzed weather these miRNAs could be contained and carried in the plasma exosome (Fig. 5c). As indicated, although the *Zheng*-specific leukocyte miRNAs kept high levels in the leukocytes, they almost couldn’t be found in the corresponding serums. Interestingly, hsa-miRNA-122-5p, a PQDS-specific miRNA discovered both in leukocytes and serums (Fig. 1a), kept much higher expression both in leukocytes and the corresponding serums (Fig. 5c). It could target to the PQDS-specific leukocyte genes in the above-mentioned immune pathways, including the focal adhesion and ECM-receptor interaction pathways related to cell-to-cell adhesion/junction and communication (Fig. 5b). Notably, there were additional experimental evidences supporting that it could be encapsulated and carried in the plasma exosomes, suggesting it could function as a regulator of genes in the far away recipient cells throughout the body.

### Potential functional analyses of the exosome-contained hsa-miR-122-5p

**Fig. 6:**
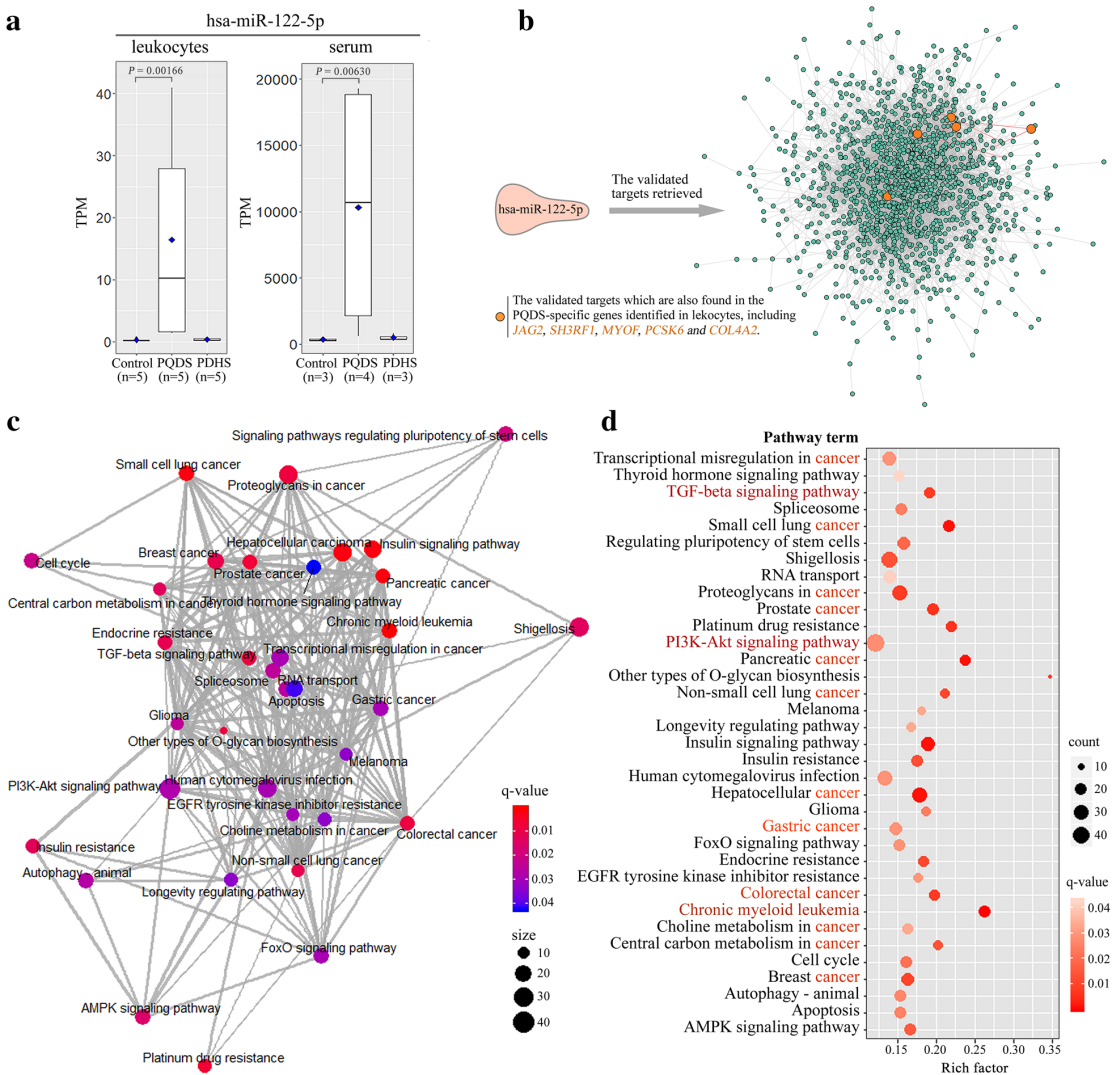
Function and pathway analyses of the validated targets of the exosome-carried hsa-miR-122-5p. **a** Boxplot graphs of expression levels of hsa-miR-122-5p in the leukocytes and serums from different populations. **b** Overview of the interaction network of targets of hsa-miR-122-5p. The invalidated targets of has-miR-122-5p were retrieved from the released “TarBase” collecting the experimentally supported miRNA-gene interactions, both in vivo and in vitro. Especially, the orange nodes show the validated targets that are also found in the PQDS-specific genes identified in the leukocytes, including *JAG2*, *SH3RF1*, *MYOF*, *PCSK6* and *COL4A2*. **c** The relationship network of the enriched pathways of targets of hsa-miR-122-5p. A node labeled with a pathway term, indicates an enriched pathway of targets, and the node size depends on the number of edges linking the node. The edge between two nodes denotes that there exist several common genes involved in the two nodes-labeled pathways, so the edge thickness relies on the number of the existed common genes. **d** The bubble diagram generated to show the enriched pathways of the validated targets of hsa-miR-122-5p. *TPM* transcripts per million reads, *Control* healthy individuals, *PQDS* chronic atrophic gastritis patients with Pi-qi-deficiency syndrome, *PDHS* chronic atrophic gastritis patients with Pi-wei damp-heat syndrome

The PQDS-specific miRNAs, hsa-miRNA-122-5p, was capable of being encapsulated and carried in plasma exosomes, especially keeping much higher levels both in leukocytes and serums (Figs. 5c and 6a). The plasma exosomes could transfer it into other far away recipient cells, making it function all over the body. In order to further investigate their potential roles, we specially retrieved its target genes from the released TarBase v7.0, a database collecting the experimentally supported miRNA-gene interactions, both in vitro and in vivo [16, 22]. About 1524 targets were obtained, containing 100 direct target genes (Additional file 1: Table S12), and an interaction network was generated to overview the possible interactions among them (Fig. 6b). Notably, the node genes having more interaction relationships with other genes were just the previously-mentioned PQDS-specific leukocyte genes, including *JAG2* (jagged-2), *COL4A2*, *SH3RF1* (E3 ubiquitin-protein ligase, SH3 domain containing ring finger 1), *MYOF* (Myoferlin) and *PCSK6* (proprotein convertase subtilisin/kexin type 6) (Figs. 5a and 6b). Furthermore, the pathway enrichment analyses of all the obtained targets revealed the potential pathways implicated in multiple human cancers, including the gastric and colorectal cancers of digestive system, and especially the gastric cancer was usually correlated with CAG. Besides, the enriched pathways were also involved in metabolism, autophagy, apoptosis and cell cycle (Fig. 6d). The pathways associated with transforming growth factor (TGF)-beta signaling and chronic myeloid leukemia, indicated the exosome-contained has-miR-122-5p might have potential roles in the regulation of leukocyte proliferation. Especially, the PI3K-Akt signaling pathway containing much more targets of hsa-miR-122-5p, kept multiple crosstalk mediated by the common targets with other pathways, including the focal adhesion and ECM-receptor interaction pathways implicated in cell-to-cell adhesion/junction and communication (Figs. 5b and 6c).

## Discussion

TCM was developed through thousands of years of empirical testing and refinement. *Zheng*, meaning syndrome, is a thousand-year-old key diagnostic concept in TCM [4, 23, 24]. Interestingly, two TCM-defined syndromes, PQDS and PDHS, seemed to be the commonly occurring *Zhengs* among CAG patients [10, 13, 20, 26]. Leukocytes, as the important immune cells throughout the body, have very crucial roles in host defense and contribute to pathogenesis of various immune diseases. Hence, the changes in the characteristics and functions of leukocytes may be implicated in the two TCM-defined *Zheng*s of CAG. Based on the high throughput identification of expression profiles of genes and miRNAs in leukocytes, including the circulating miRNAs in serums, we especially wanted to decode the miRNA-mediated gene expression underlying the leukocyte characteristics and functions under the two TCM-defined *Zheng*s resulting from CAG, in particular the potential miRNA biomarker candidates in the corresponding serums.

Compared with the healthy control, the *Zheng*-specific genes and miRNAs identified in leukocytes were different for the two TCM syndromes of CAG, and the *Zheng*-specific miRNAs in the corresponding serums were also different (Fig. 1a). Despite being the TCM-defined resultant *Zheng*s resulting from the same disease of CAG, the *Zheng*-specific genes and miRNAs were not same. Because gene expression determines cell’s characteristics, the *Zheng*-specific gene expression in leukocytes may induce the specific alterations in the characteristics and functions of leukocytes in the two TCM-defined *Zheng*s. Function enrichment analyses showed that the PDHS-specific genes in leukocytes were mainly involved in the biological processes related to defense and immune response (Fig. 2a left), but the PQDS-specific genes in leukocytes were implicated in the processes such as inflammatory response, extracellular matrix organization and collagen catabolism (Fig. 2a right). Furthermore, the PDHS-specific genes were enriched in the pathways including neutrophil degranulation (reactome), NOD-like receptor signaling, serotonergic synapse, glutamatergic synapse and dopaminergic synapse (Figs. 3a and 4a). The PQDS-specific genes were implicated in the pathways containing protein digestion and absorption, arachidonic acid metabolism, MAPK signaling, IL17 signaling and Th cell differentiation, especially the pathways associated with cell-to-cell adhesion/junction and communication such as ECM-receptor interaction, cell adhesion molecules and extracellular matrix organization (Figs. 3b and 4b). Hence, the expression upregulation of the PDHS-specific genes enriched in the neutrophil degranulation pathway, indicated the enhanced leukocyte activation in the PDHS of CAG. Notably, the PQDS-specific genes could be involved in the pathways related to cell-to-cell adhesion/junction and communication, probably contributing to the alterations in the characteristics and functions of leukocytes in the PQDS of CAG. Four of the validated PQDS-specific miRNA-gene interaction pairs, concerned with the targets of *COL4A2*, *COL26A1*, *SPP1* and *PROCR*, were implicated in the regulation of the pathways associated with cell-to-cell adhesion/junction and communication, suggesting the potential roles of the PQDS-specific miRNAs in contributing to the changes in characteristics and functions of leukocytes in the TCM-defined PQDS of CAG (Figs. 4b and 5a).

In addition, to detail the miRNA-mediated gene expression underlying leukocyte characteristics and functions, total 21 pairs of PQDS-specific miRNA-gene interactions were identified in leukocytes, related to regulation of immunity, cancer, development and metabolism (Fig. 5a). They could link to the immune and cancer pathways which kept multiple crosstalk mediated by the common target genes. These linked immune pathways included Th1 and Th2 cell differentiation, toll-like receptor signaling and PI3K-Akt signaling. Specifically, the focal adhesion and ECM-receptor interaction pathways could be implicated in cell-to-cell adhesion/junction and communication, probably leding to the changes in characteristics and functions of leukocytes in the TCM-defined PQDS of CAG (Fig. 5b). However, only three pairs of PDHS-specific miRNA-gene interaction pairs were observed in the leukocytes, possibly because the current datasets from the released Tarbase (a database collecting the experimentally supported miRNA-target interactions) was limited [6, 16]. The three obtained PDHS-specific miRNAs targeting to *TRIB1*, *MTCL1* and *NBEA*, were concern with metabolism and other functions (Fig. 5a). These results suggested again that the *Zheng*-specific miRNAs seemed to play potential roles in the regulation of the *Zheng*-specific genes expression, further contributing to the characteristics and functions of leukocytes in the TCM-defined *Zheng*s of CAG.

Regarding to the *Zheng*-specific miRNAs in the corresponding serums (Fig. 1a), we found that the validated targets of the *Zheng*-specific serum miRNAs were also associated with the biological processes enriched by the corresponding *Zheng*-specific leukocyte miRNAs (Fig. 2b and c). Besides, the PDHS-specific and PQDS-specific serum miRNAs were both associated with the pathways including fatty acid biosynthesis, lysine degradation and proteoglycans in cancer (Fig. 3c). The PDHS-specific serum miRNAs were also involved in other pathways related to adherent junction and cancer (Fig. 3c left). The PQDS-specific serum miRNAs were specially implicated in several additional pathways such as ECM-receptor interaction, fatty acid metabolism, FoxO signaling, chronic myeloid leukemia, cell cycle, p53 signaling, central carbon metabolism in cancer, colorectal cancer and transcriptional misregulation in cancer (Fig. 3c right). Interestingly, the targets of PQDS-specific serum miRNAs could be enriched in more pathways, covering almost all the enriched pathways of the targets of PQDS-specific leukocyte miRNAs (Fig. 3b and c right). Thereby, these PQDS-specific miRNAs in the serums and the corresponding leukocytes, seemed to have the common roles in contributing to the characteristics and functions of leukocytes in the TCM-defined PQDS of CAG.

Especially, the PQDS-specific miRNA, has-miR-122-5p, was identified both in leukocytes and serums (Fig. 1a), keeping much higher levels both in the leukocytes and the corresponding serums (Figs. 5c and 6a). Importantly, because it was capable of being encapsulated and carried in plasma exosomes, the exosomes could transfer it into other far away recipient cells, making it function all over the body (Fig. 5c). About 1524 validated targets were obtained from the Tarbase [6, 16], containing the PQDS-specific leukocyte genes (*JAG2*, *COL4A2*, *SH3RF1*, *MYOF* and *PCSK6*) that kept multiple interactions with other targets (Figs. 5a and 6b). These targets could be involved in multiple human cancers, including the gastric and colorectal cancers of digestive system, and especially the gastric cancer that was usually correlated with CAG (Fig. 6c and d). They were also implicated in the pathways related to metabolism, autophagy, apoptosis and cell cycle. The enriched pathways of TGF-beta signaling and chronic myeloid leukemia, indicated the exosome-carried has-miR-122-5p might have potential roles in regulating leukocyte proliferation. Notably, PI3K-Akt signaling pathway containing much more targets of hsa-miR-122-5p, held multiple crosstalk mediated by the common targets with other pathways associated with cell-to-cell adhesion/junction and communication, such as focal adhesion and ECM-receptor interaction (Figs. 5b and 6d). Interestingly, despite being the TCM-defined resultant *Zheng*s resulting from the same disease of CAG, the hsa-miR-122-5p levels were specifically higher both in the leukocytes and serums of individuals from the PQDS population rather than the PDHS population (Figs. 5c and 6a). These results suggested hsa-miR-122-5p could be a potential biomarker candidate for the TCM-defined PQDS of CAG.

The RNA/miRNA-seq analyses of leukocytes and serums revealed miRNA-mediated gene expression contributing to the leukocyte characteristics and functions under the TCM-defined resultant *Zheng*s (PQDS and PDHS) of CAG, including the potential serum miRNA biomarker candidates. Although PQDS and PDHS seemed to be the commonly occurring *Zhengs* among CAG patients, there were other three resultant *Zhengs* which were also resulted from the same disease of CAG [2, 26]. Thus, the observed specific patterns to differentiate the resultant PQDS and PDHS of CAG, were unlikely to be suitable for differentiating them from other resultant *Zhengs* of CAG. Besides, the study population was not large enough to draw the definitive conclusions, and there were significant differences in age between the health control group and CAG patients which may lead to biased conclusions. Hence, further studies involving more resultant *Zhengs* of CAG and larger sample sizes, are needed to strengthen the conclusions of this study.

## Conclusions

Despite being the two TCM-defined resultant *Zheng*s resulting from the same disease of CAG, there seemed to be different changes in characteristics and functions of leukocytes in the two TCM *Zheng*s of PDHS and PQDS. The *Zheng*-specific miRNAs seemed to play potential roles in the regulation of the *Zheng*-specific genes expression, further contributing to the characteristics and functions of leukocytes in the TCM-defined *Zheng*s of CAG. Especially, the PQDS-specific miRNAs in the serums and the corresponding leukocytes, seemed to have the common roles in contributing to the characteristics and functions of leukocytes in the PQDS of CAG. Importantly, hsa-miR-122-5p, specifically higher expression both in the leukocytes and corresponding serums in the PQDS rather than the PDHS, could be a potential biomarker candidate for the TCM-defined PDHS of CAG. These results may provide new insights into the characteristic and functional changes of leukocytes in the two TCM *Zheng*s, especially the miRNA-mediated gene regulation underlying leukocyte characteristics and functions, with potential leukocyte and serum biomarkers for future application in integrative medicine.

## Supplementary Information

**Additional file 1: Table S1.** List of leukocytes and serum samples from the clinical participants. **Table S2.** List of the differentially expressed genes identified in blood leukocytes in PDHS. **Table S3.** List of the differentially expressed genes identified in blood leukocytes in PQDS. **Table S4.** List of the differentially expressed miRNAs in blood leukocytes in PDHS. **Table S5.** List of the differentially expressed miRNAs identified in blood leukocytes in PQDS. **Table S6.** List of the differentially expressed miRNAs in serums in PDHS. **Table S7.** List of the differentially expressed miRNAs in serum in PQDS. **Table S8.** The detailed interaction relationships among the PDHS-specific genes. **Table S9.** The detailed interaction relationships among the PQDS-specific genes. **Table S10.** The experimental evidences supporting the PDHS-specific miRNA-gene interaction pairs. **Table S11.** The experimental evidences supporting the PQDS-specific miRNA-gene interaction pairs. **Table S12.** The experimentally-supported direct targets of the exosome-contained has-miR-122-5p. **Figure S1.** Glutamatergic synapse pathway (hsa04724). **Figure S2.** Serotonergic/Dopaminergic synapse pathway (hsa04726). **Figure S3.** Dopaminergic synapse pathway (hsa04728). **Figure S4.** NOD-like receptor signaling pathway (hsa04621). **Figure S5.** ECM-receptor interaction (hsa04512). **Figure S6.** Cell adhesion molecules (CAMs) (hsa04514). **Figure S7.** MAPK signaling pathway (hsa04010). **Figure S8.** Th1 and Th2 cell differentiation (hsa04658). **Figure S9.** IL-17 signaling pathway (hsa04657). **Figure S10.** Th17 cell differentiation pathway (hsa04659). **Figure S11.** Cytokine-cytokine receptor interaction pathway (hsa04060). **Figure S12.** Arachidonic acid metabolism (hsa590). **Figure S13.** The network detailing the interaction relationships of the PDHS-specific genes in leukocytes. **Figure S14.** The network detailing the interaction relationships of the PQDS-specific genes in leukocytes.

## Data Availability

All sequence data have been deposited in GenBank under BioProject accession number PRJNA591186. The RNA-seq and miRNA-seq reads are deposited in the NCBI Sequence Read Archive (SRA) (http://www.ncbi.nlm.nih.gov/sra) under the accession numbers (SRR10513209, SRR10513208, SRR10513204, SRR10513203, SRR10513202, SRR11548312, SRR11548311, SRR11548330, SRR11548319, SRR11548318, SRR11548317, SRR11548316, SRR11548315, SRR11548314, SRR11548313, SRR11483205, SRR11483204, SRR11483203, SRR11483202, SRR11483201, SRR11548310, SRR11548339, SRR11548338, SRR11548337, SRR11548336, SRR11548335, SRR11548334, SRR11548333, SRR11548332, SRR11548331, SRR11548322, SRR11548321, SRR11548320, SRR11548329, SRR11548328, SRR11548327, SRR11548326, SRR11548325, SRR11548324 and SRR11548323).

## Abbreviations

TCM: Traditional Chinese medicine;
mRNA: Messenger RNA
miRNA: MicroRNA
PQDS: Pi-qi-deficiency syndrome
PDHS: Pi-wei damp-heat syndrome
CAG: Chronic atrophic gastritis
NOD: Nucleotide-binding-oligomerisation-domains
CAM: Cell adhesion molecules
ECM: Extracellular matrix
MAPK: Mitogen-activated protein kinase
Th: Helper T
COL4A2: Collagen, type IV, alpha 2
COL26A1: Collagen, type XXVI, alpha 1
SPP1: Secreted phosphoprotein 1
PROCR: Endothelial protein C receptor
NGS: Next-generation sequencing
TPM: Transcripts per million
FPKM: Fragments per kilobase of exon model per million mapped reads
HCL: Hierarchical clustering
GO: Gene Ontology
KEGG: Kyoto Encyclopedia of Genes and Genomes
TRK: Tyrosine kinase
foxO: Forkhead box protein O
NBEA: Neurobeachin
MTCL1: Microtubule cross-linking factor 1
TRIB1: TRIBbles homolog 1
FOS: Proto-oncogene c-Fos
PI3K: Phosphatidylinositol 3-kinase
Akt: Serine/threonine-protein kinase
JAG2: Jagged-2
SH3RF1: E3 ubiquitin-protein ligase, SH3 domain containing ring finger 1
MYOF: Myoferlin
PCSK6: Proprotein convertase subtilisin/kexin type 6
TGF: Transforming growth factor

## References

[1] Buchan JR, Parker R (2007). Molecular biology. The two faces of miRNA. Science (New York, NY)..

[2] Cao ZJ, Zuo MH (2018). Study on relationship between traditional Chinese medicine syndrome distribution, gastroscopy and pathology in chronic atrophic gastritis. World Chin Med.

[3] Chen K, Rajewsky N (2007). The evolution of gene regulation by transcription factors and microRNAs. Nat Rev Genet.

[4] Cheng F, Wang X, Song W, Lu Y, Li X, Zhang H (2014). Biologic basis of TCM syndromes and the standardization of syndrome classification. J Trad Chin Med Sci.

[5] Cheung F (2011). TCM: Made in China. Nature.

[6] Chou CH, Chang NW, Shrestha S, Hsu SD, Lin YL, Lee WH (2016). miRTarBase 2016: updates to the experimentally validated miRNA-target interactions database. Nucleic Acids Res.

[7] de Hoon MJ, Imoto S, Nolan J, Miyano S (2004). Open source clustering software. Bioinformatics (Oxford, England).

[8] Fang JY, Liu WZ, Li ZK, Du YQ, Ji XL, Ge ZZ (2013). China Chronic Gastritis Consensus (2012, Shanghai). Chin J Front Med Sci..

[9] Hasin Y, Seldin M, Lusis A (2017). Multi-omics approaches to disease. Genome Biol.

[10] Hu L, Zheng XF, Yan XH (2012). Expressions of HSP 70 and NF-kappaB in the peripheral blood lymphocyte of chronic gastritis patients of different syndrome patterns. Chin J Integr Trad West Med.

[11] da Huang W, Sherman BT, Lempicki RA (2009). Bioinformatics enrichment tools: paths toward the comprehensive functional analysis of large gene lists. Nucleic Acids Res.

[12] Huang X, Yuan T, Tschannen M, Sun Z, Jacob H, Du M (2013). Characterization of human plasma-derived exosomal RNAs by deep sequencing. BMC genomics.

[13] Liang JK, Hu L, Zheng XF (2012). Study of Th1/Th2 balance in peripheral blood of chronic gastritis patients with Pi-Wei damp-heat syndrome. Chin J Integr Trad West Med..

[14] Love MI, Huber W, Anders S (2014). Moderated estimation of fold change and dispersion for RNA-seq data with DESeq2. Genome Biol.

[15] Metwaly A, Haller D. Multi-omics in IBD biomarker discovery: the missing links. Nat Rev Gastroenterol Hepatol. 2019. 10.1038/s41575-019-0188-9 31312043

[16] Paraskevopoulou MD, Vlachos IS, Hatzigeorgiou AG (2016). DIANA-TarBase and DIANA Suite Tools: Studying Experimentally Supported microRNA Targets. Curr Prot Bioinform.

[17] Rashad NM, El-Shal AS, Shalaby SM, Mohamed SY (2018). Serum miRNA-27a and miRNA-18b as potential predictive biomarkers of hepatitis C virus-associated hepatocellular carcinoma. Mol Cell Biochem.

[18] Shannon P, Markiel A, Ozier O, Baliga NS, Wang JT, Ramage D (2003). Cytoscape: A software environment for integrated models of biomolecular interaction networks. Genome Res.

[19] Szklarczyk D, Gable AL, Lyon D, Junge A, Wyder S, Huerta-Cepas J (2019). STRING v11: Protein-protein association networks with increased coverage, supporting functional discovery in genome-wide experimental datasets. Nucleic Acids Res.

[20] Tang XD, Lu B, Zhou LY, Zhan SY, Li ZH, Li BS (2012). Clinical practice guideline of Chinese medicine for chronic gastritis. Chin J Integr Med.

[21] Vlachos IS, Hatzigeorgiou AG (2017). Functional Analysis of miRNAs Using the DIANA Tools Online Suite. Methods Mol Biol.

[22] Vlachos IS, Paraskevopoulou MD, Karagkouni D, Georgakilas G, Vergoulis T, Kanellos I (2015). DIANA-TarBase v7.0: indexing more than half a million experimentally supported miRNA:mRNA interactions. Nucleic Acids Res..

[23] Wang T, Dong J (2017). What is “zheng” in traditional Chinese medicine?. J Trad Chin Med Sci.

[24] Wang Y, Xu A (2014). Zheng: A systems biology approach to diagnosis and treatments. Science (New York, NY).

[25] Xie C, Mao X, Huang J, Ding Y, Wu J, Dong S (2011). KOBAS 20: a web server for annotation and identification of enriched pathways and diseases. Nucleic Acids Res.

[26] Zhang SS, Tang XD, Huang HP, Bian LQ (2017). Expert consensus on diagnosis and treatment of Traditional Chinese Medicine for chronic gastritis (2017). China J Trad Chin Med Pharm..

[27] Zhang Y, Wen X, Hu XL, Cheng LZ, Yu JY, Wei ZB (2016). Downregulation of miR-145-5p correlates with poor prognosis in gastric cancer. Eur Rev Med Pharm Sci.

[28] Zheng B, Jeong SS, Zhu YJ, Chen L, Xia Q (2017). miRNA and lncRNA as biomarkers in cholangiocarcinoma(CCA). Oncotarget.

[29] Zheng XY (2002). Guiding principle for clinical research on new drugs of traditional Chinese medicine.

