# Supplementary material for: Integrated analyses of miRNA and mRNA profiles in leukocytes and serums in traditional Chinese medicine (TCM)-defined Pi-qi-deficiency syndrome and Pi-wei damp-heat syndrome resulting from chronic atrophic gastritis
Source: Chin Med. 2021 Jan 6;16:4. doi: 10.1186/s13020-020-00416-9 (PMC7788792; doi:10.1186/s13020-020-00416-9)
Supplement: Supplementary file 1 — Additional file 1: Table S1. List of leukocytes and serum samples from the clinical participants. Table S2. List of the differentially expressed genes identified in blood leukocytes in PDHS. Table S3. List of the differentially expressed genes identified in blood leukocytes in PQDS. Table S4. List of the differentially expressed miRNAs in blood leukocytes in PDHS. Table S5. List of the differentially expressed miRNAs identified in blood leukocytes in PQDS. Table S6. List of the differentially expressed miRNAs in serums in PDHS. Table S7. List of the differentially expressed miRNAs in serum in PQDS. Table S8. The detailed interaction relationships among the PDHS-specific genes. Table S9. The detailed interaction relationships among the PQDS-specific genes. Table S10. The experimental evidences supporting the PDHS-specific miRNA-gene interaction pairs. Table S11. The experimental evidences supporting the PQDS-specific miRNA-gene interaction pairs. Table S12. The experimentally-supported direct targets of the exosome-contained has-miR-122-5p. Figure S1. Glutamatergic synapse pathway (hsa04724). Figure S2. Serotonergic/Dopaminergic synapse pathway (hsa04726). Figure S3. Dopaminergic synapse pathway (hsa04728). Figure S4. NOD-like receptor signaling pathway (hsa04621). Figure S5. ECM-receptor interaction (hsa04512). Figure S6. Cell adhesion molecules (CAMs) (hsa04514). Figure S7. MAPK signaling pathway (hsa04010). Figure S8. Th1 and Th2 cell differentiation (hsa04658). Figure S9. IL-17 signaling pathway (hsa04657). Figure S10. Th17 cell differentiation pathway (hsa04659). Figure S11. Cytokine-cytokine receptor interaction pathway (hsa04060). Figure S12. Arachidonic acid metabolism (hsa590). Figure S13. The network detailing the interaction relationships of the PDHS-specific genes in leukocytes. Figure S14. The network detailing the interaction relationships of the PQDS-specific genes in leukocytes. [file 13020_2020_416_MOESM1_ESM.docx]

**Additional materials**

You L, Zhang S, *et al*. Integrated analyses of miRNA and mRNA profiles in leukocytes and serums in traditional Chinese medicine (TCM)-defined Pi-qi-deficiency syndrome and Pi-wei damp-heat syndrome resulting from chronic atrophic gastritis.

**Contents**

A[**dditional methods** 2](#_Toc47107805)

[**Inclusion and exclusion criteria for participants** 2](#_Toc47107806)

[**The experimental design and route** 3](#_Toc47107807)

[**miRNA sequencing** 4](#_Toc47107808)

[**RNA sequencing** 4](#_Toc47107809)

**A**[**dditional Tables** 5](#_Toc47107810)

[**Table S1** List of leukocytes and serum samples from the clinical participants. 5](#_Toc47107811)

[**Table S2** List of the differentially expressed genes identified in blood leukocytes in PDHS. 6](#_Toc47107812)

[**Table S3** List of the differentially expressed genes identified in blood leukocytes in PQDS. 11](#_Toc47107813)

[**Table S4** List of the differentially expressed miRNAs in blood leukocytes in PDHS. 16](#_Toc47107814)

[**Table S5** List of the differentially expressed miRNAs identified in blood leukocytes in PQDS. 19](#_Toc47107815)

[**Table S6** List of the differentially expressed miRNAs in serums in PDHS. 22](#_Toc47107816)

[**Table S7** List of the differentially expressed miRNAs in serum in PQDS 24](#_Toc47107817)

[**Table S8** The detailed interaction relationships among the PDHS-specific genes 27](#_Toc47107818)

[**Table S9** The detailed interaction relationships among the PQDS-specific genes 29](#_Toc47107819)

[**Table S10** The experimental evidences supporting the PDHS-specific miRNA-gene interaction pairs 32](#_Toc47107820)

[**Table S11** The experimental evidences supporting the PQDS-specific miRNA-gene interaction pairs 33](#_Toc47107821)

[**Table S12** The experimentally-supported direct targets of the exosome-contained has-miR-122-5p 34](#_Toc47107822)

[**Additional Figures** 37](#_Toc47107823)

[**Figure S1** Glutamatergic synapse pathway (hsa04724). 37](#_Toc47107824)

[**Figure S2** Serotonergic/Dopaminergic synapse pathway (hsa04726). 38](#_Toc47107825)

[**Figure S3** Dopaminergic synapse pathway (hsa04728). 39](#_Toc47107826)

[**Figure S4** NOD-like receptor signaling pathway (hsa04621). 40](#_Toc47107827)

[**Figure S5** ECM-receptor interaction (hsa04512). 41](#_Toc47107828)

[**Figure S6** Cell adhesion molecules (CAMs) (hsa04514). 42](#_Toc47107829)

[**Figure S7** MAPK signaling pathway (hsa04010). 43](#_Toc47107830)

[**Figure S8** Th1 and Th2 cell differentiation (hsa04658). 44](#_Toc47107831)

[**Figure S9** IL-17 signaling pathway (hsa04657). 45](#_Toc47107832)

[**Figure S10** Th17 cell differentiation pathway (hsa04659). 46](#_Toc47107833)

[**Figure S11** Cytokine-cytokine receptor interaction pathway (hsa04060). 47](#_Toc47107834)

[**Figure S12** Arachidonic acid metabolism (hsa590). 48](#_Toc47107835)

[**Figure S13** The network detailing the interaction relationships of the PDHS-specific genes in leukocytes. 49](#_Toc47107836)

[**Figure S14** The network detailing the interaction relationships of the PQDS-specific genes in leukocytes. 50](#_Toc47107837)

**Supplementary methods**

**Inclusion and exclusion criteria for participants**

(**1**) The modern medical gastroscopy and pathological examination verified the diagnosis of chronic atrophic gastritis (CAG) according to the pathological diagnosis and grading standards “*China Chronic Gastritis Consensus*”, proposed in Shanghai, 2012 (1).

***Endoscopic Diagnosis*** According to endoscopic diagnosis criteria, CAG manifests in mucosal inflammatory changes observed by the naked eye or by special imaging methods, which include alternating red and white mucosa with white predominant, flattened or even disappeared rugae, exposed blood vessels and may be accompanied with granular or nodular mucosa in some cases.

***Pathological Diagnosis*** The fundamental manifestations of pathology are mainly atrophy and intestinal metaplasia. Degree of atrophy is evaluated by how many 1/3 of the appropriate glands is lost; the details are as follows: 0. no loss of the appropriate glands; 1. the loss of the appropriate glands is less than 1/3 of the original; 2. the loss of the appropriate glands is between1/3 and 2/3 of the original; 3. the loss of the appropriate glands is more than 2/3 of the original, in this case few glands are remained, or even completely disappear. And the degree of intestinal metaplasia includes: 0. no intestinal metaplasia; 1. the intestinal metaplasia area accounts for less than 1/3 of the glands and whole superficial epithelium; 2. the intestinal metaplasia area accounts for 1/3 to 2/3 of the glands and whole superficial epithelium; 3. the intestinal metaplasia area accounts for more than 2/3 of the glands and whole superficial epithelium.

No abnormality was found in blood, urine, stool routine, biochemical tests, chest X-ray and electrocardiogram.

(**2**) Two senior TCM practitioners confirmed the CAG patients with the TCM-defined Pi-qi-deficiency syndrome (PQDS) or Pi-wei damp-heat syndrome (PDHS) according to “*Guiding Principle for Clinical Research on New Drugs of Traditional Chinese Medicine”* published in 2002 (2).

***TCM syndrome diagnosis*** According to the consensus on TCM diagnosis and treatment of chronic atrophic gastritis (CAG) (1, 2), the CAG patients with the TCM-defined PDHS or PQDS were selected.

*The diagnosis criteria of PQDS*: primary symptoms include loss of appetite, fatigue, weakness, abdominal distention after eating or noontime, abnormal stool (loose stool, shapeless stool, sometimes loose stool or sometimes dry stool); secondary symptoms include lassitude, and no desire to talk, bland in mouth and absence of thirst, continuous mild abdominal pain, nausea and vomiting, fullness in the stomach, borborygmus, sallow complexion, edema, lack of strength in defecating, pale tongue, enlarged and teeth-printed tongue, thin-white tongue coating and weak pulse. A diagnosis of PQDS should include two of primary symptoms or one of primary symptoms with two of secondary symptoms simultaneously.

*The diagnostic criteria of PDHS*: primary symptoms include fullness and distention of gastric cavity, loss of appetite, loose stool with discomforting evacuation, red tongue with yellow greasy tongue coating; secondary symptoms include fatigued body and limbs, reversal heat, abdominal distension, nausea with vomiting, bright yellowish and soft skin and sclera, and rapid pulse. A diagnosis of PDHS should include three of primary symptoms (tongue and pulse included) or two of primary symptoms (tongue and pulse included) and two of secondary symptoms simultaneously.

**(3)** Exclusion criteria

*Patients would be excluded if they meet one of the following criteria*: (1) Being under 18 years old or over 65 years old; (2) Being accompanied with gastric ulcer, duodenal ulcer, special types of gastritis or gastrointestinal hemorrhage; (3) With histopathological examination showing atrophic changes, dysplasia or suspected malignant changes in gastric mucosa; (4) Not PQDS according to syndrome differentiation in TCM; (5) Varied diagnosis of syndrome differentiation in TCM before treatment; (6) With unclear diagnosis of syndrome differentiation in TCM; (7) With history of gastric surgery; (8) Currently being accompanied with *H. pylori* infection; (9) With serious comorbidities of heart, lung, liver, kidney or blood system (such as cardiac function above grade II, value of alanine aminotransferase (ALT) and/or aspartate aminotransferase (AST) 1.5 times higher than the upper normal limit, creatinine (Cr) higher than the upper normal limit, etc.) or having a life-threatening illness (such as tumor or AIDS); (10) With psychiatric disorders or a history of alcohol or drug abuse; (11) Female patients preparing for a baby, pregnant or lactating.

(**4**) Age, between 18 and 65 years. Subjects voluntarily participated in the research and signed the informed consent.

**The experimental design and route**

Experimental design and route of the study. CAG, chronic atrophic gastritis; PDHS, Pi-wei damp-heat syndrome; PQDS, Pi-qi-deficiency syndrome.


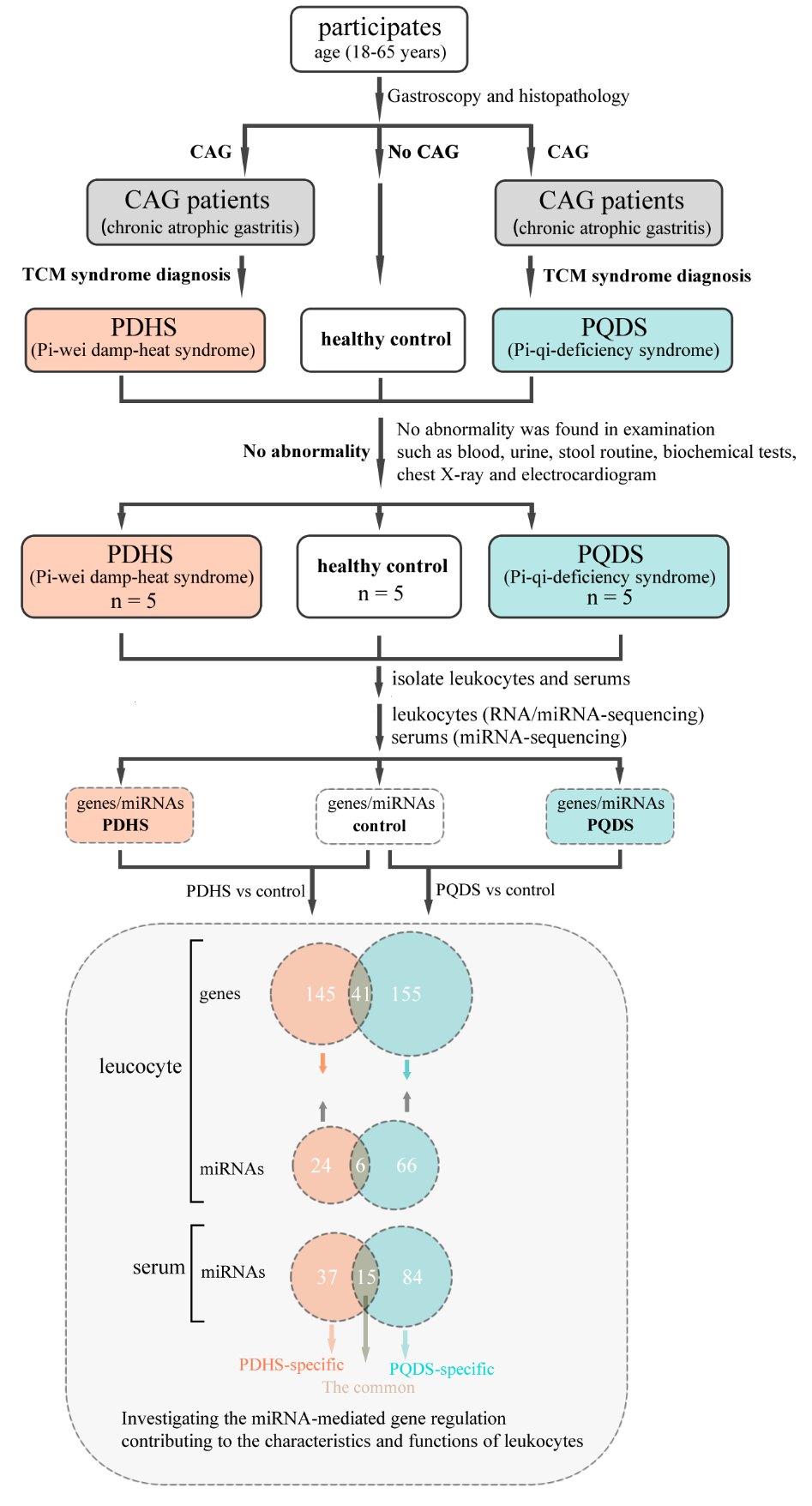


**miRNA sequencing**

miRNA sequencing (miRNA-seq) was performed by OEbiotech company (Shanghai, China). In short, the miRNA sequencing libraries were constructed using the RNAs isolated from the lymphocyte and serum samples according to instructions. High throughput miRNA-seq was performed using the Illumina HiSeq 4000 Sequencer (Illumina; San Diego, CA). The adapter sequences of generated RAW reads were removed using the popular *Cutadapt* software (<https://cutadapt.readthedocs.io/en/stable>), and the obtained sequences were filtered to remove sequences that were less than 15 bp or more than 41 bp in length. The Q20 quality control of the filtered sequences was performed by the *Fastx_Toolkit* (version 0.0.13, <http://hannonlab.cshl.edu/fastx_toolkit>) and NGSQC Toolkit (version 2.3.2) was used to filter out the reads containing N bases (3). The *BLASTN* software was used to compare the clean reads with the Rfam database (version 10.0, <http://rfam.xfam.org>), a web-accessible interface for annotating families of non-coding RNA sequences (4, 5). The results (E-value ≤ 0.01) were extracted, and the annotated mRNA, rRNA, snRNA, snoRNA and tRNA were removed. The extracted sequences were compared with the repeat database to identify and remove the repeated sequences using the *RepeatMasker* web server (<http://www.repeatmasker.org/cgi-bin/WEBRepeatMasker>). Finally, the high quantity clean reads obtained were used for subsequent annotating analysis of the mature miRNAs, and the small RNAs which were not annotated were used for the prediction of new miRNAs using Mirdeep2 (a software package developed for the identification of new and known miRNAs in deep sequencing data) (6).

**RNA sequencing**

RNA sequencing (RNA-seq) was performed by OEbiotech company (Shanghai, China). Briefly, total RNAs of leukocytes were extracted using the RNA isolation Kit (Ambion) following the manufacturer’s instructions, and stored at -80 ℃. RNA integrity Number (RIN) was detected using the popular Agilent 2100 Bioanalyzer. RNA samples (RIN ≥7) were subjected to the subsequent high throughput sequencing analysis. The whole transcriptomic sequencing libraries were constructed using the commercial kit (Illumina, RS-122-2301) termed “TruSeq Stranded Total RNA with Ribo-Zero Globin” (this kit keeps an efficient work flow enabling removal of ribosomal RNA and globin mRNA in a single step). These resultant sequencing libraries were sequenced by the well-known Illumina sequencing platform (HiSeqTM 2500) and 150 bp/125bp paired-end raw reads were generated. The obtained raw reads were filtered and processed for the subsequent annotation and differential expression analyses of lncRNAs and mRNAs.

**Supplemental Tables**

**Table S1** List of leukocytes and serum samples from the clinical participants.

| **Id** | **Sex** | **Age** | **Group** | **Leukocytes ^a^** | |  | **Serum ^b^** | **TCM-based diagnosis** | **Clinical diagnosis** |
| --- | --- | --- | --- | --- | --- | --- | --- | --- | --- |
|  |  |  |  | **RNA-seq ^c^** | **miRNA-seq ^c^** |  | **miRNA-seq ^c^** |  |  |
| A_1 | Male | 26 | Control | SRR10513209 | SRR11483205 | SRR11548322 | | Balanced constitution | Health |
| A_2 | Female | 28 | Control | SRR10513208 | SRR11483204 | SRR11548321 | | Balanced constitution | Health |
| A_3 | Female | 24 | Control | SRR10513204 | SRR11483203 | SRR11548320 | | Balanced constitution | Health |
| A_4 | Male | 25 | Control | SRR10513203 | SRR11483202 | / | | Balanced constitution | Health |
| A_5 | Male | 26 | Control | SRR10513202 | SRR11483201 | / | | Balanced constitution | Health |
| F_1 | Female | 65 | PQDS | SRR11548312 | SRR11548310 | SRR11548329 | | Pi-qi deficiency syndrome | CAG |
| F_2 | Male | 65 | PQDS | SRR11548311 | SRR11548339 | SRR11548328 | | Pi-qi deficiency syndrome | CAG |
| F_3 | Female | 30 | PQDS | SRR11548330 | SRR11548338 | SRR11548327 | | Pi-qi deficiency syndrome | CAG |
| F_4 | Male | 49 | PQDS | SRR11548319 | SRR11548337 | SRR11548326 | | Pi-qi deficiency syndrome | CAG |
| F_7 | Male | 56 | PQDS | SRR11548318 | SRR11548336 | / | | Pi-qi deficiency syndrome | CAG |
| G_1 | Male | 64 | PDHS | SRR11548317 | SRR11548335 | SRR11548325 | | Pi-wei damp-heat syndrome | CAG |
| G_2 | Male | 49 | PDHS | SRR11548316 | SRR11548334 | SRR11548324 | | Pi-wei damp-heat syndrome | CAG |
| G_3 | Male | 60 | PDHS | SRR11548315 | SRR11548333 | SRR11548323 | | Pi-wei damp-heat syndrome | CAG |
| G_4 | Female | 61 | PDHS | SRR11548314 | SRR11548332 | / | | Pi-wei damp-heat syndrome | CAG |
| G_5 | Male | 65 | PDHS | SRR11548313 | SRR11548331 | / | | Pi-wei damp-heat syndrome | CAG |

**Notes**: TCM, traditional Chinese medicine; CAG, chronic atrophic gastritis; PQDS, Pi-qi deficiency syndrome; PDHS, Pi-wei damp-heat syndrome.

^a^ The accession numbers for the leukocyte RNA-seq and miRNA-seq raw data deposited in the NCBI Sequence Read Archive (SRA).

^b^ The accession numbers for the serum miRNA-seq raw data deposited in the NCBI Sequence Read Archive (SRA).

^c^ The link for the corresponding NCBI bioProject: <https://www.ncbi.nlm.nih.gov/bioproject/PRJNA591186>.

**Table S2** List of the differentially expressed genes identified in blood leukocytes in PDHS.

| **gene_id ^a^** | **baseMean_control_A ^b^** | **baseMean_case_G ^b^** | **foldChange ^c^**  **G(n=5)/A(n=5)** | **log2FoldChange** | ***P*-value** | **padj ^d^** | **up_down** |
| --- | --- | --- | --- | --- | --- | --- | --- |
| ABCA13 | 275.716 | 646.2977 | 2.344071 | 1.229016 | 0.012265 | 1 | Up |
| ACOT4 | 16.55354 | 37.86469 | 2.287407 | 1.193713 | 0.012123 | 1 | Up |
| ACSBG1 | 9.077615 | 30.62451 | 3.37363 | 1.754302 | 0.046573 | 1 | Up |
| ADAM23 | 54.67518 | 224.1983 | 4.10055 | 2.035817 | 0.044643 | 1 | Up |
| ADAMTSL5 | 8.970107 | 27.18937 | 3.031109 | 1.599846 | 0.006693 | 0.872731 | Up |
| ADGRA3 | 105.0588 | 46.4162 | 0.441812 | -1.1785 | 0.00971 | 1 | Down |
| AK5 | 412.0303 | 153.5605 | 0.372692 | -1.42394 | 0.031951 | 1 | Down |
| AKAP3 | 7.599507 | 18.92414 | 2.49018 | 1.31625 | 0.048206 | 1 | Up |
| ALPK2 | 18.3133 | 79.54459 | 4.343541 | 2.118872 | 0.023236 | 1 | Up |
| APOBEC3H | 29.96517 | 61.29014 | 2.045379 | 1.032368 | 0.008802 | 1 | Up |
| AQP1 | 2.809829 | 12.63861 | 4.498 | 2.169284 | 0.026631 | 1 | Up |
| ARG1 | 142.2308 | 344.252 | 2.420375 | 1.275231 | 0.013954 | 1 | Up |
| ARHGEF4 | 42.2774 | 17.87232 | 0.422739 | -1.24216 | 0.009059 | 1 | Down |
| AZU1 | 25.2988 | 71.46713 | 2.824922 | 1.498211 | 0.018999 | 1 | Up |
| BAAT | 9.51777 | 2.159931 | 0.226937 | -2.13964 | 0.042008 | 1 | Down |
| BEGAIN | 5.683745 | 44.37676 | 7.807663 | 2.964891 | 0.00057 | 0.257583 | Up |
| BFSP1 | 32.95152 | 66.81122 | 2.027561 | 1.019745 | 0.036337 | 1 | Up |
| BHLHA15 | 8.723618 | 0.98284 | 0.112664 | -3.1499 | 0.011404 | 1 | Down |
| BPI | 92.95817 | 272.791 | 2.934556 | 1.553142 | 0.025647 | 1 | Up |
| BRSK1 | 36.4962 | 17.51782 | 0.47999 | -1.05892 | 0.029322 | 1 | Down |
| C19orf33 | 8.463068 | 25.95278 | 3.066592 | 1.616636 | 0.008897 | 1 | Up |
| C4orf48 | 12.67652 | 32.03176 | 2.526857 | 1.337344 | 0.028758 | 1 | Up |
| C4orf50 | 79.37741 | 233.4437 | 2.940933 | 1.556274 | 0.000116 | 0.198247 | Up |
| CACHD1 | 93.02277 | 31.93452 | 0.343298 | -1.54247 | 0.000822 | 0.295156 | Down |
| CAMP | 134.5573 | 347.4161 | 2.581918 | 1.368443 | 0.004761 | 0.782052 | Up |
| CASP5 | 1036.441 | 504.1428 | 0.486418 | -1.03973 | 0.022395 | 1 | Down |
| CCL3 | 67.884 | 160.1708 | 2.359478 | 1.238468 | 0.00704 | 0.904212 | Up |
| CCL4L2 | 175.8133 | 584.8203 | 3.326371 | 1.733949 | 0.031358 | 1 | Up |
| CD248 | 41.74714 | 4.471088 | 0.107099 | -3.22298 | 2.68E-08 | 0.000458 | Down |
| CEACAM6 | 41.49064 | 101.6223 | 2.449283 | 1.292359 | 0.030967 | 1 | Up |
| CEACAM8 | 102.5896 | 265.2344 | 2.585393 | 1.370383 | 0.02714 | 1 | Up |
| CFAP97D2 | 65.04992 | 30.90856 | 0.475151 | -1.07354 | 0.011592 | 1 | Down |
| CHN1 | 26.11023 | 54.65272 | 2.093153 | 1.065678 | 0.009005 | 1 | Up |
| CHST13 | 35.67079 | 78.31901 | 2.195606 | 1.134619 | 0.020095 | 1 | Up |
| CNTNAP2 | 107.6092 | 46.61549 | 0.433192 | -1.20692 | 0.010993 | 1 | Down |
| COL27A1 | 0 | 4.394717 | Inf | Inf | 0.014694 | 1 | Up |
| CORO2B | 22.7384 | 5.1588 | 0.226876 | -2.14002 | 0.001337 | 0.380641 | Down |
| CR2 | 289.0588 | 117.9221 | 0.407952 | -1.29353 | 1.15E-06 | 0.009843 | Down |
| CRYBG2 | 20.27435 | 41.11603 | 2.027983 | 1.020046 | 0.039765 | 1 | Up |
| CTSG | 10.34176 | 49.59173 | 4.795289 | 2.261618 | 0.014309 | 1 | Up |
| CXCL5 | 158.2314 | 373.6737 | 2.361565 | 1.239743 | 0.003972 | 0.743624 | Up |
| CYP27A1 | 533.4147 | 1246.379 | 2.336604 | 1.224413 | 0.001029 | 0.325467 | Up |
| CYP4F12 | 163.3724 | 351.6951 | 2.15272 | 1.106161 | 0.021575 | 1 | Up |
| DAB2IP | 32.80124 | 9.413901 | 0.286998 | -1.80089 | 0.002816 | 0.646625 | Down |
| DCANP1 | 66.02467 | 23.42978 | 0.354864 | -1.49466 | 0.000137 | 0.198247 | Down |
| DEFA1 | 10.56105 | 40.52313 | 3.837035 | 1.939992 | 0.011988 | 1 | Up |
| DEFA1B | 2.110959 | 12.13152 | 5.746922 | 2.522789 | 0.012443 | 1 | Up |
| DEFA3 | 162.4123 | 589.7444 | 3.631156 | 1.860429 | 0.006417 | 0.856396 | Up |
| DNAJC6 | 63.92629 | 130.3459 | 2.039003 | 1.027864 | 0.003122 | 0.666608 | Up |
| EDA | 76.95072 | 187.5652 | 2.437472 | 1.285386 | 2.01E-06 | 0.011461 | Up |
| EGR1 | 73.68869 | 272.3079 | 3.695382 | 1.885723 | 0.030478 | 1 | Up |
| EGR3 | 77.837 | 463.1177 | 5.949839 | 2.572851 | 0.017306 | 1 | Up |
| EMILIN1 | 12.2452 | 29.66519 | 2.422598 | 1.276555 | 0.02005 | 1 | Up |
| EML6 | 256.5916 | 120.9826 | 0.471499 | -1.08467 | 0.000252 | 0.198247 | Down |
| EPHX3 | 4.045448 | 15.17714 | 3.751659 | 1.907529 | 0.018374 | 1 | Up |
| ESPN | 9.027588 | 25.88601 | 2.867433 | 1.51976 | 0.018814 | 1 | Up |
| F2RL3 | 15.5054 | 35.81255 | 2.309682 | 1.207694 | 0.02666 | 1 | Up |
| FAM229A | 28.19566 | 64.8768 | 2.30095 | 1.202229 | 0.046644 | 1 | Up |
| FBLN2 | 62.43507 | 13.71273 | 0.219632 | -2.18684 | 0.000303 | 0.19894 | Down |
| FCRL5 | 452.9548 | 157.6077 | 0.347955 | -1.52303 | 0.02591 | 1 | Down |
| FGF2 | 9.485178 | 34.59379 | 3.647142 | 1.866767 | 0.004172 | 0.750164 | Up |
| FIGN | 1.19341 | 19.24091 | 16.12263 | 4.011015 | 9.97E-06 | 0.034063 | Up |
| FLT4 | 157.7723 | 68.15327 | 0.431972 | -1.21099 | 0.00528 | 0.804233 | Down |
| FOSB | 165.1327 | 396.2914 | 2.399835 | 1.262936 | 0.001522 | 0.419288 | Up |
| FOXJ1 | 15.82554 | 3.463902 | 0.21888 | -2.19179 | 0.018624 | 1 | Down |
| FSTL1 | 127.6534 | 386.8588 | 3.030541 | 1.599575 | 0.045972 | 1 | Up |
| FXYD1 | 1.704634 | 8.82484 | 5.176971 | 2.372108 | 0.04311 | 1 | Up |
| GATA6 | 3.619265 | 14.49113 | 4.003888 | 2.001402 | 0.039444 | 1 | Up |
| GATM | 50.77706 | 22.28351 | 0.43885 | -1.1882 | 0.007938 | 0.968609 | Down |
| GJB6 | 2.242715 | 11.24459 | 5.013831 | 2.325913 | 0.033423 | 1 | Up |
| GNAI1 | 42.72393 | 14.10747 | 0.330201 | -1.59859 | 0.001272 | 0.368318 | Down |
| GNAO1 | 68.42428 | 150.4375 | 2.198598 | 1.136584 | 0.021525 | 1 | Up |
| GNG10 | 13.5499 | 30.73436 | 2.268235 | 1.18157 | 0.022513 | 1 | Up |
| GOLGA6L10 | 46.47304 | 22.7784 | 0.490142 | -1.02873 | 0.015487 | 1 | Down |
| GPAT2 | 44.9451 | 9.461306 | 0.210508 | -2.24805 | 0.000201 | 0.198247 | Down |
| GPM6A | 20.09366 | 4.500827 | 0.223992 | -2.15848 | 0.00297 | 0.653354 | Down |
| GPR20 | 1.98701 | 10.5823 | 5.325743 | 2.412983 | 0.017903 | 1 | Up |
| HEY1 | 44.81386 | 102.2956 | 2.282678 | 1.190727 | 0.002989 | 0.653354 | Up |
| HIC1 | 28.6039 | 100.374 | 3.509102 | 1.811102 | 0.005848 | 0.832526 | Up |
| IFI27 | 13.1291 | 2.297132 | 0.174965 | -2.51486 | 0.026967 | 1 | Down |
| IFNG | 54.96152 | 119.2121 | 2.16901 | 1.117037 | 0.000198 | 0.198247 | Up |
| IGFBP2 | 3.319228 | 11.96254 | 3.604013 | 1.849604 | 0.046004 | 1 | Up |
| IGFBP3 | 55.48547 | 132.2581 | 2.383652 | 1.253174 | 0.000285 | 0.19894 | Up |
| IGLL5 | 760.265 | 373.3189 | 0.491038 | -1.02609 | 0.048287 | 1 | Down |
| IL23R | 86.9386 | 25.55117 | 0.293899 | -1.76661 | 0.004085 | 0.743624 | Down |
| INHBB | 10.34491 | 23.5857 | 2.279933 | 1.188992 | 0.046794 | 1 | Up |
| IQCA1 | 11.92147 | 0.837494 | 0.070251 | -3.83134 | 0.000698 | 0.265128 | Down |
| ISM1 | 16.75151 | 37.31688 | 2.227672 | 1.155537 | 0.016573 | 1 | Up |
| JAKMIP1 | 307.0487 | 615.9576 | 2.006059 | 1.004364 | 0.004025 | 0.743624 | Up |
| KCNMB4 | 117.3995 | 58.36471 | 0.497146 | -1.00826 | 0.016468 | 1 | Down |
| KIAA1217 | 10.60884 | 1.210195 | 0.114074 | -3.13196 | 0.00489 | 0.789235 | Down |
| KLHL14 | 235.5996 | 106.4499 | 0.451826 | -1.14616 | 4.21E-05 | 0.103088 | Down |
| LDB2 | 34.82531 | 12.64375 | 0.363062 | -1.46171 | 0.031291 | 1 | Down |
| LGALS9B | 65.24479 | 26.22771 | 0.401989 | -1.31477 | 0.032812 | 1 | Down |
| LOC100996693 | 0 | 3.528654 | Inf | Inf | 0.036652 | 1 | Up |
| LOC101927245 | 0.997275 | 7.544291 | 7.564909 | 2.919323 | 0.024839 | 1 | Up |
| LOC102724843 | 17.54949 | 6.157709 | 0.350877 | -1.51096 | 0.041873 | 1 | Down |
| LOC107986351 | 225.5466 | 91.78467 | 0.406943 | -1.2971 | 0.01317 | 1 | Down |
| LOC644090 | 12.40041 | 3.034964 | 0.244747 | -2.03064 | 0.028701 | 1 | Down |
| LOC644249 | 0.200481 | 4.753246 | 23.70921 | 4.567376 | 0.03488 | 1 | Up |
| LOC646652 | 28.35157 | 2.979687 | 0.105098 | -3.2502 | 0.013951 | 1 | Down |
| LRFN2 | 76.13787 | 185.9778 | 2.442645 | 1.288444 | 0.049069 | 1 | Up |
| LRRN3 | 555.2191 | 263.6136 | 0.474792 | -1.07463 | 0.026519 | 1 | Down |
| LTK | 425.3373 | 207.618 | 0.488126 | -1.03468 | 0.019422 | 1 | Down |
| MATN2 | 27.88452 | 12.48531 | 0.447751 | -1.15923 | 0.036245 | 1 | Down |
| MET | 5.524399 | 0.610253 | 0.110465 | -3.17834 | 0.046895 | 1 | Down |
| METTL24 | 49.96592 | 22.32735 | 0.446852 | -1.16213 | 0.038963 | 1 | Down |
| MLF1 | 10.07457 | 28.18329 | 2.797469 | 1.484122 | 0.036487 | 1 | Up |
| MPO | 94.78693 | 212.6677 | 2.243639 | 1.165841 | 0.01605 | 1 | Up |
| MPPED2 | 24.15779 | 7.091409 | 0.293545 | -1.76834 | 0.039712 | 1 | Down |
| MTCL1 | 39.12838 | 17.68234 | 0.451906 | -1.14591 | 0.046791 | 1 | Down |
| MXRA8 | 23.24433 | 5.460993 | 0.234939 | -2.08964 | 0.005309 | 0.804233 | Down |
| MYL4 | 10.81431 | 33.83494 | 3.12872 | 1.645572 | 0.002628 | 0.639516 | Up |
| MYO18B | 16.57143 | 5.080121 | 0.306559 | -1.70576 | 0.023848 | 1 | Down |
| N4BP3 | 136.9251 | 50.91185 | 0.371823 | -1.42731 | 0.000248 | 0.198247 | Down |
| NBEA | 538.2538 | 227.2318 | 0.422165 | -1.24412 | 8.21E-06 | 0.034063 | Down |
| NEFL | 14.45437 | 77.37703 | 5.353194 | 2.4204 | 0.034018 | 1 | Up |
| NETO1 | 62.90031 | 30.67074 | 0.487609 | -1.0362 | 0.00817 | 0.983725 | Down |
| NLGN4Y | 18.73371 | 53.93648 | 2.879114 | 1.525625 | 0.023525 | 1 | Up |
| NLRP14 | 3.682088 | 0 | 0 | -Inf | 0.034396 | 1 | Down |
| NR4A2 | 116.0311 | 239.5754 | 2.064751 | 1.045968 | 0.000898 | 0.306813 | Up |
| NRCAM | 229.8042 | 41.23486 | 0.179435 | -2.47847 | 0.000335 | 0.204312 | Down |
| OCA2 | 15.74357 | 1.532048 | 0.097313 | -3.36123 | 0.000485 | 0.243793 | Down |
| OLFM4 | 68.09493 | 215.7902 | 3.168962 | 1.66401 | 0.045236 | 1 | Up |
| OLR1 | 14.04735 | 41.69609 | 2.968253 | 1.569614 | 0.004351 | 0.768398 | Up |
| ORM1 | 102.319 | 235.0838 | 2.297557 | 1.200101 | 0.035007 | 1 | Up |
| OSM | 287.8392 | 626.6467 | 2.177072 | 1.122389 | 0.025625 | 1 | Up |
| PARD6G | 3.474008 | 13.62206 | 3.921136 | 1.971272 | 0.03587 | 1 | Up |
| PLGLB2 | 76.03641 | 214.1402 | 2.816285 | 1.493793 | 0.00017 | 0.198247 | Up |
| PMEPA1 | 70.37189 | 28.52287 | 0.405316 | -1.30288 | 0.000656 | 0.265128 | Down |
| PRR29 | 29.08142 | 12.45092 | 0.42814 | -1.22385 | 0.028343 | 1 | Down |
| PTPRF | 16.81995 | 36.38605 | 2.163268 | 1.113212 | 0.02176 | 1 | Up |
| PTPRK | 212.2907 | 97.62671 | 0.459873 | -1.12069 | 0.003234 | 0.667057 | Down |
| RAB5IF | 89.1319 | 239.0271 | 2.681723 | 1.42316 | 0.031348 | 1 | Up |
| RBMS3 | 35.18804 | 12.1191 | 0.34441 | -1.5378 | 0.004452 | 0.772868 | Down |
| RCAN2 | 14.87675 | 49.93907 | 3.356854 | 1.74711 | 0.030922 | 1 | Up |
| RDH14 | 6.225904 | 23.65259 | 3.799061 | 1.925643 | 0.04467 | 1 | Up |
| REG4 | 9.638806 | 1.154808 | 0.119808 | -3.0612 | 0.009656 | 1 | Down |
| RHD | 21.12457 | 45.19095 | 2.13926 | 1.097112 | 0.03313 | 1 | Up |
| RMI2 | 38.84966 | 17.15668 | 0.441617 | -1.17913 | 0.009125 | 1 | Down |
| RNF17 | 0.200481 | 80.7261 | 402.6621 | 8.653426 | 0.041955 | 1 | Up |
| ROBO3 | 75.26366 | 29.99104 | 0.39848 | -1.32742 | 0.000267 | 0.198247 | Down |
| RPH3A | 47.1122 | 102.2031 | 2.169356 | 1.117267 | 0.039937 | 1 | Up |
| SCN3A | 165.0429 | 65.90029 | 0.399292 | -1.32448 | 0.002818 | 0.646625 | Down |
| SCN8A | 27.09238 | 61.88524 | 2.284231 | 1.191708 | 0.032627 | 1 | Up |
| SHANK1 | 8.687087 | 1.207542 | 0.139004 | -2.8468 | 0.022959 | 1 | Down |
| SHISA2 | 8.442514 | 1.340542 | 0.158785 | -2.65486 | 0.035748 | 1 | Down |
| SLC16A10 | 187.5747 | 82.3382 | 0.438962 | -1.18783 | 0.035763 | 1 | Down |
| SLC18A1 | 9.951132 | 1.44115 | 0.144823 | -2.78764 | 0.011626 | 1 | Down |
| SLC24A2 | 5.172633 | 0.383012 | 0.074046 | -3.75544 | 0.03936 | 1 | Down |
| SLC4A10 | 1120.786 | 292.7928 | 0.261239 | -1.93656 | 0.000165 | 0.198247 | Down |
| SLX1B | 12.48291 | 157.3108 | 12.60209 | 3.655591 | 0.003426 | 0.696738 | Up |
| SMARCA1 | 19.3566 | 4.019747 | 0.207668 | -2.26765 | 0.002664 | 0.639516 | Down |
| SORCS3 | 19.88047 | 66.24208 | 3.332017 | 1.736396 | 0.032685 | 1 | Up |
| SPTSSB | 63.88324 | 27.04645 | 0.423373 | -1.24 | 0.011645 | 1 | Down |
| STYK1 | 41.47957 | 91.29897 | 2.201059 | 1.138198 | 0.028059 | 1 | Up |
| TARM1 | 1.984448 | 16.06028 | 8.09307 | 3.016687 | 0.047219 | 1 | Up |
| TEAD3 | 7.699547 | 1.365852 | 0.177394 | -2.49497 | 0.046716 | 1 | Down |
| TENM4 | 14.98534 | 55.21458 | 3.684574 | 1.881498 | 0.027599 | 1 | Up |
| THBS1 | 2253.689 | 4575.172 | 2.030081 | 1.021537 | 0.015578 | 1 | Up |
| TIMD4 | 45.65206 | 21.18162 | 0.46398 | -1.10787 | 0.013524 | 1 | Down |
| TMEM160 | 28.58304 | 86.47845 | 3.025516 | 1.597181 | 0.043269 | 1 | Up |
| TMEM178B | 3.042855 | 16.20145 | 5.324423 | 2.412625 | 0.009009 | 1 | Up |
| TMEM220 | 59.05886 | 27.6077 | 0.467461 | -1.09708 | 0.004944 | 0.789235 | Down |
| TMEM255A | 20.33276 | 49.2914 | 2.424236 | 1.27753 | 0.01031 | 1 | Up |
| TMIGD2 | 152.1956 | 73.71303 | 0.484331 | -1.04594 | 0.000829 | 0.295156 | Down |
| TNFRSF17 | 62.19309 | 25.19967 | 0.405184 | -1.30335 | 0.037064 | 1 | Down |
| TNIP3 | 39.1221 | 103.2395 | 2.638906 | 1.39994 | 0.005388 | 0.804233 | Up |
| TRIB1 | 1777.797 | 3569.842 | 2.008015 | 1.00577 | 0.033312 | 1 | Up |
| TRIM2 | 64.0956 | 129.0261 | 2.013025 | 1.009365 | 0.000692 | 0.265128 | Up |
| TTC24 | 162.0303 | 78.24812 | 0.482923 | -1.05014 | 0.000435 | 0.233568 | Down |
| TTC28 | 166.0421 | 82.73717 | 0.49829 | -1.00494 | 0.000191 | 0.198247 | Down |
| TXNRD3 | 38.84632 | 17.54653 | 0.451691 | -1.14659 | 0.018863 | 1 | Down |
| ULBP3 | 10.72663 | 25.9225 | 2.416649 | 1.273008 | 0.027222 | 1 | Up |
| VAMP7 | 121.2683 | 10.81914 | 0.089217 | -3.48654 | 0.017713 | 1 | Down |
| VSTM4 | 117.7477 | 18.4395 | 0.156602 | -2.67483 | 0.013271 | 1 | Down |
| VWDE | 15.5141 | 45.05351 | 2.904036 | 1.538059 | 0.00094 | 0.311005 | Up |
| WASF3 | 48.59079 | 113.2279 | 2.330234 | 1.220475 | 0.044064 | 1 | Up |
| XKRX | 102.421 | 35.60868 | 0.34767 | -1.52421 | 0.000545 | 0.257583 | Down |
| ZNF285 | 57.4492 | 27.04588 | 0.470779 | -1.08688 | 0.019598 | 1 | Down |
| ZNF404 | 69.92717 | 32.58873 | 0.466038 | -1.10148 | 0.003672 | 0.709965 | Down |
| ZNF415 | 100.7853 | 43.92059 | 0.435784 | -1.19832 | 0.001602 | 0.434445 | Down |
| ZNF610 | 31.62954 | 12.87262 | 0.406981 | -1.29697 | 0.013396 | 1 | Down |
| ZNF660 | 69.79472 | 34.37155 | 0.492466 | -1.0219 | 0.038776 | 1 | Down |
| ZNF662 | 83.25893 | 33.1456 | 0.398103 | -1.32879 | 0.000724 | 0.26896 | Down |
| ZNF704 | 21.71608 | 3.170716 | 0.146008 | -2.77588 | 0.000265 | 0.198247 | Down |

**Note:** TCM, traditional Chinese medicine; CAG, chronic atrophic gastritis; PDHS: Pi-wei damp-heat syndrome; PQDS: Pi-qi-deficiency syndrome.

^a^ The genes marked in brown font denoted the common differential genes observed both in PDHS and PQDS, compared with the healthy control.

^b^ normalized mean count value;

^c^ normalized mean count value (CAG with TCM-defined PDHS, n=5) / normalized mean count value (healthy control, n=5);

^d^ the adjusted *P*-value.

**Table S3** List of the differentially expressed genes identified in blood leukocytes in PQDS.

| **gene_id ^a^** | **baseMean_control_A** ^b^ | **baseMean_case_F** ^b^ | **foldChange ^c^**  **F(n=5)/A(n=5)** | **log2FoldChange** | ***P*-value** | **padj ^d^** | **up_down** |
| --- | --- | --- | --- | --- | --- | --- | --- |
| A1BG | 25.37172 | 52.10125 | 2.053517 | 1.038097 | 0.017124 | 0.709883 | Up |
| AATK | 1633.299 | 3571.874 | 2.186908 | 1.128893 | 3.60E-08 | 0.000619 | Up |
| ABCG2 | 46.86124 | 22.75381 | 0.485557 | -1.04229 | 0.047296 | 0.929754 | Down |
| ACCSL | 4.012176 | 15.0441 | 3.749612 | 1.906741 | 0.035186 | 0.85594 | Up |
| ACSBG1 | 9.000092 | 38.64806 | 4.294185 | 2.102384 | 0.000201 | 0.108294 | Up |
| ADGRA3 | 104.2085 | 50.71002 | 0.486621 | -1.03913 | 0.013115 | 0.680128 | Down |
| AKAP17A | 23.46549 | 59.69953 | 2.544141 | 1.347179 | 0.037071 | 0.862169 | Up |
| AKAP6 | 131.6842 | 62.12601 | 0.47178 | -1.08381 | 0.00251 | 0.363464 | Down |
| AKR1C1 | 13.53736 | 30.25495 | 2.234922 | 1.160224 | 0.033645 | 0.849057 | Up |
| ALK | 0 | 3.902276 | Inf | Inf | 0.038911 | 0.875496 | Up |
| ANKRD20A4 | 48.19412 | 15.31402 | 0.317757 | -1.654 | 0.015606 | 0.690816 | Down |
| ANO2 | 8.104625 | 24.5269 | 3.026285 | 1.597548 | 0.017926 | 0.716184 | Up |
| AREG | 15.8172 | 75.15952 | 4.751759 | 2.248462 | 0.003667 | 0.414982 | Up |
| ARMCX1 | 33.30823 | 14.83782 | 0.44547 | -1.1666 | 0.028421 | 0.811331 | Down |
| ARSJ | 15.71596 | 2.031386 | 0.129256 | -2.95169 | 0.006823 | 0.549023 | Down |
| ATG2A | 2511.648 | 5034.223 | 2.00435 | 1.003135 | 7.28E-05 | 0.061265 | Up |
| B4GALNT3 | 17.84713 | 57.72345 | 3.234328 | 1.693466 | 0.046475 | 0.929754 | Up |
| BAIAP3 | 229.8096 | 517.6476 | 2.252507 | 1.171531 | 0.00071 | 0.200482 | Up |
| BTNL9 | 36.94227 | 11.6332 | 0.314902 | -1.66703 | 0.022131 | 0.751439 | Down |
| C2orf27A | 141.8485 | 60.30734 | 0.425153 | -1.23394 | 5.39E-05 | 0.054569 | Down |
| C4orf48 | 12.58154 | 34.08901 | 2.709447 | 1.437998 | 0.011644 | 0.665487 | Up |
| CACNB2 | 0.198422 | 8.114647 | 40.89596 | 5.353886 | 0.003117 | 0.388943 | Up |
| CALD1 | 88.07739 | 218.3764 | 2.47937 | 1.309974 | 0.000199 | 0.108294 | Up |
| CAPN5 | 88.51645 | 41.6758 | 0.470825 | -1.08674 | 0.00159 | 0.310205 | Down |
| CAVIN2 | 4040.977 | 8877.67 | 2.196912 | 1.135477 | 0.013246 | 0.680128 | Up |
| CBS | 25.28159 | 9.603072 | 0.379845 | -1.39652 | 0.032361 | 0.836077 | Down |
| CCDC194 | 16.31057 | 36.21433 | 2.220299 | 1.150754 | 0.036019 | 0.862169 | Up |
| CCL23 | 2.227648 | 18.38572 | 8.253422 | 3.044992 | 0.012822 | 0.680128 | Up |
| CCL3 | 67.3418 | 180.1074 | 2.674526 | 1.419283 | 0.027923 | 0.806381 | Up |
| CCL4L2 | 174.3991 | 377.6033 | 2.165168 | 1.114479 | 0.006093 | 0.524643 | Up |
| CD300H | 406.9959 | 71.6802 | 0.17612 | -2.50537 | 4.34E-06 | 0.007474 | Down |
| CD300LD | 54.91486 | 11.78826 | 0.214664 | -2.21985 | 0.001161 | 0.2739 | Down |
| CD99 | 504.0727 | 129.3213 | 0.256553 | -1.96267 | 0.037153 | 0.862169 | Down |
| CFAP161 | 35.76517 | 103.4709 | 2.893063 | 1.532598 | 0.003785 | 0.414982 | Up |
| CFAP97D2 | 64.56219 | 20.09575 | 0.311262 | -1.6838 | 0.000563 | 0.173213 | Down |
| CHADL | 12.17638 | 27.42376 | 2.25221 | 1.171341 | 0.045164 | 0.921469 | Up |
| CHRNA2 | 4.735083 | 18.72029 | 3.953529 | 1.983141 | 0.014434 | 0.684685 | Up |
| CLBA1 | 11.51603 | 30.94983 | 2.687544 | 1.426288 | 0.01956 | 0.730506 | Up |
| CLEC4F | 117.0757 | 35.41912 | 0.302532 | -1.72484 | 0.036354 | 0.862169 | Down |
| CLVS1 | 31.01364 | 10.34775 | 0.333652 | -1.58359 | 0.006737 | 0.544646 | Down |
| CNTD2 | 4.193884 | 15.65895 | 3.733758 | 1.900629 | 0.029422 | 0.819065 | Up |
| CNTNAP2 | 106.7539 | 35.31488 | 0.330806 | -1.59594 | 0.00031 | 0.133364 | Down |
| COL10A1 | 3.010075 | 15.96619 | 5.304251 | 2.407149 | 0.007431 | 0.553282 | Up |
| COL13A1 | 81.75623 | 19.8154 | 0.242372 | -2.04471 | 0.048178 | 0.929754 | Down |
| COL26A1 | 0 | 6.039845 | Inf | Inf | 0.013409 | 0.680128 | Up |
| COL27A1 | 0 | 4.722369 | Inf | Inf | 0.028086 | 0.807358 | Up |
| COL4A2 | 4.140912 | 17.81921 | 4.303208 | 2.105413 | 0.014391 | 0.684503 | Up |
| COL4A4 | 192.7129 | 67.29386 | 0.349192 | -1.51791 | 2.01E-07 | 0.000863 | Down |
| COL5A3 | 212.7356 | 55.2891 | 0.259896 | -1.94399 | 0.032937 | 0.840204 | Down |
| CPXM1 | 14.1594 | 2.065782 | 0.145895 | -2.777 | 0.018507 | 0.723979 | Down |
| CSF2RA | 237.3582 | 105.1637 | 0.443059 | -1.17443 | 0.00588 | 0.51849 | Down |
| CXCL5 | 156.9502 | 349.7556 | 2.228449 | 1.15604 | 0.038952 | 0.875496 | Up |
| CXCL6 | 26.08976 | 56.06496 | 2.148926 | 1.103616 | 0.011115 | 0.653231 | Up |
| CYP2E1 | 36.41824 | 9.592364 | 0.263394 | -1.9247 | 0.005427 | 0.497021 | Down |
| DAB2IP | 32.56448 | 5.613655 | 0.172386 | -2.53629 | 9.80E-05 | 0.070321 | Down |
| DCSTAMP | 0.633084 | 8.021371 | 12.6703 | 3.663379 | 0.014633 | 0.685801 | Up |
| DDTL | 72.12243 | 30.09388 | 0.417261 | -1.26098 | 0.039652 | 0.879662 | Down |
| DNAJB5 | 72.8186 | 163.0684 | 2.239378 | 1.163098 | 0.003832 | 0.414982 | Up |
| EDA | 76.39647 | 195.0268 | 2.552826 | 1.352095 | 1.49E-05 | 0.02339 | Up |
| EGF | 198.3878 | 417.1668 | 2.102785 | 1.072301 | 0.002476 | 0.363464 | Up |
| EGR1 | 73.17035 | 482.9303 | 6.600082 | 2.722484 | 0.003872 | 0.416713 | Up |
| EGR3 | 77.30966 | 335.1382 | 4.335011 | 2.116036 | 0.024196 | 0.772925 | Up |
| EPS8 | 102.8874 | 44.52365 | 0.432742 | -1.20842 | 0.000233 | 0.117894 | Down |
| F2RL3 | 15.38295 | 38.97034 | 2.533346 | 1.341044 | 0.00891 | 0.592383 | Up |
| FGF2 | 9.410159 | 34.04051 | 3.617421 | 1.854961 | 0.001227 | 0.281304 | Up |
| FHL2 | 12.04384 | 27.47898 | 2.28158 | 1.190033 | 0.040913 | 0.888633 | Up |
| FIGN | 1.183809 | 11.0603 | 9.34297 | 3.223881 | 0.012708 | 0.680128 | Up |
| FKBP1B | 23.80123 | 48.44174 | 2.035262 | 1.025214 | 0.017657 | 0.709883 | Up |
| FLT4 | 156.5636 | 69.13928 | 0.441605 | -1.17917 | 0.006176 | 0.526451 | Down |
| FOS | 8237.116 | 26497.5 | 3.216842 | 1.685645 | 0.002838 | 0.383337 | Up |
| FOSB | 163.8777 | 848.5717 | 5.17808 | 2.372417 | 0.016738 | 0.706407 | Up |
| FOXD4L4 | 7.058487 | 0 | 0 | -Inf | 0.00249 | 0.363464 | Down |
| FSTL1 | 126.6474 | 349.3877 | 2.758743 | 1.464011 | 0.00372 | 0.414982 | Up |
| G0S2 | 140.5156 | 983.1955 | 6.997057 | 2.806748 | 0.002123 | 0.341976 | Up |
| GADD45G | 20.89376 | 46.82993 | 2.241336 | 1.164359 | 0.011603 | 0.665487 | Up |
| GJC2 | 10.63759 | 26.33951 | 2.476078 | 1.308057 | 0.035032 | 0.85594 | Up |
| GLIPR1L2 | 22.95497 | 7.831017 | 0.341147 | -1.55153 | 0.034283 | 0.849316 | Down |
| GP1BB | 266.4784 | 571.4878 | 2.144594 | 1.100704 | 0.030488 | 0.819614 | Up |
| GPC1 | 4.070195 | 14.92834 | 3.667721 | 1.874884 | 0.038399 | 0.875496 | Up |
| GPR157 | 61.10546 | 122.9077 | 2.011403 | 1.008202 | 0.001934 | 0.326416 | Up |
| GRHL1 | 54.06247 | 123.9844 | 2.293354 | 1.197459 | 0.012149 | 0.673464 | Up |
| HBG2 | 50.33362 | 212.1642 | 4.215159 | 2.075587 | 0.045332 | 0.922629 | Up |
| HIP1 | 2536.781 | 5551.817 | 2.188529 | 1.129961 | 4.38E-07 | 0.00151 | Up |
| HLA-DQB1 | 417.9968 | 2300.678 | 5.504056 | 2.460495 | 0.003237 | 0.395766 | Up |
| HLA-DRB5 | 681.282 | 1918.208 | 2.815586 | 1.493435 | 0.010513 | 0.640233 | Up |
| HOXA9 | 15.83021 | 4.670251 | 0.295021 | -1.76111 | 0.048617 | 0.929754 | Down |
| HRASLS | 8.175833 | 23.4832 | 2.87227 | 1.522191 | 0.023402 | 0.761721 | Up |
| IL17RE | 34.51839 | 15.26834 | 0.442325 | -1.17682 | 0.018794 | 0.723979 | Down |
| IL23R | 86.26338 | 17.14087 | 0.198704 | -2.33131 | 7.47E-05 | 0.061265 | Down |
| INHBB | 10.26309 | 24.06026 | 2.34435 | 1.229188 | 0.048132 | 0.929754 | Up |
| IQCA1 | 11.82008 | 1.981241 | 0.167616 | -2.57676 | 0.015966 | 0.696005 | Down |
| JAG2 | 11.05355 | 27.838 | 2.518467 | 1.332546 | 0.027848 | 0.806381 | Up |
| KIAA1217 | 10.5262 | 0.654114 | 0.062142 | -4.0083 | 0.002143 | 0.341976 | Down |
| KRT17 | 0 | 3.75648 | Inf | Inf | 0.047246 | 0.929754 | Up |
| LOC100289279 | 157.6612 | 378.3106 | 2.399516 | 1.262744 | 0.02578 | 0.786977 | Up |
| LOC101929599 | 28.49104 | 10.05327 | 0.352857 | -1.50284 | 0.036929 | 0.862169 | Down |
| LOC102724642 | 53.00436 | 12.47598 | 0.235377 | -2.08696 | 0.047812 | 0.929754 | Down |
| LOC107986351 | 223.9658 | 74.16801 | 0.331158 | -1.59441 | 0.004524 | 0.448161 | Down |
| LOC388282 | 0.37569 | 9.260335 | 24.6489 | 4.623451 | 0.043864 | 0.907815 | Up |
| LOC390937 | 11.10277 | 29.17016 | 2.627287 | 1.393574 | 0.019487 | 0.730506 | Up |
| LOC646652 | 28.15919 | 1.338452 | 0.047532 | -4.39497 | 0.003666 | 0.414982 | Down |
| LTK | 422.0167 | 154.5522 | 0.366223 | -1.44921 | 8.37E-05 | 0.062666 | Down |
| MATN2 | 27.66341 | 10.83074 | 0.391519 | -1.35285 | 0.019803 | 0.730506 | Down |
| ME1 | 78.86561 | 33.38444 | 0.423308 | -1.24022 | 0.000865 | 0.225762 | Down |
| MEF2B | 6.845679 | 23.14038 | 3.38029 | 1.757147 | 0.020577 | 0.73214 | Up |
| MFSD2B | 48.7528 | 109.5972 | 2.248019 | 1.168654 | 0.009835 | 0.623922 | Up |
| MINDY4 | 33.80835 | 16.49812 | 0.487989 | -1.03508 | 0.047814 | 0.929754 | Down |
| MKI67 | 505.0215 | 237.2314 | 0.469745 | -1.09005 | 0.02678 | 0.794858 | Down |
| MMP1 | 2.579692 | 12.24652 | 4.74728 | 2.247101 | 0.023652 | 0.764335 | Up |
| MMRN1 | 353.0193 | 752.8408 | 2.132577 | 1.092598 | 0.005498 | 0.499402 | Up |
| MUC1 | 5.04557 | 17.11684 | 3.392448 | 1.762327 | 0.034405 | 0.849544 | Up |
| MXRA8 | 23.05285 | 6.966914 | 0.302215 | -1.72635 | 0.013169 | 0.680128 | Down |
| MYL4 | 10.72595 | 75.05973 | 6.997955 | 2.806933 | 0.000106 | 0.072704 | Up |
| MYOF | 1168.929 | 543.7164 | 0.465141 | -1.10426 | 0.012837 | 0.680128 | Down |
| MYRFL | 32.36655 | 15.61467 | 0.482432 | -1.0516 | 0.045925 | 0.925974 | Down |
| NAP1L3 | 35.23237 | 73.00882 | 2.072209 | 1.051169 | 0.00495 | 0.468352 | Up |
| NECTIN2 | 52.02088 | 121.0264 | 2.326496 | 1.218159 | 0.001018 | 0.254065 | Up |
| NEK2 | 11.1681 | 2.630131 | 0.235504 | -2.08618 | 0.048664 | 0.929754 | Down |
| NFIB | 10.57102 | 27.79674 | 2.629522 | 1.394801 | 0.016503 | 0.704211 | Up |
| NHSL1 | 116.7014 | 55.78619 | 0.478025 | -1.06484 | 0.01526 | 0.685801 | Down |
| NR4A2 | 115.149 | 384.2744 | 3.337191 | 1.738634 | 0.008411 | 0.581186 | Up |
| NRGN | 813.9603 | 1839.38 | 2.259791 | 1.176189 | 0.020337 | 0.73214 | Up |
| NRXN1 | 33.78087 | 83.93754 | 2.484765 | 1.313109 | 0.014566 | 0.685277 | Up |
| OCA2 | 15.61834 | 3.598224 | 0.230385 | -2.11788 | 0.016032 | 0.696446 | Down |
| OR1J2 | 1.726322 | 9.886239 | 5.726764 | 2.51772 | 0.037049 | 0.862169 | Up |
| OR2W3 | 23.82259 | 50.89017 | 2.136214 | 1.095057 | 0.038994 | 0.875496 | Up |
| OR6N2 | 2.18436 | 10.5996 | 4.852495 | 2.278727 | 0.042155 | 0.901259 | Up |
| OSM | 285.6389 | 657.8819 | 2.303195 | 1.203636 | 0.006324 | 0.531143 | Up |
| PARD6G | 3.44675 | 12.36641 | 3.587846 | 1.843118 | 0.047951 | 0.929754 | Up |
| PCDHGB1 | 9.883761 | 1.784204 | 0.180519 | -2.46978 | 0.036779 | 0.862169 | Down |
| PCDHGC5 | 0.627083 | 7.470221 | 11.91264 | 3.574421 | 0.029136 | 0.814468 | Up |
| PCSK6 | 205.6827 | 423.9098 | 2.060989 | 1.043337 | 0.045467 | 0.922629 | Up |
| PDZK1 | 16.10957 | 4.497027 | 0.279152 | -1.84087 | 0.026454 | 0.79219 | Down |
| PDZK1IP1 | 49.18684 | 151.6182 | 3.082494 | 1.624098 | 0.001906 | 0.326416 | Up |
| PF4 | 1288.636 | 4100.423 | 3.181987 | 1.669928 | 0.003602 | 0.414982 | Up |
| PGRMC1 | 745.3633 | 1600.353 | 2.147078 | 1.102375 | 0.0293 | 0.817689 | Up |
| PHF24 | 31.98652 | 72.42917 | 2.264366 | 1.179107 | 0.008016 | 0.570376 | Up |
| PLA2G4B | 26.93357 | 54.89191 | 2.038048 | 1.027188 | 0.016491 | 0.704211 | Up |
| PLA2G4C | 105.881 | 48.91548 | 0.461986 | -1.11408 | 0.014987 | 0.685801 | Down |
| PLOD2 | 30.4428 | 86.42661 | 2.838984 | 1.505375 | 0.030499 | 0.819614 | Up |
| PODXL | 46.26814 | 21.43223 | 0.463218 | -1.11024 | 0.018222 | 0.722959 | Down |
| PROCR | 40.55863 | 18.93055 | 0.466745 | -1.09929 | 0.028651 | 0.811413 | Down |
| PROS1 | 186.4283 | 470.697 | 2.524815 | 1.336178 | 0.032671 | 0.836077 | Up |
| PRR29 | 28.86153 | 13.17322 | 0.456428 | -1.13154 | 0.048823 | 0.929754 | Down |
| PTGES | 4.234113 | 16.08221 | 3.798246 | 1.925334 | 0.020393 | 0.73214 | Up |
| PTGES3L | 1.962134 | 12.03168 | 6.131937 | 2.616343 | 0.017001 | 0.709883 | Up |
| PTPRB | 37.44342 | 10.42185 | 0.278336 | -1.8451 | 0.008266 | 0.577804 | Down |
| PVALB | 12.09238 | 49.03144 | 4.054738 | 2.019609 | 0.036175 | 0.862169 | Up |
| RAB6C | 1.570417 | 10.36642 | 6.601059 | 2.722698 | 0.024085 | 0.772296 | Up |
| RARRES1 | 0 | 4.149929 | Inf | Inf | 0.029478 | 0.819065 | Up |
| RBMS3 | 34.91978 | 10.51168 | 0.301023 | -1.73205 | 0.001857 | 0.326416 | Down |
| REEP1 | 3.995649 | 20.61847 | 5.160231 | 2.367436 | 0.002018 | 0.337369 | Up |
| RHD | 20.94813 | 51.40381 | 2.453862 | 1.295054 | 0.01521 | 0.685801 | Up |
| RNF222 | 7.79106 | 22.55859 | 2.895445 | 1.533785 | 0.030178 | 0.819185 | Up |
| ROR2 | 34.36478 | 2.021608 | 0.058828 | -4.08736 | 1.58E-07 | 0.000863 | Down |
| RORC | 351.6689 | 163.8318 | 0.465869 | -1.102 | 0.011056 | 0.652663 | Down |
| RPP25 | 54.22656 | 18.30323 | 0.337533 | -1.5669 | 0.000492 | 0.156871 | Down |
| RPS16 | 4210.82 | 2067.463 | 0.490988 | -1.02624 | 0.026635 | 0.793986 | Down |
| SASH1 | 287.1542 | 131.875 | 0.459248 | -1.12265 | 0.015588 | 0.690816 | Down |
| SCRN1 | 1199.63 | 592.7097 | 0.494077 | -1.01719 | 6.53E-07 | 0.001873 | Down |
| SEMA5A | 16.65477 | 38.609 | 2.318195 | 1.213002 | 0.017404 | 0.709883 | Up |
| SH3BGRL2 | 1441.312 | 3081.02 | 2.137649 | 1.096025 | 0.013555 | 0.680128 | Up |
| SH3RF1 | 134.2555 | 63.01974 | 0.469402 | -1.09111 | 0.018965 | 0.724547 | Down |
| SKOR1 | 12.20731 | 27.76976 | 2.274846 | 1.185769 | 0.046888 | 0.929754 | Up |
| SLC24A3 | 103.1031 | 235.7399 | 2.286449 | 1.193109 | 0.049787 | 0.930825 | Up |
| SLC28A3 | 21.21041 | 8.229666 | 0.388001 | -1.36587 | 0.047048 | 0.929754 | Down |
| SLC47A1 | 59.97103 | 23.21114 | 0.387039 | -1.36945 | 0.014027 | 0.680128 | Down |
| SLC4A10 | 1111.77 | 202.4863 | 0.18213 | -2.45696 | 2.47E-06 | 0.005322 | Down |
| SOGA3 | 18.37004 | 47.25321 | 2.572298 | 1.363058 | 0.003246 | 0.395766 | Up |
| SOX5 | 98.7077 | 42.54856 | 0.431056 | -1.21405 | 0.030982 | 0.823277 | Down |
| SPACA6 | 110.0495 | 231.6224 | 2.10471 | 1.073622 | 0.000127 | 0.084288 | Up |
| SPP1 | 7.261698 | 30.21997 | 4.161557 | 2.057123 | 0.001496 | 0.302983 | Up |
| SPTB | 175.4557 | 415.9918 | 2.370923 | 1.245449 | 0.043104 | 0.906246 | Up |
| SPX | 71.64646 | 152.1275 | 2.123308 | 1.086314 | 0.045166 | 0.921469 | Up |
| TAS2R31 | 189.695 | 91.7694 | 0.483773 | -1.0476 | 6.56E-05 | 0.059436 | Down |
| TBC1D3B | 18.12458 | 4.756618 | 0.26244 | -1.92994 | 0.018585 | 0.723979 | Down |
| TGFB1I1 | 17.04074 | 45.27734 | 2.657006 | 1.409801 | 0.01952 | 0.730506 | Up |
| THBS1 | 2236.367 | 5544.716 | 2.479341 | 1.309956 | 0.000506 | 0.158289 | Up |
| TMEM171 | 20.09325 | 3.079466 | 0.153259 | -2.70596 | 0.006695 | 0.54381 | Down |
| TMEM191B | 5.932638 | 26.9004 | 4.534307 | 2.180882 | 0.035063 | 0.85594 | Up |
| TMEM51 | 17.4709 | 3.315714 | 0.189785 | -2.39756 | 0.004824 | 0.461706 | Down |
| TMEM56 | 15.42382 | 40.45458 | 2.622864 | 1.391143 | 0.008692 | 0.589236 | Up |
| TMIGD3 | 21.61198 | 51.75313 | 2.39465 | 1.259815 | 0.027356 | 0.801093 | Up |
| TNFRSF13B | 36.40679 | 15.71746 | 0.431718 | -1.21184 | 0.022562 | 0.751439 | Down |
| TNFRSF17 | 61.695 | 21.56263 | 0.349504 | -1.51662 | 0.021691 | 0.744004 | Down |
| TRIM73 | 7.368828 | 20.63508 | 2.80032 | 1.485592 | 0.038515 | 0.875496 | Up |
| TRPC6 | 32.10915 | 66.24717 | 2.063187 | 1.044875 | 0.039698 | 0.879662 | Up |
| TTC26 | 200.7313 | 94.26735 | 0.46962 | -1.09044 | 0.028563 | 0.811331 | Down |
| VEPH1 | 67.78615 | 149.1759 | 2.200684 | 1.137952 | 0.004002 | 0.426047 | Up |
| VIL1 | 62.53394 | 125.8181 | 2.011997 | 1.008628 | 0.01067 | 0.643846 | Up |
| WASF3 | 48.19716 | 112.0506 | 2.324838 | 1.21713 | 0.020629 | 0.732387 | Up |
| WNT10A | 14.4247 | 32.31248 | 2.24008 | 1.16355 | 0.030602 | 0.820781 | Up |
| ZFP57 | 0 | 30.83981 | Inf | Inf | 0.001065 | 0.261958 | Up |
| ZMAT4 | 126.9164 | 44.58156 | 0.351267 | -1.50936 | 0.020422 | 0.73214 | Down |
| ZNF556 | 0.198422 | 5.090902 | 25.65698 | 4.681279 | 0.036742 | 0.862169 | Up |
| ZNF704 | 21.54368 | 5.002037 | 0.232181 | -2.10668 | 0.004036 | 0.426047 | Down |

**Note:** TCM, traditional Chinese medicine; CAG, chronic atrophic gastritis; PDHS: Pi-wei damp-heat syndrome; PQDS: Pi-qi-deficiency syndrome.

^a^ The genes marked in brown font denoted the common differential genes observed both in PDHS and PQDS, compared with the healthy control.

^b^ normalized mean count value;

^c^ normalized mean count value (CAG with TCM-defined PQDS, n=5) / normalized mean count value (healthy control, n=5);

^d^ the adjusted *P*-value.

**Table S4** List of the differentially expressed miRNAs in blood leukocytes in PDHS.

| **miRNA_id ^a^** | **Sequence (5’-3’)** | **Length** | **BaseMean_control_A** ^b^ | **BaseMean_case_F** ^b^ | **FoldChange** ^c^  **G(n=5)/A(n=5)** | **log2FoldChange** | ***P*-value** | **q-value** ^d^ | **Regulation** |
| --- | --- | --- | --- | --- | --- | --- | --- | --- | --- |
| hsa-miR-124-5p | CGTGTTCACAGCGGACCTTGAT | 22 | 0 | 1.455029494 | Inf | Inf | 0.036654894 | 1 | Up |
| hsa-miR-125a-3p | ACAGGTGAGGTTCTTGGGAGCC | 22 | 9.948313121 | 23.30657314 | 2.342766342 | 1.228213073 | 0.010600568 | 1 | Up |
| hsa-miR-1297 | TTCAAGTAATTCAGGTG | 17 | 23.00024862 | 8.698229828 | 0.378179818 | -1.402855722 | 0.004224144 | 1 | Down |
| hsa-miR-133a-3p | TTTGGTCCCCTTCAACCAGCTG | 22 | 0.709157152 | 3.821075753 | 5.388193216 | 2.429801585 | 0.02142001 | 1 | Up |
| hsa-miR-133a-5p | AGCTGGTAAAATGGAACCAAAT | 22 | 0.757227261 | 3.287683955 | 4.341740086 | 2.118273363 | 0.029310571 | 1 | Up |
| hsa-miR-1827 | TGAGGCAGTAGATTGAAT | 18 | 1.017834876 | 3.983340712 | 3.913543156 | 1.968475354 | 0.049036426 | 1 | Up |
| hsa-miR-3186-3p | TCACGCGGAGAGATGGCTTTG | 21 | 2.058968011 | 0.245004017 | 0.1189936 | -3.071044108 | 0.027525693 | 1 | Down |
| hsa-miR-3192-5p | TCTGGGAGGTTGTAGCAGTGGAA | 23 | 1.325594904 | 0 | 0 | -Inf | 0.036174092 | 1 | Down |
| hsa-miR-375-3p | TTTGTTCGTTCGGCTCGCGTGA | 22 | 0.839435198 | 6.102186173 | 7.269395168 | 2.861835333 | 0.000821612 | 1 | Up |
| hsa-miR-451b | TAGCAAGAGAACCATTACCATT | 22 | 37.43457426 | 77.22923468 | 2.063045626 | 1.044775728 | 0.025427029 | 1 | Up |
| hsa-miR-452-5p | AACTGTTTGCAGAGGAAACTGA | 22 | 4.163747881 | 12.25502044 | 2.943266689 | 1.55741827 | 0.030349085 | 1 | Up |
| hsa-miR-508-5p | TACTCCAGAGGGCGTCACTCATG | 23 | 0 | 1.36518944 | Inf | Inf | 0.040857255 | 1 | Up |
| hsa-miR-509-3-5p | TACTGCAGACGTGGCAATCATG | 22 | 4.873568321 | 19.80540598 | 4.06384084 | 2.0228439 | 0.021901406 | 1 | Up |
| hsa-miR-548ay-5p | AAAAGTAATTGTGGTTTTTGC | 21 | 9.98618692 | 58.44669681 | 5.852754137 | 2.549115675 | 0.028793796 | 1 | Up |
| hsa-miR-6783-5p | TAGGGGAAAAGTCCTGATCCGG | 22 | 3.277118283 | 0.738423637 | 0.225327124 | -2.149907107 | 0.04487387 | 1 | Down |
| hsa-miR-6853-3p | TGTTCATTGGAACCCTGCGCAG | 22 | 1.904101931 | 0 | 0 | -Inf | 0.014313806 | 1 | Down |
| hsa-miR-6855-5p | TTGGGGTTTGGGGTGCAGACATTGC | 25 | 3.6555128 | 0.625063542 | 0.170992027 | -2.547999041 | 0.026694046 | 1 | Down |
| novel105_mature | TCTCTACCCCTCTCCTCACAGA | 22 | 9.141904702 | 3.058529207 | 0.334561484 | -1.579656727 | 0.017068419 | 1 | Down |
| novel1287_mature | TGAGCTACCACGCCAGGCCGATCT | 24 | 0.30795338 | 3.139404291 | 10.19441414 | 3.349706963 | 0.041931195 | 1 | Up |
| novel1292_mature | GGCGGCGGCGGCGGCGGCGGCGGGA | 25 | 0.992649703 | 3.359878147 | 3.384757118 | 1.759052313 | 0.047668314 | 1 | Up |
| novel1632_mature | TTTCCTTCCCCTTGCACCCAGA | 22 | 1.525834617 | 0 | 0 | -Inf | 0.03390798 | 1 | Down |
| novel1852_mature | AATTACTTTTAATACCACTGTACA | 24 | 0.283492551 | 2.371906421 | 8.366732785 | 3.064664359 | 0.040102577 | 1 | Up |
| novel193_star | TTGGTAACTGTACCCAGGGTCC | 22 | 1.444351025 | 0 | 0 | -Inf | 0.026067524 | 1 | Down |
| novel1945_mature | TCCGGTTCTCAAGGCTCCAT | 20 | 12.95315805 | 5.70963479 | 0.440790946 | -1.181833504 | 0.043952649 | 1 | Down |
| novel224_mature | AAGAGTTACTAGAACTATTCA | 21 | 0.14217205 | 2.575106569 | 18.11260775 | 4.178922367 | 0.012334027 | 1 | Up |
| novel396_mature | TTGTATCAGTGGCTTTAATTCC | 22 | 3.039310388 | 0.268401692 | 0.088310063 | -3.501278344 | 0.024708895 | 1 | Down |
| novel579_star | TTCTCCCAACGTAAACCCAGC | 21 | 5.984851383 | 1.138360903 | 0.190207046 | -2.394357404 | 0.009456174 | 1 | Down |
| novel769_mature | TTAGGGCCCTGGCTCCATCC | 20 | 0.721530625 | 3.777244579 | 5.235044011 | 2.388201666 | 0.047202607 | 1 | Up |
| novel78_mature | TTCGCTGGGAATTCAGCCTCT | 21 | 1.290412176 | 5.437984034 | 4.214145011 | 2.075239959 | 0.020270934 | 1 | Up |
| novel93_mature | TAGGCCATTTTGGAAGCTGTTT | 22 | 3.203129121 | 7.548643933 | 2.356646781 | 1.236735541 | 0.021700131 | 1 | Up |
| hsa-miR-124-5p | CGTGTTCACAGCGGACCTTGAT | 22 | 0 | 1.455029494 | Inf | Inf | 0.036654894 | 1 | Up |
| hsa-miR-125a-3p | CGTGTTCACAGCGGACCTTGAT | 22 | 9.948313121 | 23.30657314 | 2.342766342 | 1.228213073 | 0.010600568 | 1 | Up |
| hsa-miR-1297 | ACAGGTGAGGTTCTTGGGAGCC | 22 | 23.00024862 | 8.698229828 | 0.378179818 | -1.402855722 | 0.004224144 | 1 | Down |
| hsa-miR-133a-3p | TTCAAGTAATTCAGGTG | 17 | 0.709157152 | 3.821075753 | 5.388193216 | 2.429801585 | 0.02142001 | 1 | Up |
| hsa-miR-133a-5p | TTTGGTCCCCTTCAACCAGCTG | 22 | 0.757227261 | 3.287683955 | 4.341740086 | 2.118273363 | 0.029310571 | 1 | Up |
| hsa-miR-1827 | AGCTGGTAAAATGGAACCAAAT | 22 | 1.017834876 | 3.983340712 | 3.913543156 | 1.968475354 | 0.049036426 | 1 | Up |
| hsa-miR-3186-3p | TGAGGCAGTAGATTGAAT | 18 | 2.058968011 | 0.245004017 | 0.1189936 | -3.071044108 | 0.027525693 | 1 | Down |
| hsa-miR-3192-5p | TCACGCGGAGAGATGGCTTTG | 21 | 1.325594904 | 0 | 0 | -Inf | 0.036174092 | 1 | Down |
| hsa-miR-375-3p | TCTGGGAGGTTGTAGCAGTGGAA | 23 | 0.839435198 | 6.102186173 | 7.269395168 | 2.861835333 | 0.000821612 | 1 | Up |
| hsa-miR-451b | TTTGTTCGTTCGGCTCGCGTGA | 22 | 37.43457426 | 77.22923468 | 2.063045626 | 1.044775728 | 0.025427029 | 1 | Up |
| hsa-miR-452-5p | TAGCAAGAGAACCATTACCATT | 22 | 4.163747881 | 12.25502044 | 2.943266689 | 1.55741827 | 0.030349085 | 1 | Up |
| hsa-miR-508-5p | AACTGTTTGCAGAGGAAACTGA | 22 | 0 | 1.36518944 | Inf | Inf | 0.040857255 | 1 | Up |
| hsa-miR-509-3-5p | TACTCCAGAGGGCGTCACTCATG | 23 | 4.873568321 | 19.80540598 | 4.06384084 | 2.0228439 | 0.021901406 | 1 | Up |
| hsa-miR-548ay-5p | TACTGCAGACGTGGCAATCATG | 22 | 9.98618692 | 58.44669681 | 5.852754137 | 2.549115675 | 0.028793796 | 1 | Up |
| hsa-miR-6783-5p | AAAAGTAATTGTGGTTTTTGC | 21 | 3.277118283 | 0.738423637 | 0.225327124 | -2.149907107 | 0.04487387 | 1 | Down |
| hsa-miR-6853-3p | TAGGGGAAAAGTCCTGATCCGG | 22 | 1.904101931 | 0 | 0 | -Inf | 0.014313806 | 1 | Down |
| hsa-miR-6855-5p | TGTTCATTGGAACCCTGCGCAG | 22 | 3.6555128 | 0.625063542 | 0.170992027 | -2.547999041 | 0.026694046 | 1 | Down |
| novel105_mature | TTGGGGTTTGGGGTGCAGACATTGC | 25 | 9.141904702 | 3.058529207 | 0.334561484 | -1.579656727 | 0.017068419 | 1 | Down |
| novel1287_mature | TCTCTACCCCTCTCCTCACAGA | 22 | 0.30795338 | 3.139404291 | 10.19441414 | 3.349706963 | 0.041931195 | 1 | Up |
| novel1292_mature | TGAGCTACCACGCCAGGCCGATCT | 24 | 0.992649703 | 3.359878147 | 3.384757118 | 1.759052313 | 0.047668314 | 1 | Up |
| novel1632_mature | GGCGGCGGCGGCGGCGGCGGCGGGA | 25 | 1.525834617 | 0 | 0 | -Inf | 0.03390798 | 1 | Down |
| novel1852_mature | TTTCCTTCCCCTTGCACCCAGA | 22 | 0.283492551 | 2.371906421 | 8.366732785 | 3.064664359 | 0.040102577 | 1 | Up |
| novel193_star | AATTACTTTTAATACCACTGTACA | 24 | 1.444351025 | 0 | 0 | -Inf | 0.026067524 | 1 | Down |
| novel1945_mature | TTGGTAACTGTACCCAGGGTCC | 22 | 12.95315805 | 5.70963479 | 0.440790946 | -1.181833504 | 0.043952649 | 1 | Down |
| novel224_mature | TCCGGTTCTCAAGGCTCCAT | 20 | 0.14217205 | 2.575106569 | 18.11260775 | 4.178922367 | 0.012334027 | 1 | Up |
| novel396_mature | AAGAGTTACTAGAACTATTCA | 21 | 3.039310388 | 0.268401692 | 0.088310063 | -3.501278344 | 0.024708895 | 1 | Down |
| novel579_star | TTGTATCAGTGGCTTTAATTCC | 22 | 5.984851383 | 1.138360903 | 0.190207046 | -2.394357404 | 0.009456174 | 1 | Down |
| novel769_mature | TTCTCCCAACGTAAACCCAGC | 21 | 0.721530625 | 3.777244579 | 5.235044011 | 2.388201666 | 0.047202607 | 1 | Up |
| novel78_mature | TTAGGGCCCTGGCTCCATCC | 20 | 1.290412176 | 5.437984034 | 4.214145011 | 2.075239959 | 0.020270934 | 1 | Up |
| novel93_mature | TTCGCTGGGAATTCAGCCTCT | 21 | 3.203129121 | 7.548643933 | 2.356646781 | 1.236735541 | 0.021700131 | 1 | Up |

**Note:** TCM, traditional Chinese medicine; CAG, chronic atrophic gastritis; PDHS: Pi-wei damp-heat syndrome; PQDS: Pi-qi-deficiency syndrome.

^a^ The miRNAs marked in brown font denoted the common differential miRNAs observed both in PDHS and PQDS, compared with the healthy control.

^b^ normalized mean count value;

^c^ normalized mean count value (CAG with TCM-defined PDHS, n=5) / normalized mean count value (healthy control, n=5);

^d^ the adjusted *P*-value.

**Table S5** List of the differentially expressed miRNAs identified in blood leukocytes in PQDS.

| **miRNA_id a** | **Sequence (5’-3’)** | **Length** | **BaseMean_control_A ^b^** | **BaseMean_case_F ^b^** | **FoldChange ^c^**  **F(n=5)/A(n=5)** | **log2FoldChange** | ***P*-value** | **q-value ^d^** | **Regulation** |
| --- | --- | --- | --- | --- | --- | --- | --- | --- | --- |
| hsa-miR-122-5p | TGGAGTGTGACAATGGTGTTTG | 22 | 3.541765 | 241.9149 | 68.3035 | 6.093888 | 0.00166 | 1 | Up |
| hsa-miR-125a-3p | ACAGGTGAGGTTCTTGGGAGCC | 22 | 9.779214 | 28.13562 | 2.877084 | 1.524608 | 0.014186 | 1 | Up |
| hsa-miR-126-3p | TCGTACCGTGAGTAATAATGCG | 22 | 45442.28 | 105352.5 | 2.318381 | 1.213118 | 0.034927 | 1 | Up |
| hsa-miR-1260b | ATCCCACCACTGCCACCAT | 19 | 9.000838 | 23.24618 | 2.582668 | 1.368862 | 0.040237 | 1 | Up |
| hsa-miR-1297 | TTCAAGTAATTCAGGTG | 17 | 22.66883 | 4.476239 | 0.197462 | -2.34035 | 0.000847 | 1 | Down |
| hsa-miR-133a-3p | TTTGGTCCCCTTCAACCAGCTG | 22 | 0.692149 | 22.95864 | 33.17009 | 5.051811 | 0.008733 | 1 | Up |
| hsa-miR-133b | TTTGGTCCCCTTCAACCAGCTA | 22 | 1.254933 | 8.609528 | 6.86055 | 2.778324 | 0.041725 | 1 | Up |
| hsa-miR-136-5p | ACTCCATTTGTTTTGATGATGGA | 23 | 14.076 | 33.5581 | 2.384066 | 1.253424 | 0.043738 | 1 | Up |
| hsa-miR-1910-5p | CCAGTCCTGTGCCTGCCGCCT | 21 | 1.454216 | 0 | 0 | -Inf | 0.031801 | 1 | Down |
| hsa-miR-205-5p | TCCTTCATTCCACCGGAGTCTG | 22 | 16.03169 | 0.377651 | 0.023557 | -5.40773 | 0.027346 | 1 | Down |
| hsa-miR-299-5p | TGGTTTACCGTCCCACATACAT | 22 | 2.06622 | 8.07903 | 3.910054 | 1.967188 | 0.025791 | 1 | Up |
| hsa-miR-337-3p | CTCCTATATGATGCCTTTCTTC | 22 | 30.56926 | 110.1613 | 3.603664 | 1.849465 | 0.046851 | 1 | Up |
| hsa-miR-375-3p | TTTGTTCGTTCGGCTCGCGTGA | 22 | 0.822789 | 4.343459 | 5.278948 | 2.400251 | 0.014716 | 1 | Up |
| hsa-miR-449a | TGGCAGTGTATTGTTAGCTGGT | 22 | 0.558875 | 2.755649 | 4.930704 | 2.301794 | 0.034553 | 1 | Up |
| hsa-miR-4701-5p | TTGGCCACCACACCTACCCCTT | 22 | 0.571456 | 3.50866 | 6.139859 | 2.618205 | 0.019945 | 1 | Up |
| hsa-miR-495-3p | AAACAAACATGGTGCACTTCTT | 22 | 165.199 | 780.9101 | 4.727088 | 2.240952 | 0.04125 | 1 | Up |
| hsa-miR-548ay-5p | AAAAGTAATTGTGGTTTTTGC | 21 | 9.752576 | 36.72075 | 3.765236 | 1.91274 | 0.022415 | 1 | Up |
| hsa-miR-548d-5p | AAAAGTAATTGTGGTTTTTGCC | 22 | 138.3397 | 50.09009 | 0.36208 | -1.46562 | 0.004377 | 1 | Down |
| hsa-miR-548i | AAAAGTAATTGCGGATTTTGCC | 22 | 3.043883 | 0.54791 | 0.180004 | -2.4739 | 0.040521 | 1 | Down |
| hsa-miR-551b-3p | GCGACCCATACTTGGTTTCAG | 21 | 0 | 1.29602 | Inf | Inf | 0.031221 | 1 | Up |
| hsa-miR-5680 | GAGAAATGCTGGACTAATCTGC | 22 | 1.484213 | 0 | 0 | -Inf | 0.021676 | 1 | Down |
| hsa-miR-574-3p | CACGCTCATGCACACACCCACA | 22 | 398.0662 | 821.1694 | 2.062897 | 1.044672 | 0.035913 | 1 | Up |
| hsa-miR-643 | ACTTGTATGCTAGCTCAGGTAG | 22 | 1.376928 | 0 | 0 | -Inf | 0.032419 | 1 | Down |
| hsa-miR-6509-3p | TTCCACTGCCACTACCTAATTT | 22 | 0.468959 | 3.476099 | 7.412367 | 2.889934 | 0.015087 | 1 | Up |
| hsa-miR-6513-5p | TTTGGGATTGACGCCACATGTCT | 23 | 12.94552 | 4.505367 | 0.348025 | -1.52274 | 0.035934 | 1 | Down |
| hsa-miR-655-3p | ATAATACATGGTTAACCTCTTT | 22 | 12.94224 | 31.70756 | 2.449928 | 1.292739 | 0.044286 | 1 | Up |
| hsa-miR-6802-3p | TTCACCCCTCTCACCTAAGCAG | 22 | 0.373252 | 2.501546 | 6.702023 | 2.744597 | 0.039947 | 1 | Up |
| hsa-miR-6873-3p | TTCTCTCTGTCTTTCTCTCTCAG | 23 | 6.205678 | 1.929201 | 0.310877 | -1.68559 | 0.047787 | 1 | Down |
| hsa-miR-7110-3p | TCTCTCTCCCACTTCCCTGCAG | 22 | 0.349094 | 2.889862 | 8.278182 | 3.049314 | 0.020912 | 1 | Up |
| hsa-miR-877-5p | GTAGAGGAGATGGCGCAGGG | 20 | 6.503221 | 16.72183 | 2.571315 | 1.362506 | 0.046849 | 1 | Up |
| hsa-miR-944 | AAATTATTGTACATCGGATGAG | 22 | 1.954247 | 0 | 0 | -Inf | 0.048266 | 1 | Down |
| novel1022_mature | GAGCTGGGTTTAGAACGC | 18 | 0.141167 | 34.84311 | 246.8226 | 7.947331 | 0.002918 | 1 | Up |
| novel1029_mature | CCGGAGGAAGGTGGGGAC | 18 | 0 | 12.2771 | Inf | Inf | 0.004912 | 1 | Up |
| novel1152_mature | ATGTGCTGTTAAGGGTGG | 18 | 0.324935 | 2.640403 | 8.125936 | 3.022534 | 0.02875 | 1 | Up |
| novel1238_mature | TTCCCCGCTTCCCCCCTAGGGG | 22 | 1.200853 | 4.698562 | 3.912688 | 1.96816 | 0.029463 | 1 | Up |
| novel1316_mature | CCCACCTGACCCCATGCT | 18 | 0 | 10.8898 | Inf | Inf | 0.006733 | 1 | Up |
| novel134_mature | AACTGGGCATGTTGGAACTAAGCT | 24 | 5.892368 | 1.319633 | 0.223956 | -2.15871 | 0.010955 | 1 | Down |
| novel1381_mature | AAGGCGGCCCCCTGGAAG | 18 | 0 | 5.482911 | Inf | Inf | 0.022367 | 1 | Up |
| novel145_star | AAACCGTAATTACTTGTGTACA | 22 | 0 | 1.984512 | Inf | Inf | 0.011351 | 1 | Up |
| novel1495_mature | TCGGCCGCCGCTTTCCCTCGGA | 22 | 0.514419 | 3.74106 | 7.272401 | 2.862432 | 0.011573 | 1 | Up |
| novel1519_mature | TTACTGTGATGAACTTCTGAGGGT | 24 | 7.602652 | 2.427537 | 0.319301 | -1.64701 | 0.041825 | 1 | Down |
| novel1621_mature | CCTGTGAGGGCTGCTGGC | 18 | 0 | 7.80467 | Inf | Inf | 0.013017 | 1 | Up |
| novel1646_mature | CCGCCGGGCCAAGATGGCTGCA | 22 | 1.300468 | 0 | 0 | -Inf | 0.049299 | 1 | Down |
| novel1678_mature | TTGGTGGGGCTTGCTCTGG | 19 | 0 | 4.609576 | Inf | Inf | 0.004636 | 1 | Up |
| novel1679_mature | ACTATCTGCCGTGGGCGC | 18 | 0 | 6.082619 | Inf | Inf | 0.038911 | 1 | Up |
| novel1687_star | ATATAGTAACAAGCTGGATAAAG | 23 | 0.186626 | 2.25582 | 12.08737 | 3.595429 | 0.014523 | 1 | Up |
| novel1702_mature | CAGGTGCTGCATGGCTGCC | 19 | 0.360671 | 69.72484 | 193.3195 | 7.594844 | 0.001545 | 1 | Up |
| novel1796_mature | CTGGCTCCTCCCCCCACCCAGG | 22 | 0.141167 | 2.865935 | 20.3018 | 4.343535 | 0.044548 | 1 | Up |
| novel1800_mature | GTCTGCACCAGGATGGCT | 18 | 0 | 5.46115 | Inf | Inf | 0.02017 | 1 | Up |
| novel1803_mature | TCGGCCGTGTATCTCTGTGCC | 21 | 2.60407 | 8.095469 | 3.108775 | 1.636346 | 0.047659 | 1 | Up |
| novel1854_mature | CCGAGAGGCTGAAGGAGC | 18 | 0 | 5.680516 | Inf | Inf | 0.022656 | 1 | Up |
| novel2005_mature | AGCTGGTCTGAGAGGATG | 18 | 0 | 3.684313 | Inf | Inf | 0.027197 | 1 | Up |
| novel214_mature | TCCTCCACCACCTCCTCCTGC | 21 | 0.162468 | 1.731539 | 10.65775 | 3.413831 | 0.046923 | 1 | Up |
| novel224_mature | AAGAGTTACTAGAACTATTCA | 21 | 0.141167 | 2.136294 | 15.13314 | 3.91964 | 0.021217 | 1 | Up |
| novel255_mature | CCCGCCCCTCCGCGCCCCCCC | 21 | 0.557824 | 3.321704 | 5.95475 | 2.574041 | 0.035001 | 1 | Up |
| novel26_star | AGGTAGGATTAGATCATTAGCTCA | 24 | 7.254237 | 1.945881 | 0.268241 | -1.8984 | 0.030984 | 1 | Down |
| novel275_star | CTCCCCCAGAAGGCTGTCCTGAGG | 24 | 1.638788 | 0 | 0 | -Inf | 0.020647 | 1 | Down |
| novel27_star | AGGCCACTCCTCATGCCCCCAGT | 23 | 0.480537 | 2.937121 | 6.112164 | 2.611683 | 0.046314 | 1 | Up |
| novel35_star | TTCCTTGGGAGGGAGCATGTAGC | 23 | 0.327793 | 3.739366 | 11.40772 | 3.511938 | 0.008365 | 1 | Up |
| novel47_mature | AGCCTCCCAGTCTGGCCTGAGT | 22 | 2.570112 | 8.175885 | 3.181139 | 1.669544 | 0.041761 | 1 | Up |
| novel55_star | TCAGGGAGAAAGAAGGGTTATT | 22 | 1.53932 | 5.741149 | 3.729666 | 1.899046 | 0.024117 | 1 | Up |
| novel567_mature | CATCTTCCCAAGCCTCCTCCATC | 23 | 0.688464 | 4.678149 | 6.79505 | 2.764484 | 0.009126 | 1 | Up |
| novel653_mature | TGCCTTGCCCTCTCCCTCTAGG | 22 | 5.769173 | 15.2602 | 2.645128 | 1.403337 | 0.029714 | 1 | Up |
| novel803_mature | GTAGGTGGGTGGTTTTGG | 18 | 0 | 4.494887 | Inf | Inf | 0.025401 | 1 | Up |
| novel846_mature | CCTGTCTGAGCGCCGCTC | 18 | 9.602415 | 3.973322 | 0.413784 | -1.27305 | 0.043792 | 1 | Down |
| novel908_mature | TTGGGAGTAAAGTGGGGGG | 19 | 0 | 8.581303 | Inf | Inf | 0.008502 | 1 | Up |
| novel919_mature | GGTGGTTCAGGCAGGGGAC | 19 | 0 | 36.45573 | Inf | Inf | 0.001059 | 1 | Up |
| novel92_mature | ACATGTTGACTCTCCTACCTGG | 22 | 2.805859 | 0.39521 | 0.140852 | -2.82775 | 0.030253 | 1 | Down |
| novel942_mature | TGTTGTTGGGTAGGGGAGG | 19 | 0 | 159.1787 | Inf | Inf | 7.19E-05 | 0.27544 | Up |
| novel955_mature | AATTTGGTGGCCACTGCTTCAGT | 23 | 1.609841 | 0 | 0 | -Inf | 0.03072 | 1 | Down |
| novel982_mature | AGGAAGGTGGGGATGACGG | 19 | 0 | 6.223831 | Inf | Inf | 0.009948 | 1 | Up |
| novel987_mature | TGGCCTTGTTGGTCTGAGACTTGT | 24 | 0.53572 | 3.320544 | 6.198283 | 2.631869 | 0.026291 | 1 | Up |

**Note:** TCM, traditional Chinese medicine; CAG, chronic atrophic gastritis; PDHS: Pi-wei damp-heat syndrome; PQDS: Pi-qi-deficiency syndrome.

^a^ The miRNAs marked in brown font denoted the common differential miRNAs observed both in PDHS and PQDS, compared with the healthy control.

^b^ normalized mean count value;

^c^ normalized mean count value (CAG with TCM-defined PQDS, n=5) / normalized mean count value (healthy control, n=5);

^d^ the adjusted *P*-value.

**Table S6** List of the differentially expressed miRNAs in serums in PDHS.

| **miRNA_id** ^a^ | **Sequence** | **Length** | **FoldChange** ^b^  **G(n=3)/A(n=3)** | **log2FoldChange** | ***P*-value** | **q-value ^c^** | **Regulation** |
| --- | --- | --- | --- | --- | --- | --- | --- |
| hsa-miR-10b-3p | ACAGATTCGATTCTAGGGGAAT | 22 | 0.002974 | -8.39346 | 0.000962 | 0.063013 | Down |
| hsa-miR-1249-3p | ACGCCCTTCCCCCCCTTCTTCA | 22 | 0.006734 | -7.21429 | 0.006166 | 0.161551 | Down |
| hsa-miR-130a-3p | CAGTGCAATGTTAAAAGGGCAT | 22 | 0.012804 | -6.28725 | 0.03323 | 0.356212 | Down |
| hsa-miR-132-3p | TAACAGTCTACAGCCATGGTCG | 22 | 0.009551 | -6.71015 | 0.035408 | 0.356212 | Down |
| hsa-miR-142-3p | TGTAGTGTTTCCTACTTTATGGA | 23 | 0.003011 | -8.37555 | 0.002353 | 0.095906 | Down |
| hsa-miR-152-3p | TCAGTGCATGACAGAACTTGG | 21 | 0.014856 | -6.07282 | 0.040391 | 0.356212 | Down |
| hsa-miR-181c-5p | AACATTCAACCTGTCGGTGAGT | 22 | 0.010266 | -6.60601 | 0.037657 | 0.356212 | Down |
| hsa-miR-181d-5p | AACATTCATTGTTGTCGGTGGGT | 23 | 0.007892 | -6.98538 | 0.027844 | 0.331597 | Down |
| hsa-miR-191-3p | GCTGCGCTTGGATTTCGTCCCC | 22 | 0.010205 | -6.61452 | 0.037472 | 0.356212 | Down |
| hsa-miR-29c-3p | TAGCACCATTTGAAATCGGTTA | 22 | 0.008753 | -6.83606 | 0.017847 | 0.269759 | Down |
| hsa-miR-302c-3p | TAAGTGCTTCCATGTTTCAGTGG | 23 | 3577699 | 21.7706 | 1.92E-09 | 4.62E-07 | Up |
| hsa-miR-369-3p | AATAATACATGGTTGATCTTT | 21 | 0.015252 | -6.03484 | 0.042232 | 0.356212 | Down |
| hsa-miR-409-5p | AGGTTACCCGAGCAACTTTGCAT | 23 | 0.002426 | -8.68717 | 0.002873 | 0.095906 | Down |
| hsa-miR-411-3p | TATGTAACACGGTCCACTAACC | 22 | 0.006397 | -7.28828 | 0.023446 | 0.307143 | Down |
| hsa-miR-421 | ATCAACAGACATTAATTGGGCGC | 23 | 0.009131 | -6.77505 | 0.007301 | 0.167332 | Down |
| hsa-miR-429 | TAATACTGTCTGGTAAAACCGT | 22 | 47.65909 | 5.57468 | 0.041417 | 0.356212 | Up |
| hsa-miR-433-3p | ATCATGATGGGCTCCTCGGTGT | 22 | 0.003231 | -8.274 | 0.008965 | 0.176162 | Down |
| hsa-miR-450a-5p | TTTTGCGATGTGTTCCTAATAT | 22 | 0.008907 | -6.81084 | 0.009684 | 0.181229 | Down |
| hsa-miR-543 | AAACATTCGCGGTGCACTTCTT | 22 | 0.011978 | -6.38342 | 0.046246 | 0.356212 | Down |
| hsa-miR-548d-5p | AAAAGTAATTGTGGTTTTTGCC | 22 | 144.4755 | 7.174681 | 0.046842 | 0.356212 | Up |
| hsa-miR-656-3p | AATATTATACAGTCAACCTCT | 21 | 0.004313 | -7.85716 | 0.015037 | 0.256929 | Down |
| hsa-miR-6749-3p | CTCCTCCCCTGCCTGGCCCAG | 21 | 144.4755 | 7.174681 | 0.046842 | 0.356212 | Up |
| hsa-miR-885-3p | AGGCAGCGGGGTGTAGTGGATA | 22 | 42.59598 | 5.412646 | 0.047132 | 0.356212 | Up |
| hsa-miR-9-5p | TCTTTGGTTATCTAGCTGTATGA | 23 | 0.002839 | -8.46048 | 0.00765 | 0.167332 | Down |
| novel10_mature | TCCTGTTTCCTGGCACCC | 18 | 238.2659 | 7.896428 | 0.01756 | 0.269759 | Up |
| novel119_mature | ATAGGCCAGGTGTGGAAGCA | 20 | 155.2042 | 7.278023 | 0.025991 | 0.329496 | Up |
| novel163_mature | CCATGGATATCTAGGCTCC | 19 | 234.8302 | 7.875474 | 0.001729 | 0.095906 | Up |
| novel177_mature | GGCTGATGGCTGGGGTGA | 18 | 3.07E-07 | -21.6373 | 2.87E-09 | 4.62E-07 | Down |
| novel282_mature | CAAAGAACTTTGAAAAGAG | 19 | 33.4585 | 5.064301 | 0.045968 | 0.356212 | Up |
| novel377_mature | GAGGGTTGGAGGGGTGAC | 18 | 747.6329 | 9.546186 | 0.002608 | 0.095906 | Up |
| novel380_mature | GGAGGGCGGCGGCGGCGC | 18 | 157.3721 | 7.298036 | 0.043046 | 0.356212 | Up |
| novel38_mature | TAGGAGGGCGTGGAGGTC | 18 | 152.2135 | 7.249952 | 0.044489 | 0.356212 | Up |
| novel399_mature | GAGCCCGGGCTGCGGCAC | 18 | 345.7633 | 8.433641 | 0.005424 | 0.152247 | Up |
| novel401_mature | GTCCTGTCGGTGTCGCCT | 18 | 189.5781 | 7.566649 | 0.008509 | 0.176002 | Up |
| novel415_mature | AGAGCTTATGAGGGAGGA | 18 | 3.35E-07 | -21.511 | 3.53E-09 | 4.62E-07 | Down |
| novel473_mature | AACAGTATAGATGAAGTAC | 19 | 0.00402 | -7.95873 | 0.013845 | 0.247327 | Down |
| novel518_mature | GCCTACAAGCAGTCGGAGC | 19 | 0.005442 | -7.52155 | 0.019476 | 0.273353 | Down |
| novel545_mature | ATCCTGGCAGTGGGACCA | 18 | 36.82664 | 5.202678 | 0.02696 | 0.3311 | Up |
| novel548_mature | AAATGGGGAACGTGAGCT | 18 | 0.007623 | -7.03535 | 0.038988 | 0.356212 | Down |
| novel558_mature | GAAGCTCCTGCGATTTGT | 18 | 55.86528 | 5.80388 | 0.01629 | 0.266756 | Up |
| novel616_mature | TGAGAATTGAATTCCATAGAT | 21 | 0.013306 | -6.23177 | 0.032607 | 0.356212 | Down |
| novel632_mature | TGGCCCTGGGAAGAGTTCA | 19 | 112.9634 | 6.819711 | 0.002928 | 0.095906 | Up |
| novel642_mature | AGACAGGTGCTGCATGGA | 18 | 0.005675 | -7.46118 | 0.022087 | 0.299318 | Down |
| novel650_mature | TCCTGGTGCTAGTAGCAC | 18 | 69.69663 | 6.123017 | 0.018896 | 0.273353 | Up |
| novel673_mature | TCAGCTGTGTATCTCTGTGCC | 21 | 466.3387 | 8.865234 | 0.004824 | 0.145818 | Up |
| novel687_mature | AGTGGGGGTAGAGCACTG | 18 | 1490.71 | 10.54178 | 0.000945 | 0.063013 | Up |
| novel689_mature | TCCTTGGGTGGAAGCTGTG | 19 | 154.7928 | 7.274195 | 0.043756 | 0.356212 | Up |
| novel693_mature | TTCACAGTCTGGGCACCT | 18 | 130.7825 | 7.031026 | 0.002642 | 0.095906 | Up |
| novel701_mature | GGAGGCAGCAGTGGGGAA | 18 | 26.57156 | 4.731811 | 0.038023 | 0.356212 | Up |
| novel703_star | CGGCGGCGGAGGCGGCGGTGG | 21 | 115.0233 | 6.845782 | 0.038744 | 0.356212 | Up |
| novel759_mature | AGGGATCACCTGGGAACT | 18 | 1570.19 | 10.61672 | 0.000362 | 0.035551 | Up |
| novel851_mature | CTTGGGTGTGCTGGTGAAGT | 20 | 103.1655 | 6.688816 | 0.007664 | 0.167332 | Up |

**Note:** TCM, traditional Chinese medicine; CAG, chronic atrophic gastritis; PDHS: Pi-wei damp-heat syndrome; PQDS: Pi-qi-deficiency syndrome.

^a^ The miRNAs marked in brown font denoted the common differential miRNAs observed both in PDHS and PQDS, compared with the healthy control.

^b^ normalized mean count value (CAG with TCM-defined PDHS, n=3) / normalized mean count value (healthy control, n=3).

^c^ the adjusted *P*-value.

**Table S7** List of the differentially expressed miRNAs in serum in PQDS

| **miRNA_id** | **Sequence** | **Length** | **FoldChange** ^a^  **F(n=4)/A(n=3)** | **log2FoldChange** | **p-value** | **q-value ^b^** | **Regulation** |
| --- | --- | --- | --- | --- | --- | --- | --- |
| hsa-let-7a-3p | CTATACAATCTACTGTCTTTC | 21 | 0.006102 | -7.35661 | 0.016244 | 0.114342 | Down |
| hsa-let-7b-3p | CTATACAACCTACTGCCTTCCC | 22 | 0.009631 | -6.69814 | 0.025724 | 0.144296 | Down |
| hsa-miR-10b-3p | ACAGATTCGATTCTAGGGGAAT | 22 | 0.001655 | -9.23936 | 0.000986 | 0.020817 | Down |
| hsa-miR-122-5p | TGGAGTGTGACAATGGTGTTTG | 22 | 51.92444 | 5.698342 | 0.006296 | 0.066177 | Up |
| hsa-miR-1226-3p | TCACCAGCCCTGTGTTCCCTAG | 22 | 0.008894 | -6.81295 | 0.034637 | 0.155434 | Down |
| hsa-miR-1275 | GTGGGGGAGAGGCTGTC | 17 | 105.9995 | 6.727914 | 0.049242 | 0.190086 | Up |
| hsa-miR-1306-5p | CCACCTCCCCTGCAAACGTCCA | 22 | 0.01205 | -6.37488 | 0.048531 |  | Down |
| hsa-miR-132-3p | TAACAGTCTACAGCCATGGTCG | 22 | 0.010169 | -6.61967 | 0.04158 | 0.175612 | Down |
| hsa-miR-142-3p | TGTAGTGTTTCCTACTTTATGGA | 23 | 0.016816 | -5.89403 | 0.017761 | 0.122622 | Down |
| hsa-miR-1468-5p | CTCCGTTTGCCTGTTTCGCTG | 21 | 282.2879 | 8.141023 | 0.012988 | 0.099209 | Up |
| hsa-miR-148a-3p | TCAGTGCACTACAGAACTTTGT | 22 | 23.45089 | 4.551571 | 0.032609 | 0.154032 | Up |
| hsa-miR-148a-5p | AAAGTTCTGAGACACTCCGACT | 22 | 0.004976 | -7.65065 | 0.004459 | 0.055984 | Down |
| hsa-miR-149-5p | TCTGGCTCCGTGTCTTCACTCCC | 23 | 0.006006 | -7.37934 | 0.003223 | 0.052593 | Down |
| hsa-miR-151a-3p | CTAGACTGAAGCTCCTTGAGG | 21 | 100.2374 | 6.647277 | 0.003703 | 0.055388 | Up |
| hsa-miR-181d-5p | AACATTCATTGTTGTCGGTGGGT | 23 | 0.01545 | -6.01625 | 0.047538 | 0.187541 | Down |
| hsa-miR-1843 | TATGGAGGTCTCTGTCTGGC | 20 | 95.74041 | 6.581056 | 0.04153 | 0.175612 | Up |
| hsa-miR-18a-3p | ACTGCCCTAAGTGCTCCTTCTGG | 23 | 0.01065 | -6.55301 | 0.028054 | 0.149424 | Down |
| hsa-miR-19a-3p | TGTGCAAATCTATGCAAAACTGA | 23 | 0.005889 | -7.40783 | 0.004522 | 0.055984 | Down |
| hsa-miR-206 | TGGAATGTAAGGAAGTGTGTGG | 22 | 36.78798 | 5.201163 | 0.031554 | 0.153079 | Up |
| hsa-miR-25-5p | AGGCGGAGACTTGGGCAATTG | 21 | 9313147 | 23.15084 | 5.78E-11 | 2.59E-09 | Up |
| hsa-miR-26b-3p | CCTGTTCTCCATTACTTGGCT | 21 | 0.015539 | -6.00794 | 0.04404 | 0.178091 | Down |
| hsa-miR-29b-3p | TAGCACCATTTGAAATCAGTGTT | 23 | 0.009452 | -6.72517 | 0.039298 | 0.172048 | Down |
| hsa-miR-29c-3p | TAGCACCATTTGAAATCGGTTA | 22 | 0.0167 | -5.90404 | 0.031068 | 0.152788 | Down |
| hsa-miR-3158-3p | AAGGGCTTCCTCTCTGCAGGAC | 22 | 449.1616 | 8.811091 | 0.009053 | 0.073865 | Up |
| hsa-miR-3173-5p | TGCCCTGCCTGTTTTCTCCTTT | 22 | 0.017217 | -5.85998 | 0.046504 |  | Down |
| hsa-miR-3184-5p | TGAGGGGCCTCAGACCGAGCTTTT | 24 | 32.02877 | 5.001297 | 0.043201 | 0.178091 | Up |
| hsa-miR-32-5p | TATTGCACATTACTAAGTTGCA | 22 | 0.011158 | -6.48572 | 0.044695 |  | Down |
| hsa-miR-329-3p | AACACACCTGGTTAACCTCTTT | 22 | 0.005233 | -7.5782 | 0.003593 | 0.055388 | Down |
| hsa-miR-331-3p | GCCCCTGGGCCTATCCTAGAA | 21 | 0.021343 | -5.55008 | 0.030483 | 0.151992 | Down |
| hsa-miR-331-5p | CTAGGTATGGTCCCAGGGATCC | 22 | 0.005994 | -7.38226 | 0.022109 | 0.139628 | Down |
| hsa-miR-3613-3p | ACAAAAAAAAAAGCCCAACCCTTC | 24 | 0.016694 | -5.90455 | 0.046197 |  | Down |
| hsa-miR-369-3p | AATAATACATGGTTGATCTTT | 21 | 0.016293 | -5.93964 | 0.046545 |  | Down |
| hsa-miR-375-3p | TTTGTTCGTTCGGCTCGCGTGA | 22 | 64.23087 | 6.005195 | 0.006388 | 0.066177 | Up |
| hsa-miR-3940-3p | CAGCCCGGATCCCAGCCCACTT | 22 | 0.017612 | -5.82732 | 0.047775 |  | Down |
| hsa-miR-409-5p | AGGTTACCCGAGCAACTTTGCAT | 23 | 0.00258 | -8.59831 | 0.00387 | 0.055573 | Down |
| hsa-miR-421 | ATCAACAGACATTAATTGGGCGC | 23 | 0.02259 | -5.46819 | 0.03307 | 0.154181 | Down |
| hsa-miR-532-5p | CATGCCTTGAGTGTAGGACCGT | 22 | 32.74121 | 5.033036 | 0.019551 | 0.132434 | Up |
| hsa-miR-654-5p | TGGTGGGCCGCAGAACATGTGC | 22 | 69.93383 | 6.127919 | 0.029135 | 0.149424 | Up |
| hsa-miR-6721-5p | TGGGCAGGGGCTTATTGTAGGAG | 23 | 92.62224 | 6.533287 | 0.044151 | 0.178091 | Up |
| hsa-miR-6780a-5p | TTGGGAGGGAAGACAGCTGGAGA | 23 | 7631790 | 22.86359 | 8.05E-11 | 2.80E-09 | Up |
| hsa-miR-6842-3p | TTGGCTGGTCTCTGCTCCGCAG | 22 | 13636740 | 23.701 | 1.65E-11 | 1.18E-09 | Up |
| hsa-miR-7706 | TGAAGCGCCTGTGCTCTGCCGAGA | 24 | 94.93745 | 6.568905 | 0.035444 | 0.15709 | Up |
| hsa-miR-95-3p | TTCAACGGGTATTTATTGAGCA | 22 | 108.7757 | 6.765213 | 0.033859 | 0.155434 | Up |
| novel101_mature | TCATGGTTTGCCTGGGACTA | 20 | 212.6714 | 7.732482 | 0.023746 | 0.139628 | Up |
| novel108_mature | ATCCTGGGGCTGGAGGAGG | 19 | 466.7207 | 8.866416 | 0.005393 | 0.062455 | Up |
| novel10_mature | TCCTGTTTCCTGGCACCC | 18 | 163.1163 | 7.349757 | 0.032295 | 0.154032 | Up |
| novel114_mature | TCCTGTGCTGAGCTGCCCT | 19 | 36.9255 | 5.206546 | 0.029696 | 0.150151 | Up |
| novel130_mature | TAGCAGTGAGACTGTGGGG | 19 | 317.3324 | 8.309851 | 0.006518 | 0.066177 | Up |
| novel132_mature | TGAGGGGTAGAGAGAGAGA | 19 | 106.7756 | 6.738439 | 0.02309 | 0.139628 | Up |
| novel133_mature | CAGAGGAAGGTGGGGATG | 18 | 536.2668 | 9.066807 | 0.000876 | 0.020817 | Up |
| novel144_mature | TGTGAGCACTCGAAGATA | 18 | 52.2421 | 5.707141 | 0.027403 | 0.149054 | Up |
| novel154_mature | CGACTGGGGGACTGCCTA | 18 | 437.3364 | 8.7726 | 0.012117 | 0.094565 | Up |
| novel161_mature | ACTTGGGACTGGCTCAGAG | 19 | 336.5119 | 8.394514 | 0.015892 | 0.114107 | Up |
| novel221_mature | CTAGACTGGAGTTCCTTGAGG | 21 | 987.636 | 9.947836 | 0.001322 | 0.026373 | Up |
| novel249_mature | TGAGGGGCAGGGAGTGAGA | 19 | 258.1022 | 8.011798 | 0.011133 | 0.088819 | Up |
| novel267_mature | AGAATTGTAGTCTATGGA | 18 | 943.5036 | 9.881884 | 0.004876 | 0.058345 | Up |
| novel278_mature | GCGGATGTGGCTCAGAGG | 18 | 39184022 | 25.22376 | 3.01E-12 | 1.08E-09 | Up |
| novel322_mature | GGGGACGACGGCGGGGGC | 18 | 562.5239 | 9.135771 | 0.004266 | 0.055984 | Up |
| novel33_mature | TCCGCTGCAGCCCCTCGACGT | 21 | 15311824 | 23.86814 | 1.10E-11 | 1.18E-09 | Up |
| novel349_mature | TTGGGAGTGGGCGGGCGG | 18 | 125.1111 | 6.967066 | 0.048969 | 0.190086 | Up |
| novel365_mature | TGAGCACCCCAGGACCTGCGCT | 22 | 0.006263 | -7.31897 | 0.024114 | 0.139628 | Down |
| novel374_mature | TGGGGGGCAGAGGGCGAG | 18 | 150.132 | 7.230088 | 0.034246 | 0.155434 | Up |
| novel379_mature | TGCCCTGTGGCTCCACCA | 18 | 17025666 | 24.02121 | 1.37E-11 | 1.18E-09 | Up |
| novel401_mature | GTCCTGTCGGTGTCGCCT | 18 | 728.0614 | 9.507916 | 0.006636 | 0.066177 | Up |
| novel425_mature | TCAGACGTCTCGAGGCTCGCG | 21 | 12710736 | 23.59954 | 3.04E-11 | 1.56E-09 | Up |
| novel429_mature | GGTGGCTGTAGCTCAGTG | 18 | 5223461 | 22.31657 | 2.77E-10 | 7.64E-09 | Up |
| novel43_mature | TGGGGCGTCGCCAAGTGG | 18 | 99.42477 | 6.635533 | 0.026877 | 0.148445 | Up |
| novel441_mature | TGAAGGCTCGGGACTGTG | 18 | 7835443 | 22.90158 | 8.09E-11 | 2.80E-09 | Up |
| novel475_mature | GACAGCAGGAAGGTGGCT | 18 | 246.8047 | 7.947226 | 0.007846 | 0.068701 | Up |
| novel476_mature | CGGGGGCCCTGGGAAGAG | 18 | 946.8747 | 9.88703 | 0.001467 | 0.027715 | Up |
| novel47_mature | TCCCTGTCCTCCAGGAGCTCA | 21 | 0.00935 | -6.74078 | 0.013924 | 0.10286 | Down |
| novel481_mature | TCCTGTGCTGAGCTGACCCG | 20 | 47.93664 | 5.583057 | 0.028525 | 0.149424 | Up |
| novel502_mature | AGACAGCGTCAGGTGTGG | 18 | 158.4716 | 7.308081 | 0.024005 | 0.139628 | Up |
| novel503_mature | TGAGGTACAGAGAGCGAGA | 19 | 149.6402 | 7.225354 | 0.020941 | 0.139216 | Up |
| novel518_mature | GCCTACAAGCAGTCGGAGC | 19 | 0.00572 | -7.44976 | 0.023595 | 0.139628 | Down |
| novel550_mature | GGTGATAAAGGAGGTGGGA | 19 | 53.88858 | 5.751908 | 0.023052 | 0.139628 | Up |
| novel557_mature | GATGAGATGTGGGTAGGGG | 19 | 338.7121 | 8.403916 | 0.007335 | 0.066586 | Up |
| novel572_mature | GCTGACTTGATTTTGCTTTT | 20 | 683.7394 | 9.417303 | 0.007057 | 0.066586 | Up |
| novel580_mature | GGCTGACTTGATTTTGCTTTT | 21 | 7776428 | 22.89068 | 8.57E-11 | 2.80E-09 | Up |
| novel596_mature | GTGTGTGTCGTGATAGGT | 18 | 12483359 | 23.5735 | 2.84E-11 | 1.56E-09 | Up |
| novel5_mature | ACACGTGAAACCCTGTCT | 18 | 61.45891 | 5.94155 | 0.043611 | 0.178091 | Up |
| novel632_mature | TGGCCCTGGGAAGAGTTCA | 19 | 173.3796 | 7.43779 | 0.014039 | 0.10286 | Up |
| novel641_mature | TGACGAGTGGTGGACGGGC | 19 | 175.6139 | 7.456263 | 0.029136 | 0.149424 | Up |
| novel668_mature | GGGACGTGTAGCTGGAAAA | 19 | 792.9693 | 9.631121 | 0.005993 | 0.066177 | Up |
| novel673_mature | TCAGCTGTGTATCTCTGTGCC | 21 | 13993919 | 23.7383 | 1.55E-11 | 1.18E-09 | Up |
| novel689_mature | TCCTTGGGTGGAAGCTGTG | 19 | 911.1163 | 9.831491 | 0.002388 | 0.040826 | Up |
| novel705_mature | TGAGAGCAGAGAGCGAGAC | 19 | 125.7384 | 6.974282 | 0.040298 | 0.1743 | Up |
| novel709_mature | AGTTTGGCTGGGGCGGTC | 18 | 106.1816 | 6.73039 | 0.022353 | 0.139628 | Up |
| novel737_mature | AGAGGGACTGTGTGATGT | 18 | 449.3445 | 8.811678 | 0.001831 | 0.03287 | Up |
| novel756_mature | CTGGGGTCGGGGGACAGC | 18 | 5372945 | 22.35728 | 2.70E-10 | 7.64E-09 | Up |
| novel778_mature | GTGGAATTCCGAGTGTGGA | 19 | 1014.397 | 9.986406 | 0.004424 | 0.055984 | Up |
| novel803_mature | TGCTGTGGCTGGGTTGTG | 18 | 679.0952 | 9.40747 | 0.007213 | 0.066586 | Up |
| novel817_mature | ATGCCCTGGGGCGGGCTG | 18 | 624.3495 | 9.28621 | 0.008166 | 0.0698 | Up |
| novel82_mature | TGAGGGGCAGAGAGCAGGA | 19 | 59.80024 | 5.902079 | 0.046607 | 0.18591 | Up |
| novel851_mature | CTTGGGTGTGCTGGTGAAGT | 20 | 286.4355 | 8.162066 | 0.000529 | 0.013567 | Up |
| novel862_mature | TGCAGATCTTGGTGGTAGC | 19 | 309.7569 | 8.274993 | 0.000965 | 0.020817 | Up |
| novel866_mature | CCAGATCTCAGATTAAGC | 18 | 387.4077 | 8.597709 | 0.008728 | 0.072865 | Up |
| novel889_mature | TCAGCTTTGGATGTTAGG | 18 | 194.8849 | 7.606479 | 0.025686 | 0.144296 | Up |
| novel89_mature | TGGAGGGTGTTTCTGAGACAGTG | 23 | 666.1635 | 9.379732 | 0.007419 | 0.066586 | Up |

**Note:** TCM, traditional Chinese medicine; CAG, chronic atrophic gastritis; PQDS: Pi-qi-deficiency sydrome.

^a^ normalized mean count value (CAG with TCM-defined PQDS, n=4) / normalized mean count value (healthy control, n=3);

^b^ the adjusted *P*-value.

**Table S8** The detailed interaction relationships among the PDHS-specific genes

| **node1** | **node2** | **Homology ^a^** | **Co-expression ^b^** | **Experimentally determined interaction ^c^** | **Database annotated ^d^** | **Automated textmining ^e^** | **Combined score ^f^** |
| --- | --- | --- | --- | --- | --- | --- | --- |
| CEACAM8 | CEACAM6 | 0.977 | 0.323 | 0.87 | 0.9 | 0.752 | 0.99 |
| MPO | CTSG | 0 | 0.612 | 0.061 | 0.9 | 0.737 | 0.989 |
| AZU1 | MPO | 0 | 0.673 | 0.061 | 0.9 | 0.594 | 0.985 |
| BPI | MPO | 0 | 0.54 | 0 | 0.9 | 0.669 | 0.983 |
| BPI | AZU1 | 0 | 0.32 | 0 | 0.9 | 0.759 | 0.982 |
| BPI | CTSG | 0 | 0.221 | 0 | 0.9 | 0.7 | 0.974 |
| CAMP | BPI | 0 | 0.327 | 0 | 0.9 | 0.647 | 0.974 |
| GNG10 | GNAI1 | 0 | 0.061 | 0.659 | 0.9 | 0.297 | 0.974 |
| DEFA1 | DEFA3 | 0.983 | 0.771 | 0.87 | 0 | 0.74 | 0.969 |
| AZU1 | CTSG | 0.794 | 0.559 | 0 | 0.9 | 0.731 | 0.964 |
| GNAI1 | GNAO1 | 0.974 | 0.085 | 0.561 | 0.9 | 0.598 | 0.957 |
| NRCAM | SCN8A | 0 | 0.118 | 0 | 0.9 | 0.557 | 0.957 |
| GNG10 | GNAO1 | 0 | 0.049 | 0.31 | 0.9 | 0.426 | 0.957 |
| DEFA1B | DEFA3 | 0.983 | 0.771 | 0.8 | 0 | 0.736 | 0.952 |
| GATM | ARG1 | 0 | 0 | 0 | 0.9 | 0.495 | 0.948 |
| IGFBP3 | CTSG | 0 | 0 | 0.379 | 0.9 | 0.186 | 0.945 |
| HEY1 | GATA6 | 0 | 0 | 0.156 | 0.9 | 0.373 | 0.942 |
| ARG1 | MPO | 0 | 0.102 | 0 | 0.9 | 0.39 | 0.94 |
| BPI | OLFM4 | 0 | 0.114 | 0 | 0.9 | 0.285 | 0.931 |
| ARG1 | ORM1 | 0 | 0.22 | 0 | 0.9 | 0.1 | 0.923 |
| ARG1 | BPI | 0 | 0.146 | 0 | 0.9 | 0.163 | 0.922 |
| NRCAM | SCN3A | 0 | 0.116 | 0 | 0.9 | 0.185 | 0.921 |
| ARG1 | CTSG | 0 | 0.091 | 0 | 0.9 | 0.197 | 0.92 |
| ABCA13 | CEACAM8 | 0 | 0.076 | 0 | 0.9 | 0.199 | 0.919 |
| ARG1 | CAMP | 0 | 0.137 | 0 | 0.9 | 0.121 | 0.917 |
| ABCA13 | CEACAM6 | 0 | 0 | 0 | 0.9 | 0.173 | 0.913 |
| OLR1 | CEACAM8 | 0 | 0.077 | 0 | 0.9 | 0.138 | 0.913 |
| CAMP | OLFM4 | 0 | 0.115 | 0 | 0.9 | 0.086 | 0.912 |
| CAMP | ORM1 | 0 | 0.101 | 0 | 0.9 | 0.097 | 0.911 |
| DEFA1 | DEFA1B | 0.983 | 0.522 | 0.82 | 0 | 0 | 0.91 |
| ACOT4 | BAAT | 0.903 | 0 | 0 | 0.9 | 0.637 | 0.909 |
| DNAJC6 | VAMP7 | 0 | 0.092 | 0 | 0.9 | 0.056 | 0.906 |
| ORM1 | OLFM4 | 0 | 0 | 0 | 0.9 | 0.105 | 0.906 |
| ARG1 | AZU1 | 0 | 0 | 0 | 0.9 | 0.084 | 0.904 |
| BPI | ORM1 | 0 | 0.069 | 0 | 0.9 | 0.043 | 0.903 |
| TARM1 | OLR1 | 0 | 0.061 | 0 | 0.9 | 0 | 0.902 |
| TARM1 | CEACAM8 | 0 | 0.065 | 0 | 0.9 | 0 | 0.902 |
| ARG1 | OLFM4 | 0 | 0 | 0 | 0.9 | 0.056 | 0.901 |
| GPR20 | GNG10 | 0 | 0 | 0 | 0.9 | 0 | 0.9 |
| BPI | CEACAM8 | 0 | 0.727 | 0 | 0 | 0.329 | 0.808 |
| CAMP | CTSG | 0 | 0.194 | 0.379 | 0 | 0.635 | 0.801 |
| CHN1 | ROBO3 | 0 | 0.061 | 0 | 0 | 0.757 | 0.762 |
| DNAJC6 | CACHD1 | 0 | 0 | 0 | 0 | 0.74 | 0.74 |
| MTCL1 | EML6 | 0 | 0.063 | 0 | 0 | 0.689 | 0.696 |
| SLX1B | RMI2 | 0 | 0.058 | 0 | 0 | 0.69 | 0.696 |
| CEACAM8 | MPO | 0 | 0.316 | 0 | 0 | 0.57 | 0.693 |
| CAMP | MPO | 0 | 0.252 | 0 | 0 | 0.577 | 0.67 |
| CAMP | AZU1 | 0 | 0.137 | 0 | 0 | 0.63 | 0.667 |
| CAMP | CEACAM8 | 0 | 0.56 | 0 | 0 | 0.252 | 0.656 |
| NEFL | TRIM2 | 0 | 0.153 | 0.299 | 0 | 0.462 | 0.653 |
| REG4 | OLFM4 | 0 | 0.14 | 0 | 0 | 0.576 | 0.62 |
| DEFA1 | CAMP | 0 | 0.314 | 0 | 0 | 0.441 | 0.6 |
| TNIP3 | MLF1 | 0 | 0.063 | 0 | 0 | 0.59 | 0.599 |
| SCN8A | GNAO1 | 0 | 0.156 | 0.379 | 0 | 0.292 | 0.596 |
| DEFA1B | CAMP | 0 | 0.334 | 0 | 0 | 0.418 | 0.595 |
| DEFA3 | CTSG | 0 | 0.302 | 0 | 0 | 0.429 | 0.584 |
| IFNG | MPO | 0 | 0 | 0 | 0 | 0.581 | 0.581 |
| FBLN2 | CD248 | 0.552 | 0.528 | 0 | 0 | 0.266 | 0.578 |
| DEFA3 | BPI | 0 | 0.299 | 0 | 0 | 0.418 | 0.574 |
| DEFA1 | CTSG | 0 | 0.312 | 0 | 0 | 0.399 | 0.569 |
| DEFA1 | MPO | 0 | 0.306 | 0 | 0 | 0.377 | 0.549 |
| DEFA3 | CAMP | 0 | 0.35 | 0 | 0 | 0.314 | 0.535 |
| LRRN3 | NRCAM | 0 | 0.098 | 0.137 | 0 | 0.448 | 0.532 |
| ARG1 | CEACAM8 | 0 | 0.152 | 0 | 0 | 0.47 | 0.531 |
| ARG1 | IFNG | 0 | 0 | 0 | 0 | 0.529 | 0.529 |
| DEFA1 | BPI | 0 | 0.238 | 0 | 0 | 0.399 | 0.522 |
| MLF1 | ADAM23 | 0 | 0 | 0 | 0 | 0.515 | 0.515 |
| CHN1 | ARHGEF4 | 0 | 0.107 | 0.315 | 0 | 0.269 | 0.514 |
| RCAN2 | TMEM220 | 0 | 0 | 0 | 0 | 0.508 | 0.508 |
| DEFA3 | MPO | 0 | 0.333 | 0 | 0 | 0.291 | 0.506 |
| REG4 | GATA6 | 0 | 0 | 0.08 | 0 | 0.484 | 0.505 |
| CHN1 | GPM6A | 0 | 0.158 | 0 | 0 | 0.436 | 0.505 |
| SHANK1 | N4BP3 | 0 | 0 | 0 | 0 | 0.502 | 0.502 |
| DEFA1B | BPI | 0 | 0.214 | 0 | 0 | 0.389 | 0.499 |
| CEACAM8 | CTSG | 0 | 0.284 | 0 | 0 | 0.327 | 0.497 |
| DEFA1B | CTSG | 0 | 0.229 | 0 | 0 | 0.374 | 0.496 |
| BAAT | CYP27A1 | 0 | 0 | 0 | 0 | 0.491 | 0.491 |
| HEY1 | LDB2 | 0 | 0.061 | 0 | 0 | 0.477 | 0.488 |
| ZNF610 | SORCS3 | 0 | 0 | 0 | 0 | 0.474 | 0.474 |
| DEFA1B | MPO | 0 | 0.214 | 0 | 0 | 0.358 | 0.473 |
| CEACAM8 | AZU1 | 0 | 0.322 | 0 | 0 | 0.25 | 0.469 |
| SLC24A2 | GPM6A | 0 | 0.462 | 0 | 0 | 0.05 | 0.468 |
| IGFBP2 | IFNG | 0 | 0 | 0 | 0 | 0.453 | 0.453 |
| ESPN | KCNMB4 | 0 | 0 | 0 | 0 | 0.45 | 0.45 |
| DEFA3 | AZU1 | 0 | 0.115 | 0 | 0 | 0.404 | 0.449 |
| ZNF285 | LRRN3 | 0 | 0 | 0.053 | 0 | 0.442 | 0.448 |
| EMILIN1 | CD248 | 0 | 0.448 | 0 | 0 | 0 | 0.448 |
| IGLL5 | CR2 | 0 | 0.088 | 0 | 0 | 0.416 | 0.444 |
| SHANK1 | GNAO1 | 0 | 0.145 | 0.073 | 0 | 0.346 | 0.436 |
| DEFA3 | OLFM4 | 0 | 0.096 | 0 | 0 | 0.4 | 0.434 |
| DEFA1 | AZU1 | 0 | 0.098 | 0 | 0 | 0.398 | 0.433 |
| HEY1 | FOXJ1 | 0 | 0.061 | 0.058 | 0 | 0.407 | 0.43 |
| ABCA13 | TRIM2 | 0 | 0.055 | 0.054 | 0 | 0.413 | 0.429 |
| OLFM4 | CEACAM6 | 0 | 0.16 | 0 | 0 | 0.347 | 0.428 |
| CEACAM8 | OLFM4 | 0 | 0.185 | 0 | 0 | 0.32 | 0.422 |
| AQP1 | IFNG | 0 | 0 | 0 | 0 | 0.417 | 0.417 |
| SCN8A | SCN3A | 0.976 | 0.107 | 0 | 0.36 | 0.897 | 0.416 |
| ADAMTSL5 | CAMP | 0 | 0 | 0 | 0 | 0.417 | 0.416 |
| FOXJ1 | GATA6 | 0 | 0 | 0.153 | 0 | 0.335 | 0.413 |
| XKRX | SLC24A2 | 0 | 0 | 0 | 0 | 0.404 | 0.404 |
| LRFN2 | SHANK1 | 0 | 0.158 | 0.105 | 0 | 0.267 | 0.4 |

**Note:** PDHS: Pi-wei damp-heat syndrome.

**^a^** Cooccurrence, Gene families whose occurrence patterns across genomes show similarities;

**^b^** Co-expression, Proteins whose genes are observed to be correlated in expression, across a large number of experiments;

**^c^** Experiments, Co-purification, co-crystallization, Yeast2Hybrid, Genetic Interactions, etc ... as imported from primary sources;

**^d^** Database, Known metabolic pathways, protein complexes, signal transduction pathways, etc ... from curated databases;

**^e^** Textmining, Automated, unsupervised textmining - searching for proteins that are frequently mentioned together;

^f^ The value determines the thickness of edge between nudes in a created interaction network cartoon, indicating the interaction strength of all the support data.

**Table S9** The detailed interaction relationships among the PQDS-specific genes

| **node1** | **node2** | **Homology ^a^** | **Co-expression ^b^** | **Experimentally determined interaction ^c^** | **Database annotated ^d^** | **Automated textmining ^e^** | **Combined score ^f^** |
| --- | --- | --- | --- | --- | --- | --- | --- |
| COL4A2 | COL10A1 | 0.703 | 0.061 | 0.55 | 0.9 | 0.217 | 0.961 |
| PROS1 | PROCR | 0 | 0.06 | 0 | 0.9 | 0.547 | 0.953 |
| NEK2 | ANAPC1 | 0 | 0.109 | 0.369 | 0.9 | 0.135 | 0.944 |
| COL4A2 | PLOD2 | 0 | 0.158 | 0.185 | 0.9 | 0.278 | 0.943 |
| EPS8 | EGF | 0 | 0 | 0 | 0.9 | 0.429 | 0.94 |
| PF4 | EGF | 0 | 0 | 0 | 0.9 | 0.423 | 0.939 |
| COL4A2 | COL5A3 | 0.658 | 0.12 | 0 | 0.9 | 0.523 | 0.933 |
| HLA-DRB5 | HLA-DQB1 | 0.963 | 0.311 | 0 | 0.9 | 0.769 | 0.93 |
| MMRN1 | PF4 | 0 | 0.079 | 0 | 0.9 | 0.291 | 0.929 |
| EGF | A1BG | 0 | 0 | 0 | 0.9 | 0.322 | 0.929 |
| PF4 | CXCL6 | 0.869 | 0.055 | 0.221 | 0.9 | 0.805 | 0.928 |
| COL13A1 | COL4A2 | 0.694 | 0.139 | 0 | 0.9 | 0.32 | 0.926 |
| PF4 | PROCR | 0 | 0.062 | 0 | 0.9 | 0.275 | 0.926 |
| REEP1 | OR2W3 | 0 | 0 | 0 | 0.9 | 0.285 | 0.925 |
| COL4A2 | COL26A1 | 0 | 0 | 0 | 0.9 | 0.077 | 0.924 |
| COL4A4 | COL5A3 | 0.679 | 0 | 0 | 0.9 | 0.428 | 0.922 |
| COL13A1 | COL5A3 | 0.712 | 0.076 | 0 | 0.9 | 0.371 | 0.921 |
| COL4A4 | PLOD2 | 0 | 0.061 | 0.185 | 0.9 | 0.085 | 0.92 |
| COL13A1 | COL4A4 | 0.722 | 0.061 | 0 | 0.9 | 0.335 | 0.918 |
| COL13A1 | PLOD2 | 0 | 0.069 | 0.093 | 0.9 | 0.151 | 0.918 |
| CYP2E1 | AKR1C1 | 0 | 0.062 | 0 | 0.9 | 0.187 | 0.917 |
| COL10A1 | COL5A3 | 0.743 | 0 | 0 | 0.9 | 0.29 | 0.916 |
| COL4A4 | COL10A1 | 0.737 | 0.061 | 0 | 0.9 | 0.169 | 0.915 |
| PROS1 | EGF | 0.555 | 0 | 0 | 0.9 | 0.348 | 0.914 |
| COL4A4 | COL26A1 | 0.605 | 0 | 0 | 0.9 | 0.216 | 0.914 |
| COL4A4 | COL4A2 | 0.878 | 0.061 | 0 | 0.9 | 0.743 | 0.914 |
| COL26A1 | COL5A3 | 0.589 | 0 | 0 | 0.9 | 0.161 | 0.914 |
| COL13A1 | COL10A1 | 0.705 | 0 | 0 | 0.9 | 0.088 | 0.913 |
| PROS1 | A1BG | 0 | 0 | 0 | 0.9 | 0.164 | 0.912 |
| MMRN1 | EGF | 0 | 0 | 0 | 0.9 | 0.145 | 0.91 |
| PLOD2 | COL5A3 | 0 | 0.061 | 0.093 | 0.9 | 0.067 | 0.909 |
| MMRN1 | PROS1 | 0 | 0 | 0 | 0.9 | 0.132 | 0.909 |
| COL10A1 | COL26A1 | 0.596 | 0 | 0 | 0.9 | 0 | 0.908 |
| AREG | EGF | 0 | 0.061 | 0 | 0 | 0.905 | 0.908 |
| PROS1 | PF4 | 0 | 0 | 0 | 0.9 | 0.111 | 0.907 |
| COL10A1 | PLOD2 | 0 | 0 | 0 | 0.9 | 0.11 | 0.907 |
| HIP1 | EGF | 0 | 0 | 0.053 | 0.9 | 0.073 | 0.904 |
| RPP25 | RPS16 | 0 | 0.088 | 0 | 0.9 | 0 | 0.904 |
| PF4 | A1BG | 0 | 0 | 0 | 0.9 | 0.078 | 0.903 |
| COL13A1 | COL26A1 | 0.595 | 0.061 | 0 | 0.9 | 0 | 0.902 |
| ADORA3 | CXCL6 | 0 | 0 | 0 | 0.9 | 0.064 | 0.902 |
| TAS2R31 | ADORA3 | 0 | 0 | 0 | 0.9 | 0 | 0.9 |
| ANAPC1 | SH3RF1 | 0 | 0 | 0 | 0.9 | 0 | 0.9 |
| COL26A1 | PLOD2 | 0 | 0 | 0 | 0.9 | 0.043 | 0.9 |
| MMRN1 | A1BG | 0 | 0 | 0 | 0.9 | 0.049 | 0.9 |
| REEP1 | OR6N2 | 0 | 0 | 0 | 0.9 | 0 | 0.9 |
| ADORA3 | PF4 | 0 | 0 | 0 | 0.9 | 0 | 0.9 |
| PTPRB | PGRMC1 | 0 | 0 | 0 | 0.9 | 0.05 | 0.9 |
| REEP1 | OR1J2 | 0 | 0 | 0 | 0.9 | 0 | 0.9 |
| TAS2R31 | CXCL6 | 0 | 0 | 0 | 0.9 | 0 | 0.9 |
| TAS2R31 | PF4 | 0 | 0 | 0 | 0.9 | 0 | 0.9 |
| MKI67 | NEK2 | 0 | 0.813 | 0 | 0 | 0.348 | 0.873 |
| FOS | EGF | 0 | 0 | 0 | 0 | 0.862 | 0.863 |
| MMP1 | FOS | 0 | 0 | 0.379 | 0 | 0.723 | 0.821 |
| MMP1 | EGF | 0 | 0 | 0 | 0 | 0.77 | 0.771 |
| FOS | DCSTAMP | 0 | 0 | 0 | 0 | 0.769 | 0.769 |
| PDZK1 | PDZK1IP1 | 0 | 0.063 | 0.379 | 0 | 0.627 | 0.764 |
| FKBP1B | AKAP6 | 0 | 0 | 0 | 0 | 0.743 | 0.743 |
| MYRFL | PTPRB | 0 | 0.062 | 0 | 0 | 0.731 | 0.737 |
| EGF | ABCG2 | 0 | 0 | 0 | 0 | 0.724 | 0.724 |
| CSF2RA | CD99 | 0 | 0 | 0 | 0 | 0.71 | 0.71 |
| SLC47A1 | ABCG2 | 0 | 0 | 0.051 | 0 | 0.671 | 0.675 |
| RNF222 | ANAPC1 | 0 | 0 | 0 | 0.54 | 0.284 | 0.656 |
| CYP2E1 | PLA2G4B | 0 | 0 | 0 | 0.65 | 0 | 0.65 |
| PLA2G4C | CYP2E1 | 0 | 0 | 0 | 0.65 | 0 | 0.65 |
| FOS | PVALB | 0 | 0 | 0.104 | 0 | 0.619 | 0.644 |
| NRGN | PVALB | 0 | 0.262 | 0.064 | 0 | 0.525 | 0.643 |
| CSF2RA | EGF | 0 | 0 | 0 | 0.6 | 0.127 | 0.636 |
| CALD1 | COL4A2 | 0 | 0.35 | 0 | 0 | 0.446 | 0.625 |
| AKAP17A | PGRMC1 | 0 | 0 | 0 | 0 | 0.625 | 0.625 |
| SOX5 | COL10A1 | 0 | 0 | 0 | 0 | 0.612 | 0.612 |
| SPP1 | ABCG2 | 0 | 0.059 | 0 | 0 | 0.559 | 0.567 |
| KRT17 | EGF | 0 | 0 | 0 | 0 | 0.56 | 0.56 |
| ALK | EGF | 0 | 0.05 | 0.05 | 0 | 0.552 | 0.56 |
| MUC1 | EGF | 0 | 0 | 0 | 0 | 0.559 | 0.559 |
| SPP1 | EGF | 0 | 0 | 0 | 0 | 0.559 | 0.559 |
| CCL23 | CXCL6 | 0 | 0.05 | 0 | 0 | 0.548 | 0.552 |
| MKI67 | EGF | 0 | 0 | 0 | 0 | 0.552 | 0.552 |
| PDZK1 | ABCG2 | 0 | 0.061 | 0.056 | 0 | 0.516 | 0.533 |
| SPP1 | MMP1 | 0 | 0.049 | 0 | 0 | 0.516 | 0.52 |
| MMP1 | PF4 | 0 | 0 | 0 | 0 | 0.519 | 0.519 |
| SPP1 | COL10A1 | 0 | 0 | 0 | 0 | 0.513 | 0.513 |
| TGFB1I1 | FOS | 0 | 0.05 | 0 | 0 | 0.509 | 0.513 |
| CCL23 | HBG2 | 0 | 0 | 0 | 0 | 0.508 | 0.508 |
| TBC1D3B | RAB6C | 0 | 0.062 | 0.129 | 0 | 0.428 | 0.492 |
| JAG2 | EGF | 0 | 0 | 0 | 0 | 0.488 | 0.488 |
| NRXN1 | PVALB | 0 | 0.157 | 0 | 0 | 0.404 | 0.476 |
| SPP1 | FOS | 0 | 0 | 0 | 0 | 0.473 | 0.473 |
| SPP1 | HOXA9 | 0 | 0 | 0 | 0 | 0.466 | 0.467 |
| REEP1 | ANO2 | 0 | 0.055 | 0 | 0 | 0.458 | 0.466 |
| PODXL | TRPC6 | 0 | 0 | 0 | 0 | 0.454 | 0.454 |
| ARSJ | GPC1 | 0 | 0.076 | 0.093 | 0 | 0.398 | 0.451 |
| TMEM171 | PDZK1 | 0 | 0 | 0 | 0 | 0.448 | 0.448 |
| FHL2 | NFIB | 0 | 0 | 0 | 0 | 0.443 | 0.443 |
| EPS8 | PVRL2 | 0 | 0 | 0 | 0 | 0.426 | 0.426 |
| MUC1 | KRT17 | 0 | 0.112 | 0 | 0 | 0.376 | 0.422 |
| COL4A4 | TRPC6 | 0 | 0 | 0 | 0 | 0.421 | 0.42 |
| MUC1 | SPP1 | 0 | 0 | 0 | 0 | 0.42 | 0.42 |
| EGF | GPC1 | 0 | 0 | 0 | 0 | 0.42 | 0.42 |
| ALK | CALD1 | 0 | 0 | 0 | 0 | 0.416 | 0.416 |
| IL17RE | RORC | 0 | 0 | 0 | 0 | 0.417 | 0.416 |
| HLA-DRB5 | AKAP17A | 0 | 0 | 0 | 0 | 0.415 | 0.414 |
| NRXN1 | SEMA5A | 0 | 0.065 | 0 | 0 | 0.398 | 0.413 |
| CCL23 | PF4 | 0 | 0.085 | 0 | 0 | 0.38 | 0.409 |
| EGF | WNT10A | 0 | 0.054 | 0.126 | 0 | 0.339 | 0.405 |
| ENSG00000214338 | DNAJB5 | 0 | 0 | 0 | 0 | 0.402 | 0.402 |
| GPC1 | WNT10A | 0 | 0 | 0.184 | 0 | 0.297 | 0.402 |
| ENSG00000214338 | SASH1 | 0 | 0.079 | 0 | 0 | 0.377 | 0.401 |

**Note:** PQDS: Pi-qi-deficiency syndrome.

**^a^** Cooccurrence, Gene families whose occurrence patterns across genomes show similarities;

**^b^** Co-expression, Proteins whose genes are observed to be correlated in expression, across a large number of experiments;

**^c^** Experiments, Co-purification, co-crystallization, Yeast2Hybrid, Genetic Interactions, etc ... as imported from primary sources;

**^d^** Database, Known metabolic pathways, protein complexes, signal transduction pathways, etc ... from curated databases;

**^e^** Textmining, Automated, unsupervised textmining - searching for proteins that are frequently mentioned together;

^f^ The value determines the thickness of edge between nudes in a created interaction network cartoon, indicating the interaction strength of all the support data.

**Table S10** The experimental evidences supporting the PDHS-specific miRNA-gene interaction pairs

| **geneId** | **geneName ^a^** | **Mirna ^a^** | **species** | **cell_line** | **tissue** | **category** | **method** | **positive_negative** | **direct_indirect** | **up_down** | **condition** |
| --- | --- | --- | --- | --- | --- | --- | --- | --- | --- | --- | --- |
| ENSG00000172915 | NBEA | hsa-miR-124-5p | *H. sapiens* | BETA Cells | Pancreas | Normal/Primary | HITS-CLIP | POSITIVE | DIRECT | DOWN | NA |
| ENSG00000168502 | MTCL1 | hsa-miR-452-5p | *H. sapiens* | BETA Cells | Pancreas | Normal/Primary | HITS-CLIP | POSITIVE | DIRECT | DOWN | NA |
| ENSG00000173334 | TRIB1 | hsa-miR-508-5p | *H. sapiens* | HS5 | Bone Marrow | Normal/Primary | HITS-CLIP | POSITIVE | DIRECT | DOWN | NA |

**Notes**: PDHS: Pi-wei damp-heat syndrome

^a^ The PDHS-specific miRNAs and genes matched in the miRNA-gene interactions collected in the released Tarbase v7.0 (7).

**Table S11** The experimental evidences supporting the PQDS-specific miRNA-gene interaction pairs

| **geneId** | **geneName ^a^** | **mirna ^a^** | **species** | **cell_line** | **tissue** | **category** | **method** | **positive_negative** | **direct_indirect** | **up_down** | **condition** |
| --- | --- | --- | --- | --- | --- | --- | --- | --- | --- | --- | --- |
| ENSG00000122786 | *CALD1* | hsa-miR-122-5p | *H. sapiens* | HEPG2 | Liver | Cancer/Malignant | Biotin-Microarrays | POSITIVE | INDIRECT | DOWN | NA |
| ENSG00000122786 | *CALD1* | hsa-miR-122-5p | *H. sapiens* | NA | NA | NA | pSILAC | POSITIVE | INDIRECT | NA | NA |
| ENSG00000134871 | *COL4A2* | hsa-miR-122-5p | *H. sapiens* | HEPG2 | Liver | Cancer/Malignant | Biotin-Microarrays | POSITIVE | INDIRECT | DOWN | NA |
| ENSG00000184916 | *JAG2* | hsa-miR-122-5p | *H. sapiens* | HEPG2 | Liver | Cancer/Malignant | Biotin-Microarrays | POSITIVE | INDIRECT | DOWN | NA |
| ENSG00000138119 | *MYOF* | hsa-miR-122-5p | *H. sapiens* | HEPG2 | Liver | Cancer/Malignant | Biotin-Microarrays | POSITIVE | INDIRECT | DOWN | NA |
| ENSG00000140479 | *PCSK6* | hsa-miR-122-5p | *H. sapiens* | HEPG2 | Liver | Cancer/Malignant | Biotin-Microarrays | POSITIVE | INDIRECT | DOWN | NA |
| ENSG00000154447 | *SH3RF1* | hsa-miR-122-5p | *H. sapiens* | HEPG2 | Liver | Cancer/Malignant | Biotin-Microarrays | POSITIVE | INDIRECT | DOWN | NA |
| ENSG00000078399 | *HOXA9* | hsa-miR-126-3p | *H. sapiens* | 293S | Kidney | NA | HITS-CLIP | POSITIVE | DIRECT | DOWN | treatment: emetine |
| ENSG00000101000 | *PROCR* | hsa-miR-126-3p | *H. sapiens* | HUVEC | Umbilical Vein | Normal/Primary | HITS-CLIP | POSITIVE | DIRECT | DOWN | NA |
| ENSG00000118785 | *SPP1* | hsa-miR-126-3p | *H. sapiens* | A375M | Skin | Cancer/Malignant | Luciferase Reporter Assay | NEGATIVE | DIRECT | NA | NA |
| ENSG00000118785 | *SPP1* | hsa-miR-126-3p | *H. sapiens* | A375M | Skin | Cancer/Malignant | Western Blot | POSITIVE | INDIRECT | DOWN | NA |
| ENSG00000118785 | *SPP1* | hsa-miR-126-3p | *H. sapiens* | ME665/1 | Skin | Cancer/Malignant | Western Blot | POSITIVE | INDIRECT | DOWN | NA |
| ENSG00000170345 | *FOS* | hsa-miR-136-5p | *H. sapiens* | HS5 | Bone Marrow | Normal/Primary | HITS-CLIP | POSITIVE | DIRECT | DOWN | NA |
| ENSG00000111961 | *SASH1* | hsa-miR-136-5p | *H. sapiens* | HS5 | Bone Marrow | Normal/Primary | HITS-CLIP | POSITIVE | DIRECT | DOWN | NA |
| ENSG00000154447 | *SH3RF1* | hsa-miR-136-5p | *H. sapiens* | HS5 | Bone Marrow | Normal/Primary | HITS-CLIP | POSITIVE | DIRECT | DOWN | NA |
| ENSG00000109321 | *AREG* | hsa-miR-205-5p | *H. sapiens* | CASKI | Cervix | Cancer/Malignant | AGO-IP | POSITIVE | INDIRECT | DOWN | miR-205 mimic transfection |
| ENSG00000128567 | *PODXL* | hsa-miR-205-5p | *H. sapiens* | CASKI | Cervix | Cancer/Malignant | AGO-IP | POSITIVE | INDIRECT | DOWN | miR-205 mimic transfection |
| ENSG00000136193 | *SCRN1* | hsa-miR-205-5p | *H. sapiens* | CASKI | Cervix | Cancer/Malignant | AGO-IP | POSITIVE | INDIRECT | DOWN | miR-205 mimic transfection |
| ENSG00000171729 | *TMEM51* | hsa-miR-205-5p | *H. sapiens* | CASKI | Cervix | Cancer/Malignant | AGO-IP | POSITIVE | INDIRECT | DOWN | miR-205 mimic transfection |
| ENSG00000115641 | *FHL2* | hsa-miR-299-5p | *H. sapiens* | HS27A | Bone Marrow | Normal/Primary | HITS-CLIP | POSITIVE | DIRECT | DOWN | NA |
| ENSG00000115641 | *FHL2* | hsa-miR-449a | *H. sapiens* | BETA Cells | Pancreas | Normal/Primary | HITS-CLIP | POSITIVE | DIRECT | DOWN | NA |
| ENSG00000184916 | *JAG2* | hsa-miR-449a | *H. sapiens* | 293S | Kidney | NA | HITS-CLIP | POSITIVE | DIRECT | DOWN | treatment:emetine |
| ENSG00000148773 | *MKI67* | hsa-miR-449a | *H. sapiens* | 293S | Kidney | NA | HITS-CLIP | POSITIVE | DIRECT | DOWN | no treatment (control) |
| ENSG00000140682 | *TGFB1I1* | hsa-miR-449a | *H. sapiens* | HS5 | Bone Marrow | Normal/Primary | HITS-CLIP | POSITIVE | DIRECT | DOWN | NA |
| ENSG00000148773 | *MKI67* | hsa-miR-548d-5p | *H. sapiens* | HELA | Cervix | Cancer/Malignant | HITS-CLIP | POSITIVE | DIRECT | DOWN | Hela cells were treated with control shRNA |
| ENSG00000152078 | *TMEM56* | hsa-miR-548d-5p | *H. sapiens* | EF3DAGO2 | NA | Normal/Primary | PAR-CLIP | POSITIVE | DIRECT | DOWN | NA |
| ENSG00000160963 | *COL26A1* | hsa-miR-551b-3p | *H. sapiens* | 293S | Kidney | NA | HITS-CLIP | POSITIVE | DIRECT | DOWN | treatment:emetine |
| ENSG00000138119 | *MYOF* | hsa-miR-877-5p | *H. sapiens* | HMSC | Bone Marrow | Stem/Progenitor | HITS-CLIP | POSITIVE | DIRECT | DOWN | NA |
| ENSG00000147862 | *NFIB* | hsa-miR-877-5p | *H. sapiens* | 293S | Kidney | NA | HITS-CLIP | POSITIVE | DIRECT | DOWN | treatment: emetine |
| ENSG00000147862 | *NFIB* | hsa-miR-877-5p | *H. sapiens* | 293S | Kidney | NA | HITS-CLIP | POSITIVE | DIRECT | DOWN | treatment:hippuristanol |

**Notes**: PQDS: Pi-qi deficiency syndrome

^a^ The PQDS-specific miRNAs and genes matched with the miRNA-gene interactions collected in the released Tarbase v7.0 (7).

**Table S12** The experimentally-supported direct targets of the exosome-contained has-miR-122-5p

| **geneId** | **geneName ^a^** | **mirna ^a^** | **species** | **cell_line** | **tissue** | **category** | **method** | **positive_negative** | **direct_indirect** | **up_down** |
| --- | --- | --- | --- | --- | --- | --- | --- | --- | --- | --- |
| ENSG00000143322 | *ABL2* | hsa-miR-122-5p | *H. sapiens* | BETA Cells | Pancreas | Normal/Primary | HITS-CLIP | POSITIVE | DIRECT | DOWN |
| ENSG00000172493 | *AFF1* | hsa-miR-122-5p | *H. sapiens* | EF3DAGO2 | NA | Normal/Primary | PAR-CLIP | POSITIVE | DIRECT | DOWN |
| ENSG00000174574 | *AKIRIN1* | hsa-miR-122-5p | *H. sapiens* | BETA Cells | Pancreas | Normal/Primary | HITS-CLIP | POSITIVE | DIRECT | DOWN |
| ENSG00000149925 | *ALDOA* | hsa-miR-122-5p | *H. sapiens* | BETA Cells | Pancreas | Normal/Primary | HITS-CLIP | POSITIVE | DIRECT | DOWN |
| ENSG00000198925 | *ATG9A* | hsa-miR-122-5p | *H. sapiens* | BETA Cells | Pancreas | Normal/Primary | HITS-CLIP | POSITIVE | DIRECT | DOWN |
| ENSG00000116459 | *ATP5F1* | hsa-miR-122-5p | *H. sapiens* | BETA Cells | Pancreas | Normal/Primary | HITS-CLIP | POSITIVE | DIRECT | DOWN |
| ENSG00000114439 | *BBX* | hsa-miR-122-5p | *H. sapiens* | BC3 | NA | Cancer/Malignant | PAR-CLIP | POSITIVE | DIRECT | DOWN |
| ENSG00000173068 | *BNC2* | hsa-miR-122-5p | *H. sapiens* | BC3 | NA | Cancer/Malignant | PAR-CLIP | POSITIVE | DIRECT | DOWN |
| ENSG00000118961 | *C2orf43* | hsa-miR-122-5p | *H. sapiens* | BC3 | NA | Cancer/Malignant | PAR-CLIP | POSITIVE | DIRECT | DOWN |
| ENSG00000126247 | *CAPNS1* | hsa-miR-122-5p | *H. sapiens* | BETA Cells | Pancreas | Normal/Primary | HITS-CLIP | POSITIVE | DIRECT | DOWN |
| ENSG00000135387 | *CAPRIN1* | hsa-miR-122-5p | *H. sapiens* | BC3 | NA | Cancer/Malignant | PAR-CLIP | POSITIVE | DIRECT | DOWN |
| ENSG00000113328 | *CCNG1* | hsa-miR-122-5p | *H. sapiens* | HEP3B | Liver | Cancer/Malignant | Luciferase Reporter Assay | POSITIVE | DIRECT | DOWN |
| ENSG00000135446 | *CDK4* | hsa-miR-122-5p | *H. sapiens* | HEPG2 | Liver | Cancer/Malignant | Luciferase Reporter Assay | POSITIVE | DIRECT | DOWN |
| ENSG00000111276 | *CDKN1B* | hsa-miR-122-5p | *H. sapiens* | BC3 | NA | Cancer/Malignant | PAR-CLIP | POSITIVE | DIRECT | DOWN |
| ENSG00000169504 | *CLIC4* | hsa-miR-122-5p | *H. sapiens* | BETA Cells | Pancreas | Normal/Primary | HITS-CLIP | POSITIVE | DIRECT | DOWN |
| ENSG00000153551 | *CMTM7* | hsa-miR-122-5p | *H. sapiens* | BC3 | NA | Cancer/Malignant | PAR-CLIP | POSITIVE | DIRECT | DOWN |
| ENSG00000036257 | *CUL3* | hsa-miR-122-5p | *H. sapiens* | EF3DAGO2 | NA | Normal/Primary | PAR-CLIP | POSITIVE | DIRECT | DOWN |
| ENSG00000013563 | *DNASE1L1* | hsa-miR-122-5p | *H. sapiens* | BC3 | NA | Cancer/Malignant | PAR-CLIP | POSITIVE | DIRECT | DOWN |
| ENSG00000156171 | *DRAM2* | hsa-miR-122-5p | *H. sapiens* | EF3DAGO2 | NA | Normal/Primary | PAR-CLIP | POSITIVE | DIRECT | DOWN |
| ENSG00000205250 | *E2F4* | hsa-miR-122-5p | *H. sapiens* | EF3DAGO2 | NA | Normal/Primary | PAR-CLIP | POSITIVE | DIRECT | DOWN |
| ENSG00000107581 | *EIF3A* | hsa-miR-122-5p | *H. sapiens* | BETA Cells | Pancreas | Normal/Primary | HITS-CLIP | POSITIVE | DIRECT | DOWN |
| ENSG00000084623 | *EIF3I* | hsa-miR-122-5p | *H. sapiens* | BETA Cells | Pancreas | Normal/Primary | HITS-CLIP | POSITIVE | DIRECT | DOWN |
| ENSG00000062598 | *ELMO2* | hsa-miR-122-5p | *H. sapiens* | BETA Cells | Pancreas | Normal/Primary | HITS-CLIP | POSITIVE | DIRECT | DOWN |
| ENSG00000197930 | *ERO1L* | hsa-miR-122-5p | *H. sapiens* | EF3DAGO2 | NA | Normal/Primary | PAR-CLIP | POSITIVE | DIRECT | DOWN |
| ENSG00000164142 | *FAM160A1* | hsa-miR-122-5p | *H. sapiens* | BETA Cells | Pancreas | Normal/Primary | HITS-CLIP | POSITIVE | DIRECT | DOWN |
| ENSG00000125740 | *FOSB* | hsa-miR-122-5p | *H. sapiens* | BETA Cells | Pancreas | Normal/Primary | HITS-CLIP | POSITIVE | DIRECT | DOWN |
| ENSG00000128573 | *FOXP2* | hsa-miR-122-5p | *H. sapiens* | BC3 | NA | Cancer/Malignant | PAR-CLIP | POSITIVE | DIRECT | DOWN |
| ENSG00000104290 | *FZD3* | hsa-miR-122-5p | *H. sapiens* | EF3DAGO2 | NA | Normal/Primary | PAR-CLIP | POSITIVE | DIRECT | DOWN |
| ENSG00000115339 | *GALNT3* | hsa-miR-122-5p | *H. sapiens* | BETA Cells | Pancreas | Normal/Primary | HITS-CLIP | POSITIVE | DIRECT | DOWN |
| ENSG00000165219 | *GAPVD1* | hsa-miR-122-5p | *H. sapiens* | BETA Cells | Pancreas | Normal/Primary | HITS-CLIP | POSITIVE | DIRECT | DOWN |
| ENSG00000145990 | *GFOD1* | hsa-miR-122-5p | *H. sapiens* | EF3DAGO2 | NA | Normal/Primary | PAR-CLIP | POSITIVE | DIRECT | DOWN |
| ENSG00000108262 | *GIT1* | hsa-miR-122-5p | *H. sapiens* | BETA Cells | Pancreas | Normal/Primary | HITS-CLIP | POSITIVE | DIRECT | DOWN |
| ENSG00000169347 | *GP2* | hsa-miR-122-5p | *H. sapiens* | BETA Cells | Pancreas | Normal/Primary | HITS-CLIP | POSITIVE | DIRECT | DOWN |
| ENSG00000140443 | *IGF1R* | hsa-miR-122-5p | *H. sapiens* | Breast Cancer Cell Lines | Mammary Gland | Cancer/Malignant | Luciferase Reporter Assay | POSITIVE | DIRECT | DOWN |
| ENSG00000168264 | *IRF2BP2* | hsa-miR-122-5p | *H. sapiens* | BETA Cells | Pancreas | Normal/Primary | HITS-CLIP | POSITIVE | DIRECT | DOWN |
| ENSG00000182149 | *IST1* | hsa-miR-122-5p | *H. sapiens* | EF3DAGO2 | NA | Normal/Primary | PAR-CLIP | POSITIVE | DIRECT | DOWN |
| ENSG00000171988 | *JMJD1C* | hsa-miR-122-5p | *H. sapiens* | BC3 | NA | Cancer/Malignant | PAR-CLIP | POSITIVE | DIRECT | DOWN |
| ENSG00000143603 | *KCNN3* | hsa-miR-122-5p | *H. sapiens* | EF3DAGO2 | NA | Normal/Primary | PAR-CLIP | POSITIVE | DIRECT | DOWN |
| ENSG00000130518 | *KIAA1683* | hsa-miR-122-5p | *H. sapiens* | BETA Cells | Pancreas | Normal/Primary | HITS-CLIP | POSITIVE | DIRECT | DOWN |
| ENSG00000176619 | *LMNB2* | hsa-miR-122-5p | *H. sapiens* | BC3 | NA | Cancer/Malignant | PAR-CLIP | POSITIVE | DIRECT | DOWN |
| ENSG00000123066 | *MED13L* | hsa-miR-122-5p | *H. sapiens* | BC3 | NA | Cancer/Malignant | PAR-CLIP | POSITIVE | DIRECT | DOWN |
| ENSG00000116604 | *MEF2D* | hsa-miR-122-5p | *H. sapiens* | EF3DAGO2 | NA | Normal/Primary | PAR-CLIP | POSITIVE | DIRECT | DOWN |
| ENSG00000147649 | *MTDH* | hsa-miR-122-5p | *H. sapiens* | BETA Cells | Pancreas | Normal/Primary | HITS-CLIP | POSITIVE | DIRECT | DOWN |
| ENSG00000169894 | *MUC3A* | hsa-miR-122-5p | *H. sapiens* | BETA Cells | Pancreas | Normal/Primary | HITS-CLIP | POSITIVE | DIRECT | DOWN |
| ENSG00000184956 | *MUC6* | hsa-miR-122-5p | *H. sapiens* | BETA Cells | Pancreas | Normal/Primary | HITS-CLIP | POSITIVE | DIRECT | DOWN |
| ENSG00000173848 | *NET1* | hsa-miR-122-5p | *H. sapiens* | BC3 | NA | Cancer/Malignant | PAR-CLIP | POSITIVE | DIRECT | DOWN |
| ENSG00000197183 | *NOL4L* | hsa-miR-122-5p | *H. sapiens* | BC3 | NA | Cancer/Malignant | PAR-CLIP | POSITIVE | DIRECT | DOWN |
| ENSG00000137497 | *NUMA1* | hsa-miR-122-5p | *H. sapiens* | BC3 | NA | Cancer/Malignant | PAR-CLIP | POSITIVE | DIRECT | DOWN |
| ENSG00000125450 | *NUP85* | hsa-miR-122-5p | *H. sapiens* | BC3 | NA | Cancer/Malignant | PAR-CLIP | POSITIVE | DIRECT | DOWN |
| ENSG00000119900 | *OGFRL1* | hsa-miR-122-5p | *H. sapiens* | BC3 | NA | Cancer/Malignant | PAR-CLIP | POSITIVE | DIRECT | DOWN |
| ENSG00000101104 | *PABPC1L* | hsa-miR-122-5p | *H. sapiens* | BETA Cells | Pancreas | Normal/Primary | HITS-CLIP | POSITIVE | DIRECT | DOWN |
| ENSG00000041880 | *PARP3* | hsa-miR-122-5p | *H. sapiens* | BETA Cells | Pancreas | Normal/Primary | HITS-CLIP | POSITIVE | DIRECT | DOWN |
| ENSG00000197329 | *PELI1* | hsa-miR-122-5p | *H. sapiens* | EF3DAGO2 | NA | Normal/Primary | PAR-CLIP | POSITIVE | DIRECT | DOWN |
| ENSG00000025293 | *PHF20* | hsa-miR-122-5p | *H. sapiens* | BETA Cells | Pancreas | Normal/Primary | HITS-CLIP | POSITIVE | DIRECT | DOWN |
| ENSG00000143393 | *PI4KB* | hsa-miR-122-5p | *H. sapiens* | BC3 | NA | Cancer/Malignant | PAR-CLIP | POSITIVE | DIRECT | DOWN |
| ENSG00000105851 | *PIK3CG* | hsa-miR-122-5p | *H. sapiens* | Breast Cancer Cell Lines | Mammary Gland | Cancer/Malignant | Luciferase Reporter Assay | POSITIVE | DIRECT | DOWN |
| ENSG00000065243 | *PKN2* | hsa-miR-122-5p | *H. sapiens* | BETA Cells | Pancreas | Normal/Primary | HITS-CLIP | POSITIVE | DIRECT | DOWN |
| ENSG00000143850 | *PLEKHA6* | hsa-miR-122-5p | *H. sapiens* | BETA Cells | Pancreas | Normal/Primary | HITS-CLIP | POSITIVE | DIRECT | DOWN |
| ENSG00000198805 | *PNP* | hsa-miR-122-5p | *H. sapiens* | EF3DAGO2 | NA | Normal/Primary | PAR-CLIP | POSITIVE | DIRECT | DOWN |
| ENSG00000180228 | *PRKRA* | hsa-miR-122-5p | *H. sapiens* | HEPG2 | Liver | Cancer/Malignant | Luciferase Reporter Assay | POSITIVE | DIRECT | DOWN |
| ENSG00000132300 | *PTCD3* | hsa-miR-122-5p | *H. sapiens* | BETA Cells | Pancreas | Normal/Primary | HITS-CLIP | POSITIVE | DIRECT | DOWN |
| ENSG00000146676 | *PURB* | hsa-miR-122-5p | *H. sapiens* | BETA Cells | Pancreas | Normal/Primary | HITS-CLIP | POSITIVE | DIRECT | DOWN |
| ENSG00000112531 | *QKI* | hsa-miR-122-5p | *H. sapiens* | BETA Cells | Pancreas | Normal/Primary | HITS-CLIP | POSITIVE | DIRECT | DOWN |
| ENSG00000070950 | *RAD18* | hsa-miR-122-5p | *H. sapiens* | BC3 | NA | Cancer/Malignant | PAR-CLIP | POSITIVE | DIRECT | DOWN |
| ENSG00000144468 | *RHBDD1* | hsa-miR-122-5p | *H. sapiens* | EF3DAGO2 | NA | Normal/Primary | PAR-CLIP | POSITIVE | DIRECT | DOWN |
| ENSG00000171490 | *RSL1D1* | hsa-miR-122-5p | *H. sapiens* | BETA Cells | Pancreas | Normal/Primary | HITS-CLIP | POSITIVE | DIRECT | DOWN |
| ENSG00000010803 | *SCMH1* | hsa-miR-122-5p | *H. sapiens* | BC3 | NA | Cancer/Malignant | PAR-CLIP | POSITIVE | DIRECT | DOWN |
| ENSG00000093183 | *SEC22C* | hsa-miR-122-5p | *H. sapiens* | BC3 | NA | Cancer/Malignant | PAR-CLIP | POSITIVE | DIRECT | DOWN |
| ENSG00000105281 | *SLC1A5* | hsa-miR-122-5p | *H. sapiens* | BC3 | NA | Cancer/Malignant | PAR-CLIP | POSITIVE | DIRECT | DOWN |
| ENSG00000164209 | *SLC25A46* | hsa-miR-122-5p | *H. sapiens* | BC3 | NA | Cancer/Malignant | PAR-CLIP | POSITIVE | DIRECT | DOWN |
| ENSG00000100036 | *SLC35E4* | hsa-miR-122-5p | *H. sapiens* | BC3 | NA | Cancer/Malignant | PAR-CLIP | POSITIVE | DIRECT | DOWN |
| ENSG00000175387 | *SMAD2* | hsa-miR-122-5p | *H. sapiens* | EF3DAGO2 | NA | Normal/Primary | PAR-CLIP | POSITIVE | DIRECT | DOWN |
| ENSG00000077312 | *SNRPA* | hsa-miR-122-5p | *H. sapiens* | BC3 | NA | Cancer/Malignant | PAR-CLIP | POSITIVE | DIRECT | DOWN |
| ENSG00000126091 | *ST3GAL3* | hsa-miR-122-5p | *H. sapiens* | BC3 | NA | Cancer/Malignant | PAR-CLIP | POSITIVE | DIRECT | DOWN |
| ENSG00000055070 | *SZRD1* | hsa-miR-122-5p | *H. sapiens* | BETA Cells | Pancreas | Normal/Primary | HITS-CLIP | POSITIVE | DIRECT | DOWN |
| ENSG00000141002 | *TCF25* | hsa-miR-122-5p | *H. sapiens* | BETA Cells | Pancreas | Normal/Primary | HITS-CLIP | POSITIVE | DIRECT | DOWN |
| ENSG00000135966 | *TGFBRAP1* | hsa-miR-122-5p | *H. sapiens* | EF3DAGO2 | NA | Normal/Primary | PAR-CLIP | POSITIVE | DIRECT | DOWN |
| ENSG00000057704 | *TMCC3* | hsa-miR-122-5p | *H. sapiens* | BETA Cells | Pancreas | Normal/Primary | HITS-CLIP | POSITIVE | DIRECT | DOWN |
| ENSG00000170348 | *TMED10* | hsa-miR-122-5p | *H. sapiens* | EF3DAGO2 | NA | Normal/Primary | PAR-CLIP | POSITIVE | DIRECT | DOWN |
| ENSG00000181284 | *TMEM102* | hsa-miR-122-5p | *H. sapiens* | EF3DAGO2 | NA | Normal/Primary | PAR-CLIP | POSITIVE | DIRECT | DOWN |
| ENSG00000170006 | *TMEM154* | hsa-miR-122-5p | *H. sapiens* | BC3 | NA | Cancer/Malignant | PAR-CLIP | POSITIVE | DIRECT | DOWN |
| ENSG00000124208 | *TMEM189-UBE2V1* | hsa-miR-122-5p | *H. sapiens* | BC3 | NA | Cancer/Malignant | PAR-CLIP | POSITIVE | DIRECT | DOWN |
| ENSG00000149582 | *TMEM25* | hsa-miR-122-5p | *H. sapiens* | BC3 | NA | Cancer/Malignant | PAR-CLIP | POSITIVE | DIRECT | DOWN |
| ENSG00000103460 | *TOX3* | hsa-miR-122-5p | *H. sapiens* | BC3 | NA | Cancer/Malignant | PAR-CLIP | POSITIVE | DIRECT | DOWN |
| ENSG00000171103 | *TRMT61B* | hsa-miR-122-5p | *H. sapiens* | BC3 | NA | Cancer/Malignant | PAR-CLIP | POSITIVE | DIRECT | DOWN |
| ENSG00000104447 | *TRPS1* | hsa-miR-122-5p | *H. sapiens* | BC3 | NA | Cancer/Malignant | PAR-CLIP | POSITIVE | DIRECT | DOWN |
| ENSG00000033178 | *UBA6* | hsa-miR-122-5p | *H. sapiens* | EF3DAGO2 | NA | Normal/Primary | PAR-CLIP | POSITIVE | DIRECT | DOWN |
| ENSG00000127481 | *UBR4* | hsa-miR-122-5p | *H. sapiens* | BETA Cells | Pancreas | Normal/Primary | HITS-CLIP | POSITIVE | DIRECT | DOWN |
| ENSG00000130477 | *UNC13A* | hsa-miR-122-5p | *H. sapiens* | BETA Cells | Pancreas | Normal/Primary | HITS-CLIP | POSITIVE | DIRECT | DOWN |
| ENSG00000162607 | *USP1* | hsa-miR-122-5p | *H. sapiens* | BC3 | NA | Cancer/Malignant | PAR-CLIP | POSITIVE | DIRECT | DOWN |
| ENSG00000152484 | *USP12* | hsa-miR-122-5p | *H. sapiens* | BETA Cells | Pancreas | Normal/Primary | HITS-CLIP | POSITIVE | DIRECT | DOWN |
| ENSG00000106346 | *USP42* | hsa-miR-122-5p | *H. sapiens* | BC3 | NA | Cancer/Malignant | PAR-CLIP | POSITIVE | DIRECT | DOWN |
| ENSG00000160131 | *VMA21* | hsa-miR-122-5p | *H. sapiens* | BC3 | NA | Cancer/Malignant | PAR-CLIP | POSITIVE | DIRECT | DOWN |
| ENSG00000062716 | *VMP1* | hsa-miR-122-5p | *H. sapiens* | BC3 | NA | Cancer/Malignant | PAR-CLIP | POSITIVE | DIRECT | DOWN |
| ENSG00000062716 | *VMP1* | hsa-miR-122-5p | *H. sapiens* | EF3DAGO2 | NA | Normal/Primary | PAR-CLIP | POSITIVE | DIRECT | DOWN |
| ENSG00000048707 | *VPS13D* | hsa-miR-122-5p | *H. sapiens* | BETA Cells | Pancreas | Normal/Primary | HITS-CLIP | POSITIVE | DIRECT | DOWN |
| ENSG00000133316 | *WDR74* | hsa-miR-122-5p | *H. sapiens* | BC3 | NA | Cancer/Malignant | PAR-CLIP | POSITIVE | DIRECT | DOWN |
| ENSG00000137207 | *YIPF3* | hsa-miR-122-5p | *H. sapiens* | BC3 | NA | Cancer/Malignant | PAR-CLIP | POSITIVE | DIRECT | DOWN |
| ENSG00000177888 | *ZBTB41* | hsa-miR-122-5p | *H. sapiens* | EF3DAGO2 | NA | Normal/Primary | PAR-CLIP | POSITIVE | DIRECT | DOWN |
| ENSG00000169554 | *ZEB2* | hsa-miR-122-5p | *H. sapiens* | BETA Cells | Pancreas | Normal/Primary | HITS-CLIP | POSITIVE | DIRECT | DOWN |
| ENSG00000106261 | *ZKSCAN1* | hsa-miR-122-5p | *H. sapiens* | BC3 | NA | Cancer/Malignant | PAR-CLIP | POSITIVE | DIRECT | DOWN |
| ENSG00000085644 | *ZNF213* | hsa-miR-122-5p | *H. sapiens* | EF3DAGO2 | NA | Normal/Primary | PAR-CLIP | POSITIVE | DIRECT | DOWN |

**Notes**:

^a^ The validated miRNA-gene interactions collected in the released Tarbase v7.0 (7).

**Supplemental Figures**


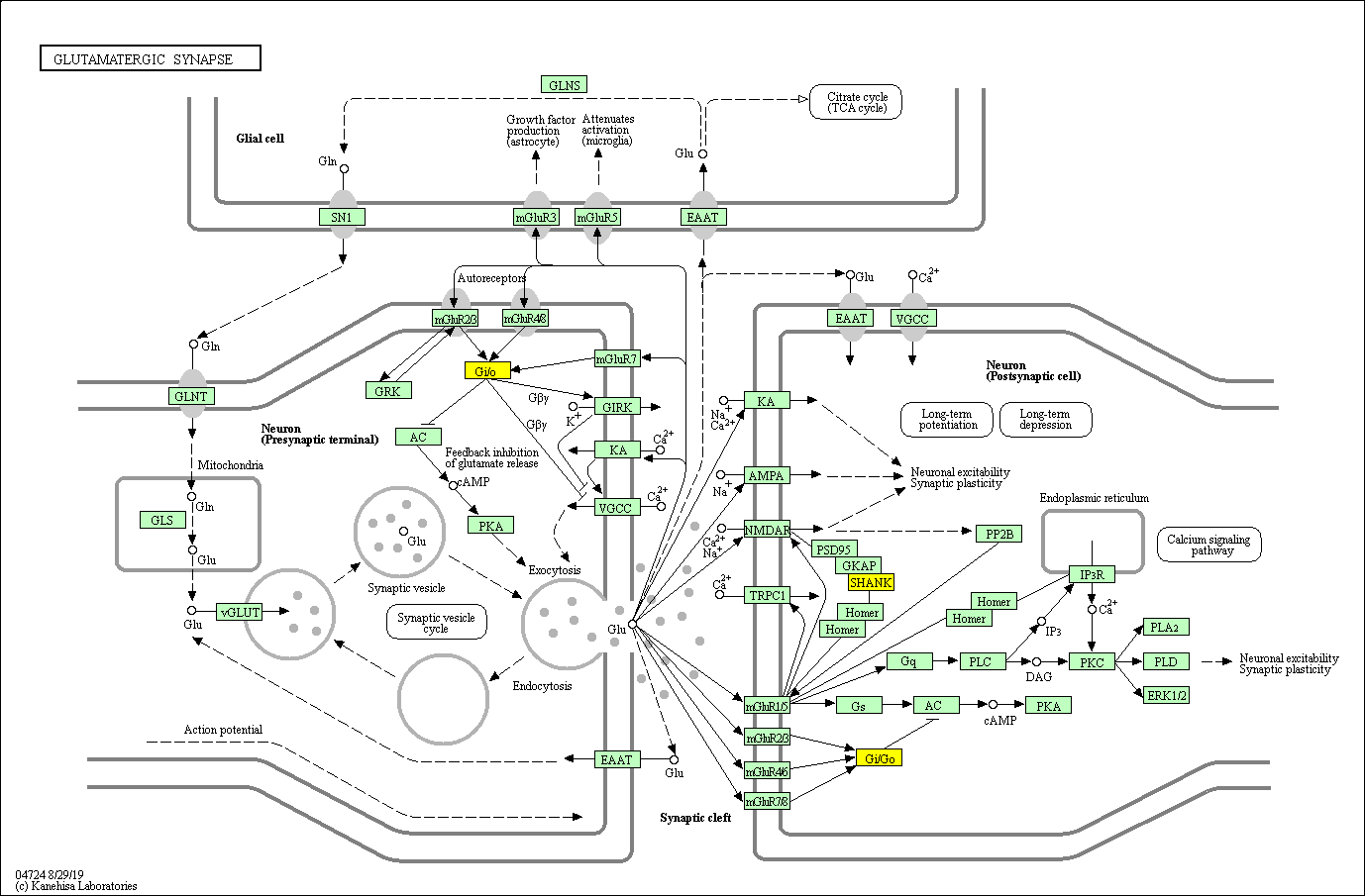


**Figure S1** Glutamatergic synapse pathway (hsa04724).

The PDHS-specific genes (yellow) in this pathway containing the SHANK1, GNAI1, GNG10, GNAO1. For the detailed overview of the pathway, it is referred to the web address below.

<https://www.genome.jp/kegg-bin/show_pathway?hsa04724/hsa:50944%20yellow/hsa:2770%20yellow/hsa:2790%20yellow/hsa:2775%20yellow>

***Description***: Glutamate is the major excitatory neurotransmitter in the mammalian central nervous system(CNS). Glutamate is packaged into synaptic vesicles in the presynaptic terminal. Once released into the synaptic cleft, glutamate acts on postsynaptic ionotropic glutamate receptors (iGluRs) to mediate fast excitatory synaptic transmission. Glutamate can also act on metabotropic glutamate receptors (mGluRs) and exert a variety of modulatory effects through their coupling to G proteins and the subsequent recruitment of second messenger systems. Presynaptically localized Group II and Group III mGluRs are thought to represent the classical inhibitory autoreceptor mechanism that suppresses excess glutamate release. After its action on these receptors, glutamate can be removed from the synaptic cleft by EAATs located either on the presynaptic terminal, neighboring glial cells, or the postsynaptic neuron. In glia, glutamate is converted to glutamine, which is then transported back to the presynaptic terminal and converted back to glutamate.


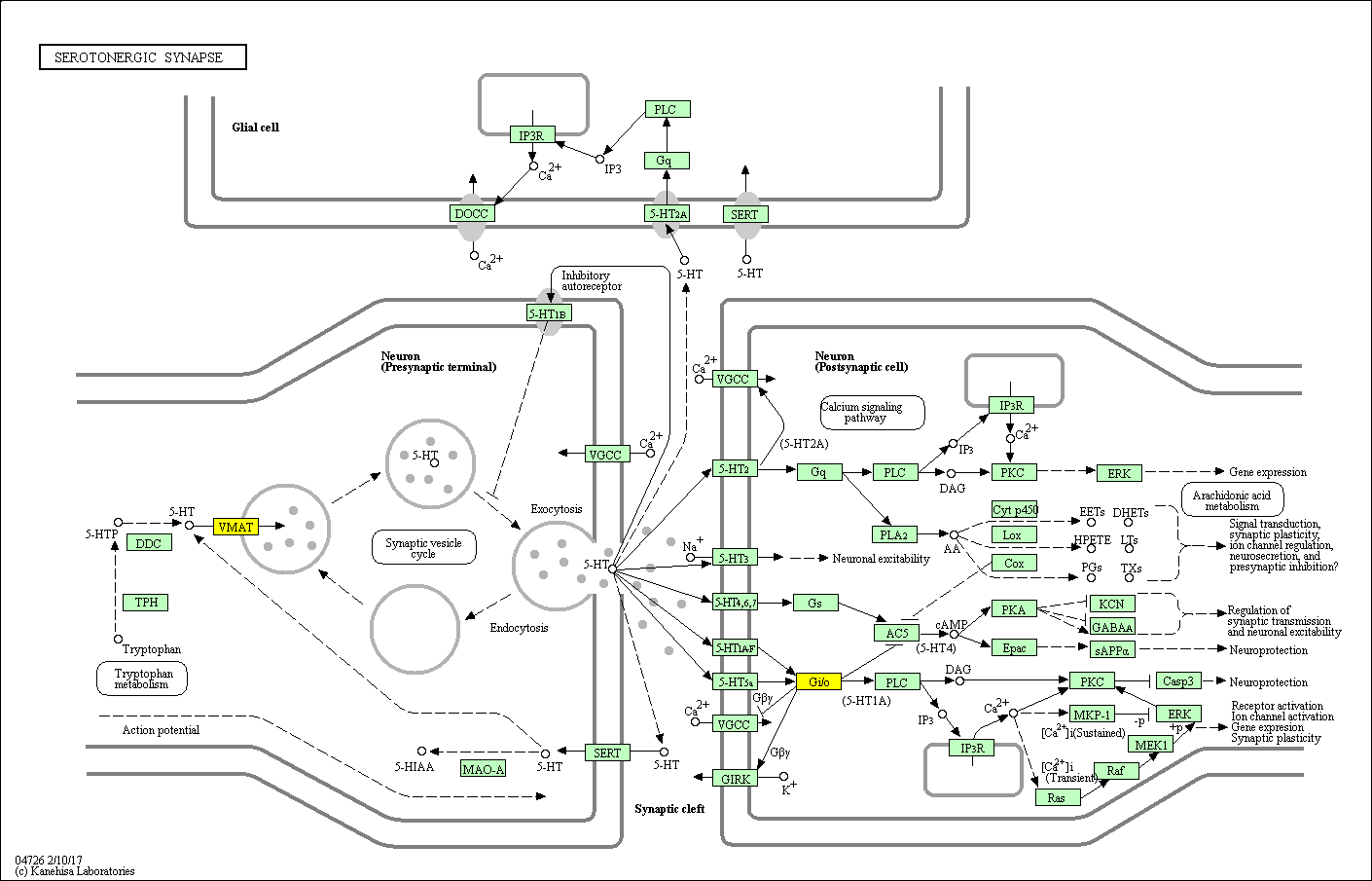


**Figure S2** Serotonergic/Dopaminergic synapse pathway (hsa04726).

The PDHS-specific genes (yellow) in this pathway containing the SLC18A1, GNAI1, GNG10, GNAO1. For the detailed overview of the pathway, it is referred to the web address below.

<https://www.genome.jp/kegg-bin/show_pathway?hsa04726/hsa:6570%20yellow/hsa:2770%20yellow/hsa:2790%20yellow/hsa:2775%20yellow>

***Description***: Serotonin (5-Hydroxytryptamine, 5-HT) is a monoamine neurotransmitter that plays important roles in physiological functions such as learning and memory, emotion, sleep, pain, motor function and endocrine secretion, as well as in pathological states including abnormal mood and cognition. Once released from presynaptic axonal terminals, 5-HT binds to receptors, which have been divided into 7 subfamilies on the basis of conserved structures and signaling mechanisms. These families include the ionotropic 5-HT3 receptors and G-protein-coupled 5-HT receptors, the 5-HT1 (Gi /Go -coupled), 5-HT2(Gq-coupled), 5-HT4/6/7 (Gs-coupled) and 5-HT5 receptors. Presynaptically localized 5-HT1B receptors are thought to be the autoreceptors that suppress excess 5-HT release. 5-HT's actions are terminated by transporter- mediated reuptake into neurons, leading to catabolism by monoamine oxidase.


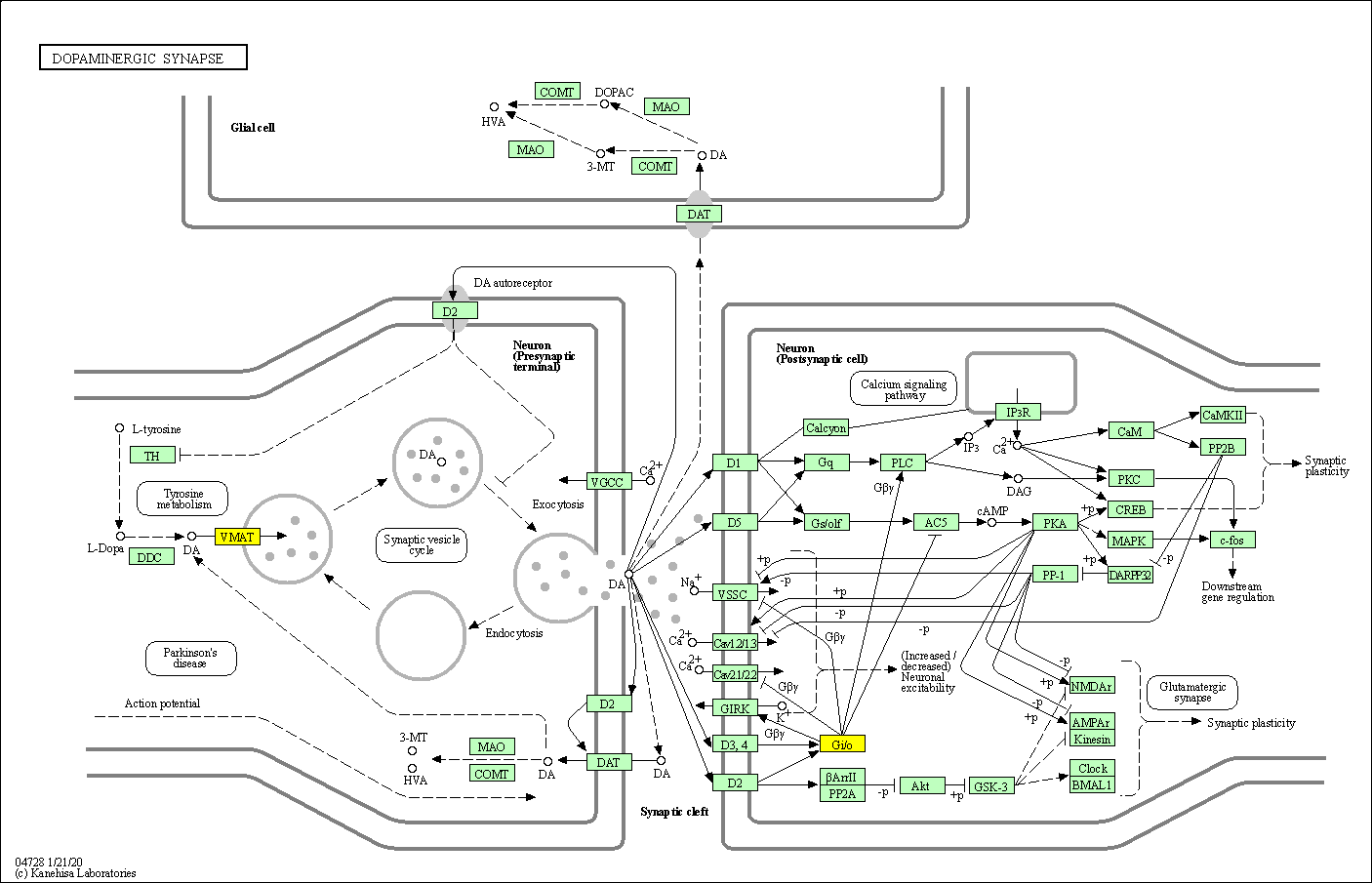


**Figure S3** Dopaminergic synapse pathway (hsa04728).

The PDHS-specific genes (yellow) in this pathway containing the SLC18A1, GNAI1, GNG10, GNAO1. For the detailed overview of the pathway, it is referred to the web address below.

<https://www.genome.jp/kegg-bin/show_pathway?hsa04728/hsa:6570%20yellow/hsa:2770%20yellow/hsa:2790%20yellow/hsa:2775%20yellow>

***Description***: Dopamine (DA) is an important and prototypical slow neurotransmitter in the mammalian brain, where it controls a variety of functions including locomotor activity, motivation and reward, learning and memory, and endocrine regulation. Once released from presynaptic axonal terminals, DA interacts with at least five receptor subtypes in the central nervous system (CNS), which have been divided into two groups: the D1-like receptors (D1Rs), comprising D1 and D5 receptors, both positively coupled to adenylyl cyclase and cAMP production, and the D2-like receptors (D2Rs), comprising D2, D3, and D4 receptors, whose activation results in inhibition of adenylyl cyclase and suppression of cAMP production. In addition, D1Rs and D2Rs modulate intracellular Ca2+ levels and a number of Ca2+ -dependent intracellular signaling processes. Through diverse cAMP- and Ca2+-dependent and - independent mechanisms, DA influences neuronal activity, synaptic plasticity, and behavior. Presynaptically localized D2Rs regulate synthesis and release of DA as the main autoreceptor of the dopaminergic system.


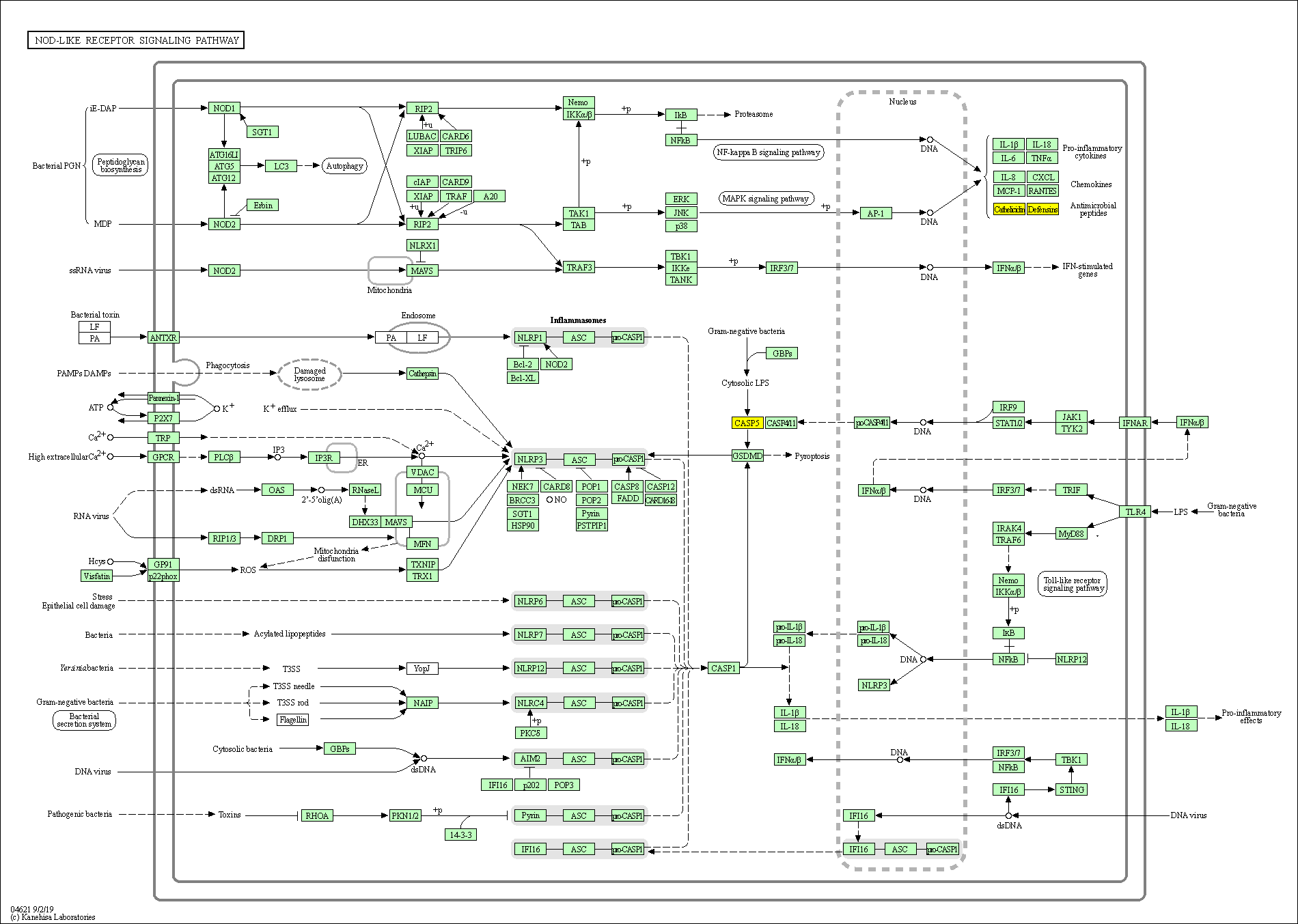


**Figure S4** NOD-like receptor signaling pathway (hsa04621).

The PDHS-specific genes (yellow) in this pathway containing the DEFA3, CASP5, DEFA1B, CAMP, DEFA1. For the detailed overview of the pathway, it is referred to the web address below.

<https://www.genome.jp/kegg-bin/show_pathway?hsa04621/hsa:1668%20yellow/hsa:838%20yellow/hsa:728358%20yellow/hsa:820%20yellow/hsa:1667%20yellow>

***Description***: Specific families of pattern recognition receptors are responsible for detecting various pathogens and generating innate immune responses. The intracellular NOD-like receptor (NLR) family contains more than 20 members in mammals and plays a pivotal role in the recognition of intracellular ligands. NOD1 and NOD2, two prototypic NLRs, sense the cytosolic presence of the bacterial peptidoglycan fragments that escaped from endosomal compartments, driving the activation of NF-{kappa}B and MAPK, cytokine production and apoptosis. On the other hand, a different set of NLRs induces caspase-1 activation through the assembly of multiprotein complexes called inflammasomes. The activated of caspase-1 regulates maturation of the pro-inflammatory cytokines IL-1B, IL-18 and drives pyroptosis.


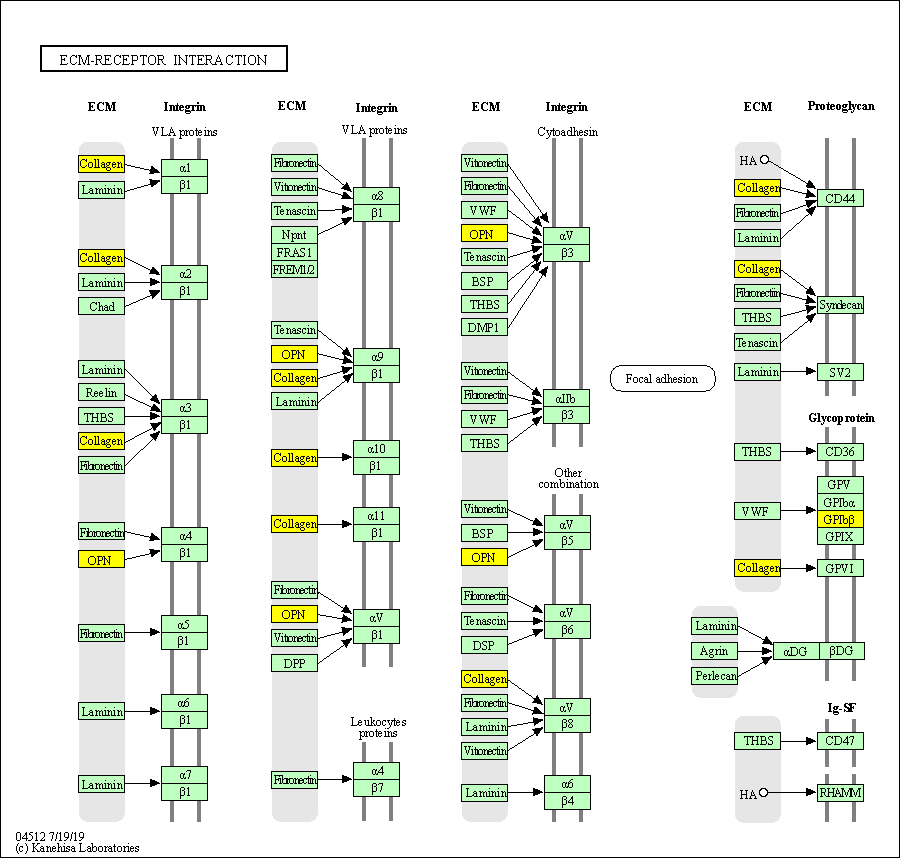


**Figure S5** ECM-receptor interaction (hsa04512).

The PQDS-specific genes (yellow) in this pathway containing the COL4A2, SPP1, GP1BB, COL4A4. For the detailed overview of the pathway, it is referred to the web address below.

<https://www.genome.jp/kegg-bin/show_pathway?hsa04512/hsa:1284%20yellow/hsa:6696%20yellow/hsa:2812%20yellow/hsa:1286%20yellow>.

***Description***: The extracellular matrix (ECM) consists of a complex mixture of structural and functional macromolecules and serves an important role in tissue and organ morphogenesis and in the maintenance of cell and tissue structure and function. Specific interactions between cells and the ECM are mediated by transmembrane molecules, mainly integrins and perhaps also proteoglycans, CD36, or other cell-surface-associated components. These interactions lead to a direct or indirect control of cellular activities such as adhesion, migration, differentiation, proliferation, and apoptosis. In addition, integrins function as mechanoreceptors and provide a force-transmitting physical link between the ECM and the cytoskeleton. Integrins are a family of glycosylated, heterodimeric transmembrane adhesion receptors that consist of noncovalently bound alpha- and beta-subunits.


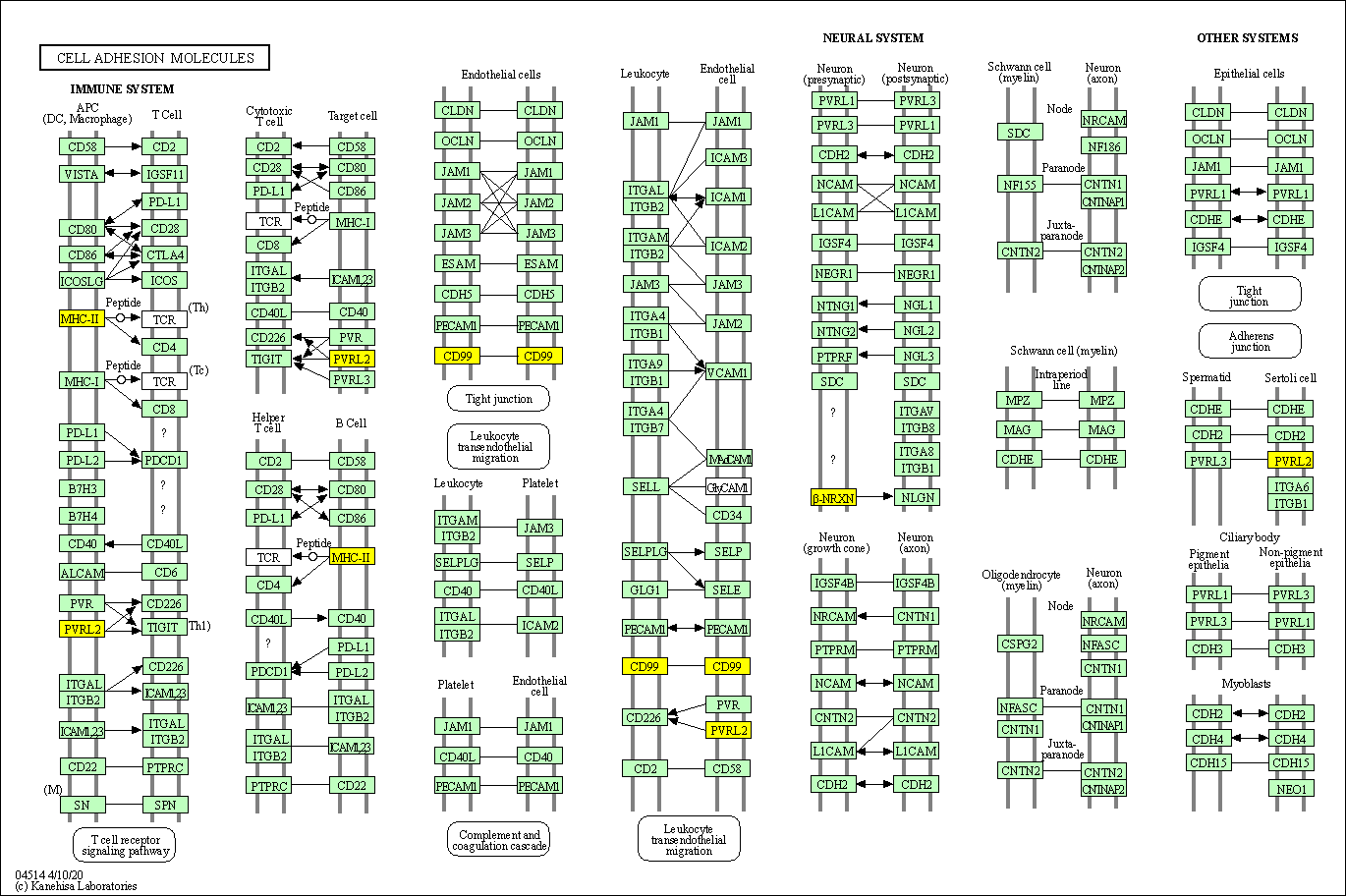


**Figure S6** Cell adhesion molecules (CAMs) (hsa04514).

The PQDS-specific genes (yellow) in this pathway containing the HLA-DRB5, NECTIN2, NRXN1, CD99, HLA-DQB1. For the detailed overview of the pathway, it is referred to the web address below.

<https://www.genome.jp/kegg-bin/show_pathway?hsa04514/hsa:3127%20yellow/hsa:5819%20yellow/hsa:9378%20yellow/hsa:4267%20yellow/hsa:3119%20yellow>.

***Description***: Cell adhesion molecules are (glyco)proteins expressed on the cell surface and play a critical role in a wide array of biologic processes that include hemostasis, the immune response, inflammation, embryogenesis, and development of neuronal tissue. There are four main groups: the integrin family, the immunoglobulin superfamily, selectins, and cadherins. Membrane proteins that mediate immune cell–cell interactions fall into different categories, namely those involved in antigen recognition, costimulation and cellular adhesion. Furthermore cell-cell adhesions are important for brain morphology and highly coordinated brain functions such as memory and learning. During early development of the nervous system, neurons elongate their axons towards their targets and establish and maintain synapses through formation of cell-cell adhesions. Cell-cell adhesions also underpin axon-axon contacts and link neurons with supporting schwann cells.


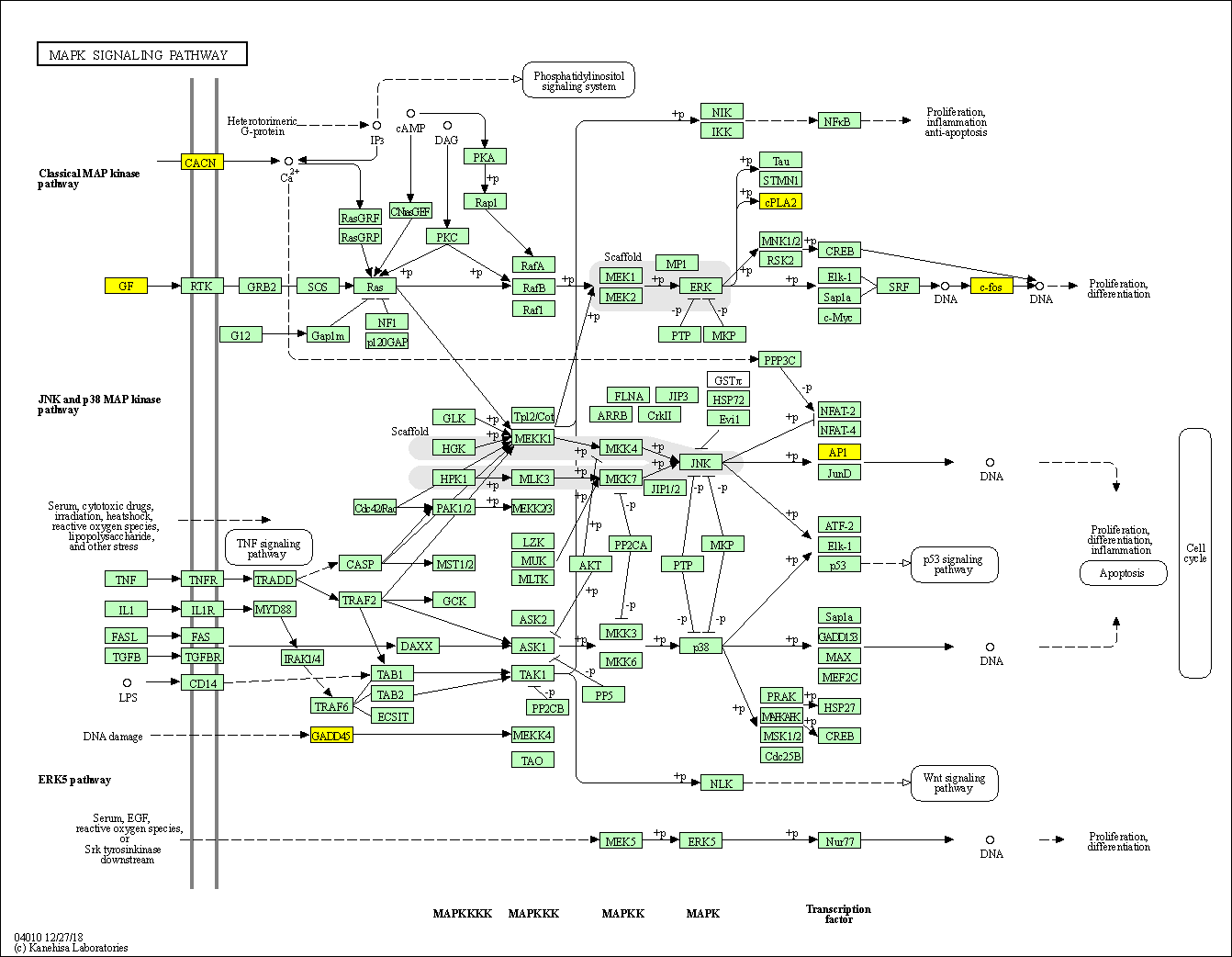


**Figure S7** MAPK signaling pathway (hsa04010).

The PQDS-specific genes (yellow) in this pathway containing the EGF, GADD45G, PLA2G4C, FOS, PLA2G4B, AREG, CACNB2. For the detailed overview of the pathway, it is referred to the web address below.

<https://www.genome.jp/kegg-bin/show_pathway?hsa04010/hsa:1950%20yellow/hsa:10912%20yellow/hsa:8605%20yellow/hsa:2353%20yellow/hsa:100137049%20yellow/hsa:374%20yellow/hsa:783%20yellow>

***Description***: The mitogen-activated protein kinase (MAPK) cascade is a highly conserved module that is involved in various cellular functions, including cell proliferation, differentiation and migration. Mammals express at least four distinctly regulated groups of MAPKs, extracellular signal-related kinases (ERK)-1/2, Jun amino-terminal kinases (JNK1/2/3), p38 proteins (p38alpha/beta/gamma/delta) and ERK5, that are activated by specific MAPKKs: MEK1/2 for ERK1/2, MKK3/6 for the p38, MKK4/7 (JNKK1/2) for the JNKs, and MEK5 for ERK5. Each MAPKK, however, can be activated by more than one MAPKKK, increasing the complexity and diversity of MAPK signalling. Presumably each MAPKKK confers responsiveness to distinct stimuli. For example, activation of ERK1/2 by growth factors depends on the MAPKKK c-Raf, but other MAPKKKs may activate ERK1/2 in response to pro-inflammatory stimuli.


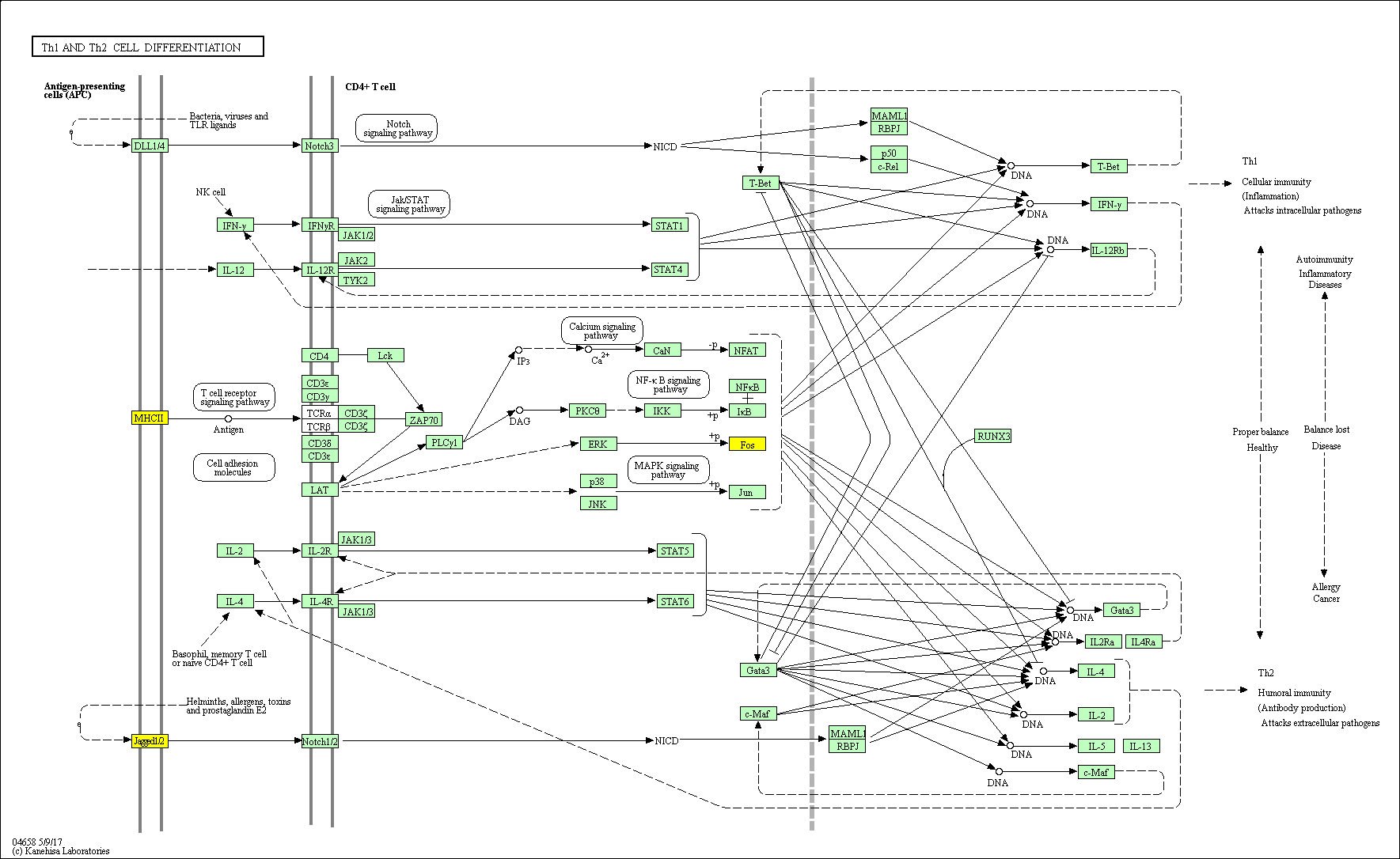


**Figure S8** Th1 and Th2 cell differentiation (hsa04658).

The PQDS-specific genes (yellow) in this pathway containing the JAG2, HLA-DRB5, FOS, HLA-DQB1. For the detailed overview of the pathway, it is referred to the web address below.

<https://www.genome.jp/kegg-bin/show_pathway?hsa04658/hsa:3714%20yellow/hsa:3127%20yellow/hsa:2353%20yellow/hsa:3119%20yellow>

***Description***: Immunity to different classes of microorganisms is orchestrated by separate lineages of effector T helper (TH)-cells, which differentiate from naive CD4+ precursor cells in response to cues provided by antigen presenting cells (APC) and include T helper type 1 (Th1) and Th2. Th1 cells are characterized by the transcription factor T-bet and signal transducer and activator of transcription (STAT) 4, and the production of IFN-gamma. These cells stimulate strong cell-mediated immune responses, particularly against intracellular pathogens. On the other hand, transcription factors like GATA-3 and STAT6 drive the generation of Th2 cells that produce IL-4, IL-5 and IL-13 and are necessary for inducing the humoral response to combat parasitic helminths (type 2 immunity) and isotype switching to IgG1 and IgE. The balance between Th1/Th2 subsets determines the susceptibility to disease states, where the improper development of Th2 cells can lead to allergy, while an overactive Th1 response can lead to autoimmunity.


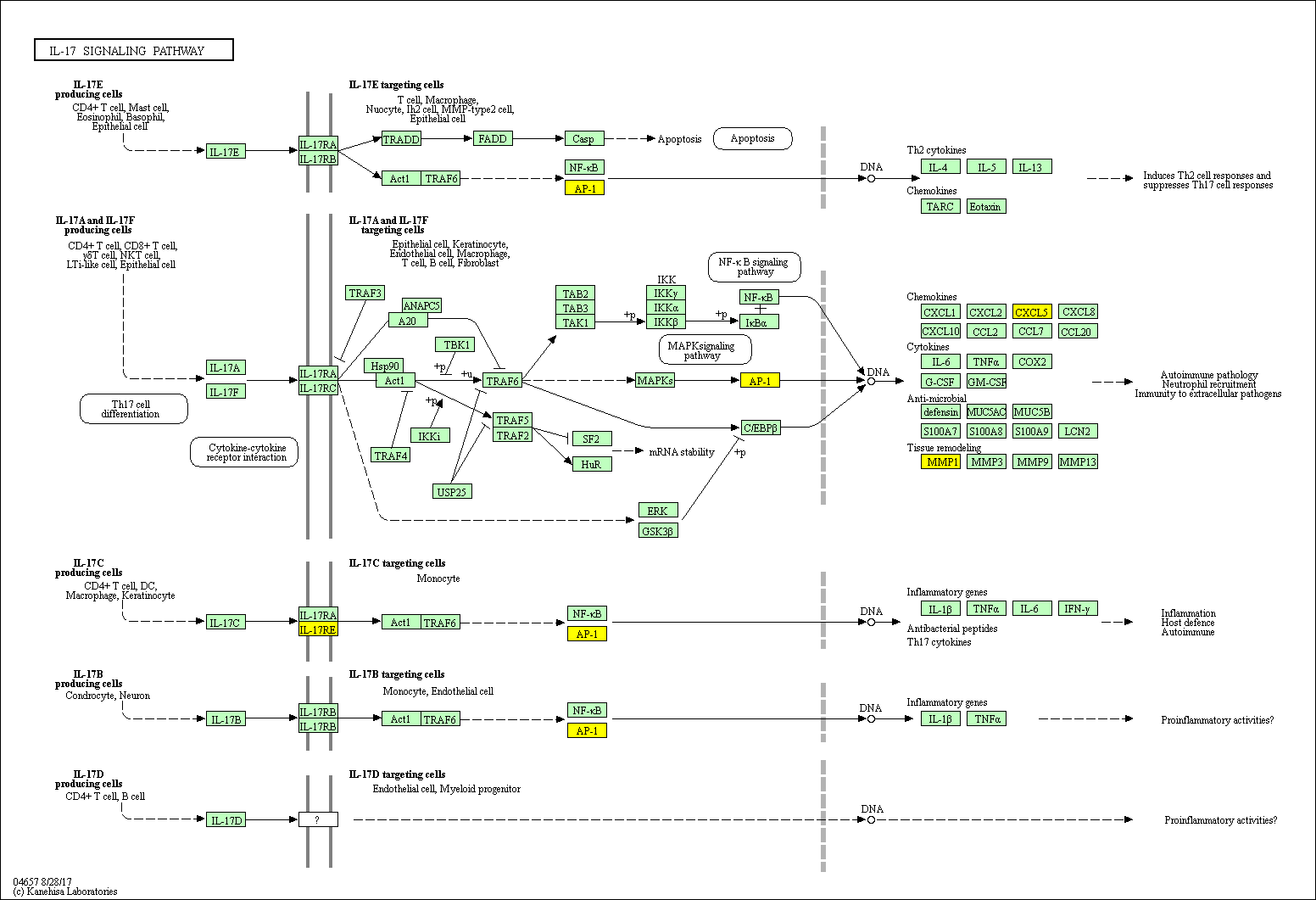


**Figure S9** IL-17 signaling pathway (hsa04657).

The PQDS-specific genes (yellow) in this pathway containing the CXCL6, FOS, MMP1, IL17RE. For the detailed overview of the pathway, it is referred to the web address below.

<https://www.genome.jp/kegg-bin/show_pathway?hsa04657/hsa:6372%20yellow/hsa:2353%20yellow/hsa:4312%20yellow/hsa:132014%20yellow>

***Description***: The interleukin 17 (IL-17) family, a subset of cytokines consisting of IL-17A-F, plays crucial roles in both acute and chronic inflammatory responses. IL-17A, the hallmark cytokine of the newly defined T helper 17 (TH17) cell subset, has important roles in protecting the host against extracellular pathogens, but also promotes inflammatory pathology in autoimmune disease, whereas IL-17F is mainly involved in mucosal host defense mechanisms. IL-17E (IL-25) is an amplifier of Th2 immune responses. IL-17C has biological functions similar to those of IL-17A. The functions of IL-17B and IL-17D remain largely elusive. The IL-17 family signals via their correspondent receptors and activates downstream pathways that include NF-kappaB, MAPKs and C/EBPs to induce the expression of antimicrobial peptides, cytokines and chemokines. The receptor proximal adaptor Act1 (an NF-kappaB activator 1) is considered as the master mediator in IL-17A signaling. It is likely that Act1 is a common signal adaptor also shared by other members mediated signalings in this family.


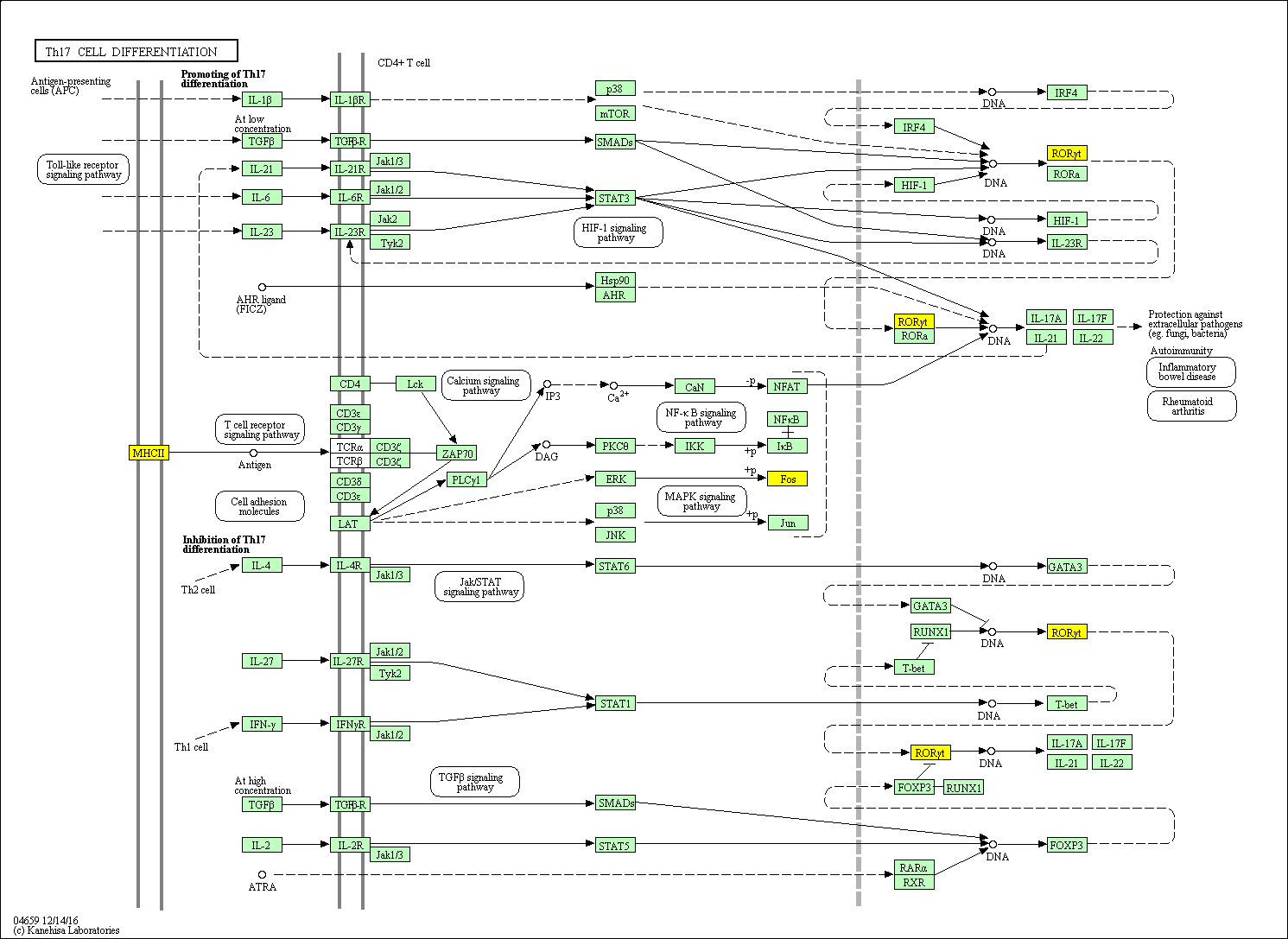


**Figure S10** Th17 cell differentiation pathway (hsa04659).

The PQDS-specific genes (yellow) in this pathway containing the HLA-DRB5, RORC, FOS, HLA-DQB1. For the detailed overview of the pathway, it is referred to the web address below.

<https://www.genome.jp/kegg-bin/show_pathway?hsa04659/hsa:3127%20yellow/hsa:6097%20yellow/hsa:2353%20yellow/hsa:3119%20yellow>

***Description***: Interleukin (IL)-17-producing helper T (Th17) cells serve as a subset of CD4+ T cells involved in epithelial cell- and neutrophil mediated immune responses against extracellular microbes and in the pathogenesis of autoimmune diseases. In vivo, Th17 differentiation requires antigen presentation and co-stimulation, and activation of antigen presenting-cells (APCs) to produce TGF-beta, IL-6, IL-1, IL-23 and IL-21. This initial activation results in the activation and up-regulation of STAT3, ROR(gamma)t and other transcriptional factors in CD4+ T cells, which bind to the promoter regions of the IL-17, IL-21 and IL-22 genes and induce IL-17, IL-21 and IL-22. In contrast, the differentiation of Th17 cells and their IL-17 expression are negatively regulated by IL-2, Th2 cytokine IL-4, IL-27 and Th1 cytokine IFN-gamma through STAT5, STAT6 and STAT1 activation, respectively. Retinoid acid and the combination of IL-2 and TGF-beta upregulate Foxp3, which also downregulates cytokines like IL-17 and IL-21. The inhibition of Th17 differentiation may serve as a protective strategy to 'fine-tune' the expression IL-17 so it does not cause excessive inflammation. Thus, balanced differentiation of Th cells is crucial for immunity and host protection.


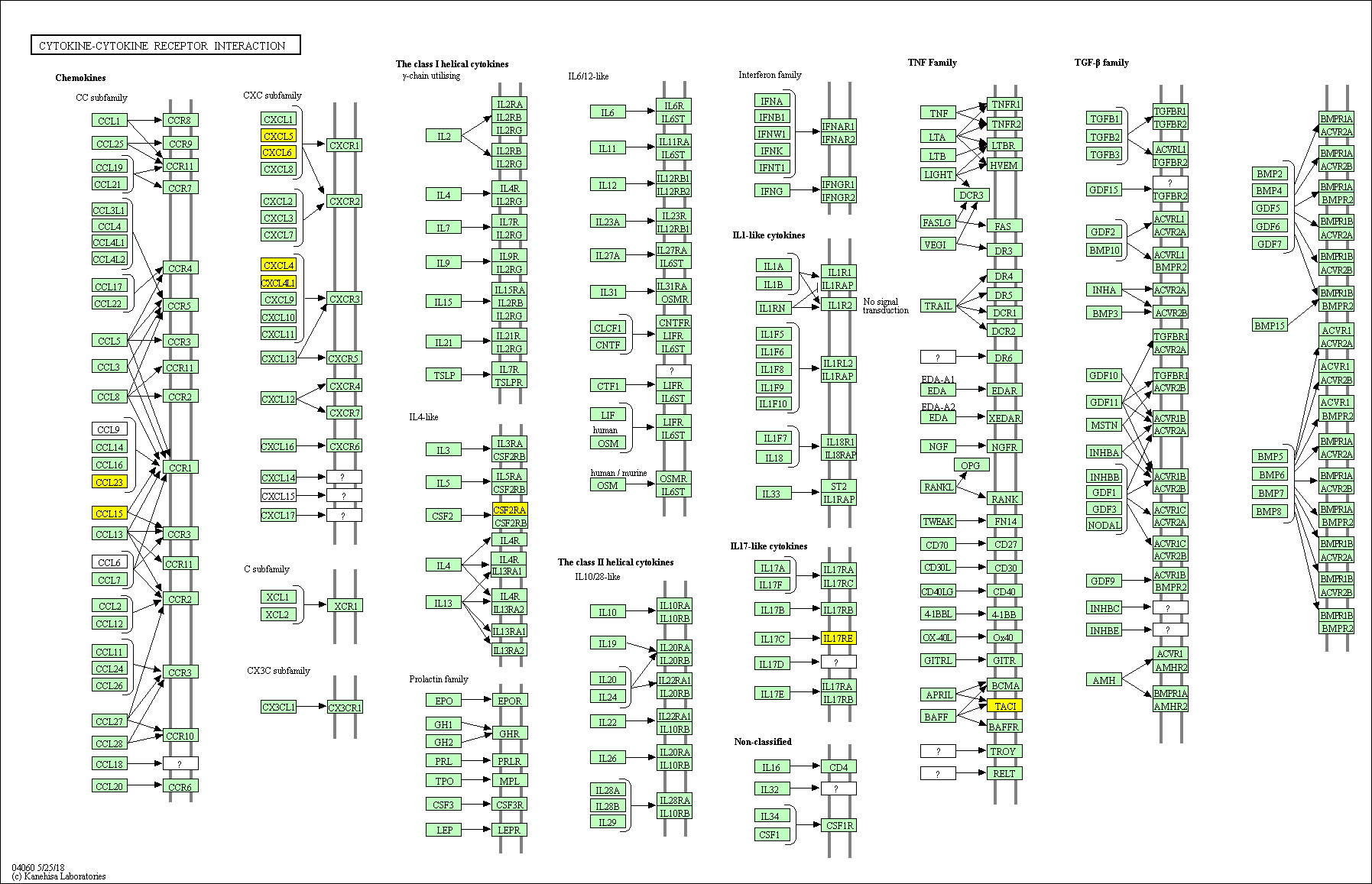


**Figure S11** Cytokine-cytokine receptor interaction pathway (hsa04060).

The PQDS-specific genes (yellow) in this pathway containing the TNFRSF13B, CCL23, CXCL6, CSF2RA, PF4, IL17RE. For the detailed overview of the pathway, it is referred to the web address below.

<https://www.genome.jp/kegg-bin/show_pathway?hsa04060/hsa:23495%20yellow/hsa:6368%20yellow/hsa:6372%20yellow/hsa:1438%20yellow/hsa:5196%20yellow/hsa:132014%20yellow>

***Description***: Cytokines are soluble extracellular proteins or glycoproteins that are crucial intercellular regulators and mobilizers of cells engaged in innate as well as adaptive inflammatory host defenses, cell growth, differentiation, cell death, angiogenesis, and development and repair processes aimed at the restoration of homeostasis. Cytokines are released by various cells in the body, usually in response to an activating stimulus, and they induce responses through binding to specific receptors on the cell surface of target cells. Cytokines can be grouped by structure into different families and their receptors can likewise be grouped.


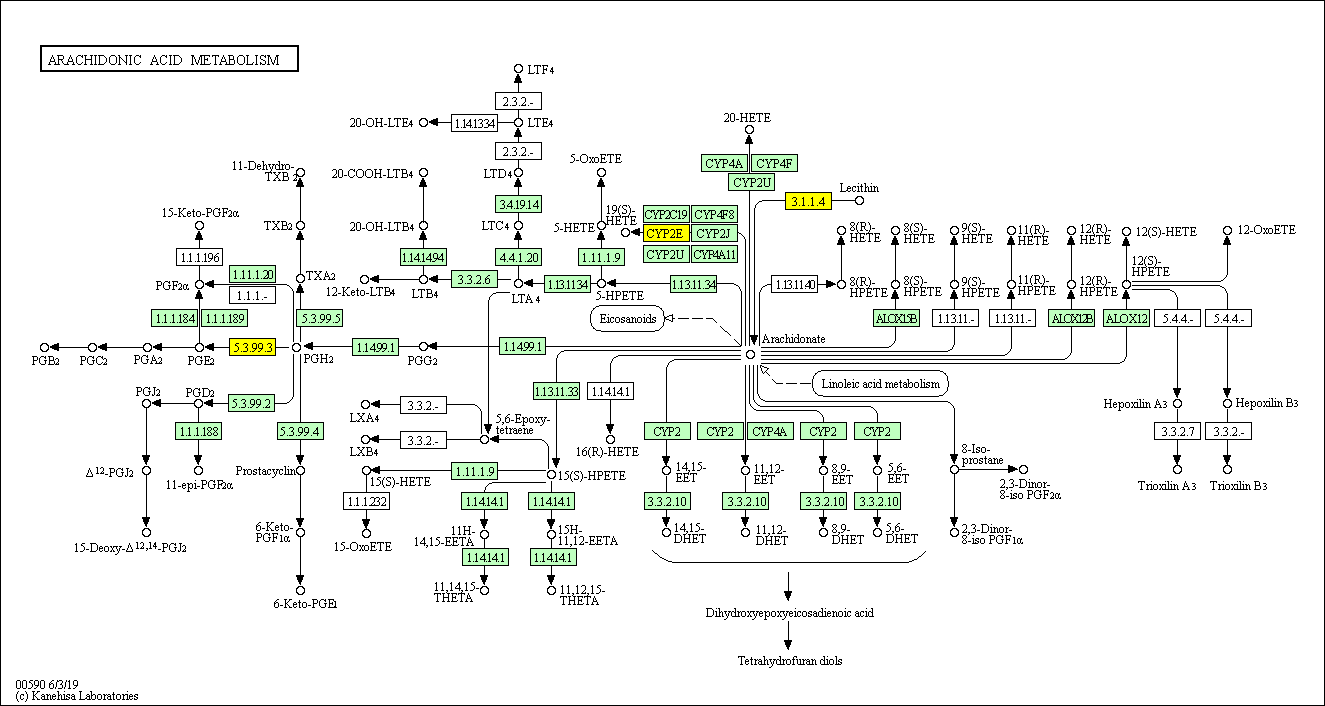


**Figure S12** Arachidonic acid metabolism (hsa590).

The PQDS-specific genes (yellow) in this pathway containing the PLA2G4C, CYP2E1, PLA2G4B, PTGES. For the detailed overview of the pathway, it is referred to the web address below.

<https://www.genome.jp/kegg-bin/show_pathway?hsa00590/hsa:8605%20yellow/hsa:1571%20yellow/hsa:100137049%20yellow/hsa:9536%20yellow>


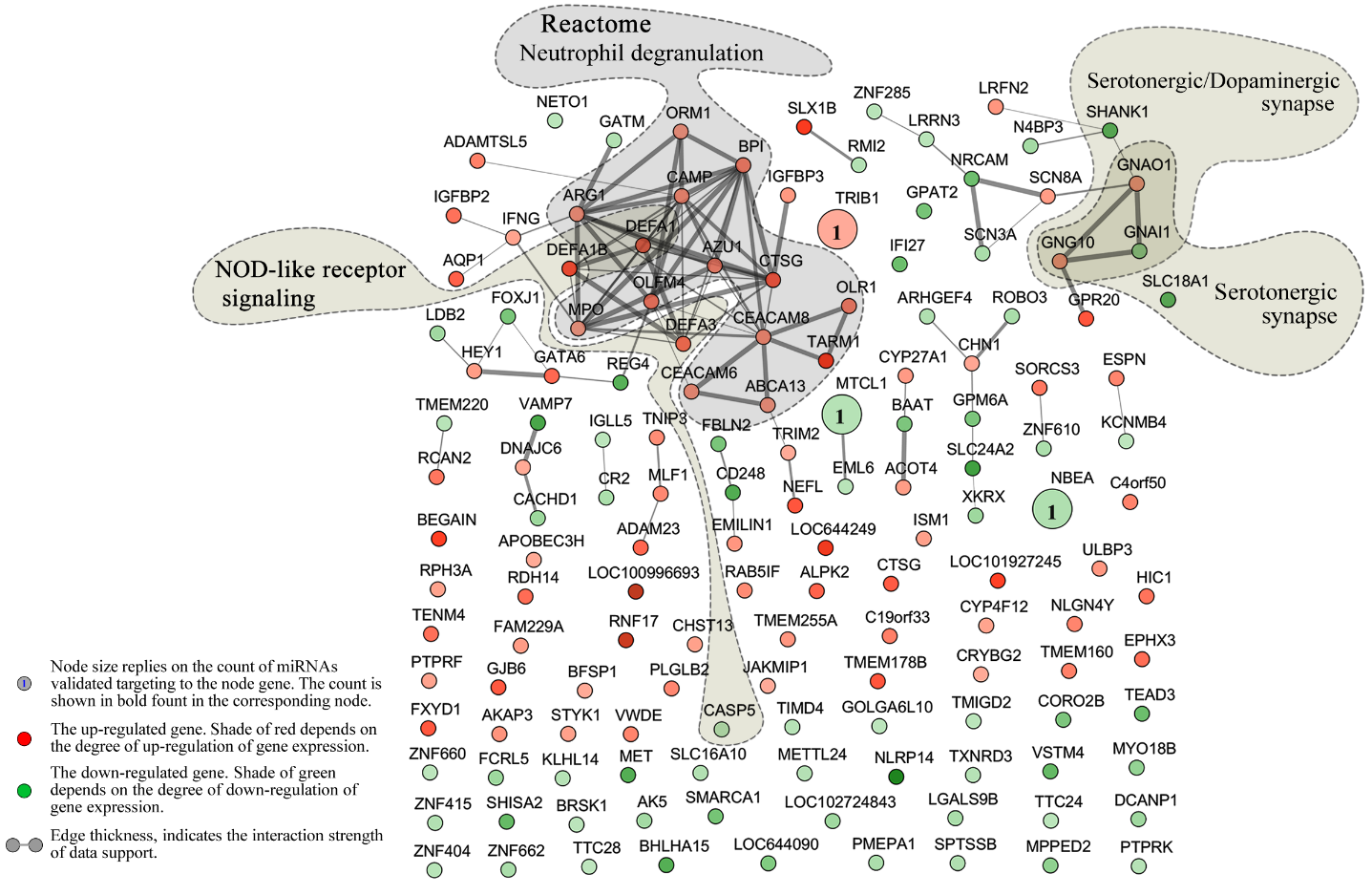


**Figure S13** The network detailing the interaction relationships of the PDHS-specific genes in leukocytes.

The blue number marked in a node indicates the count of the PDHS-specific miRNAs which were validated targeting to the corresponding node gene. The node genes-enriched pathways were specially marked in the generated interaction network. ***Abbreviations***: PDHS, chronic atrophic gastritis patients with Pi-wei damp-heat syndrome.


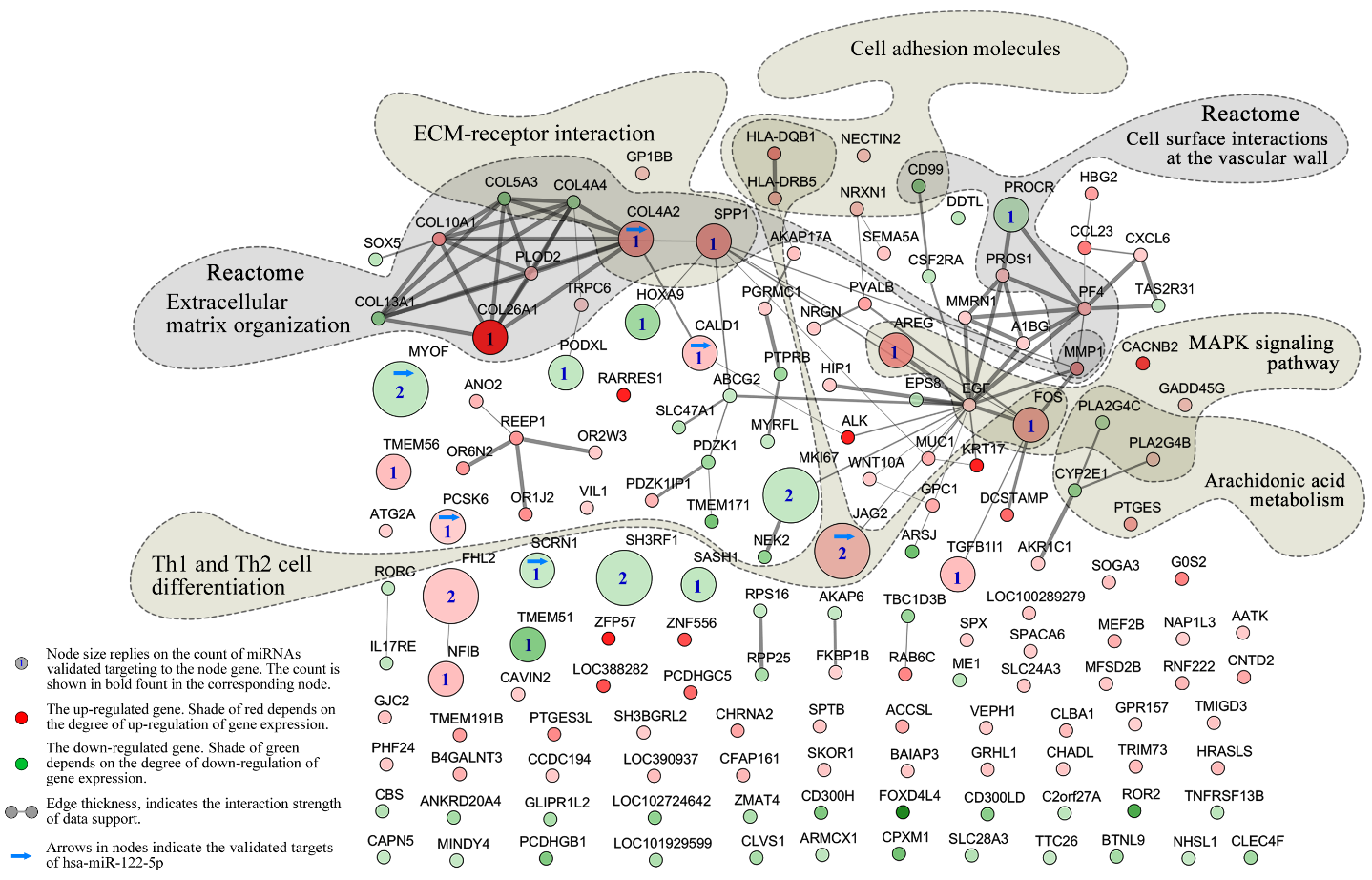


**Figure S14** The network detailing the interaction relationships of the PQDS-specific genes in leukocytes.

The blue number marked in a node indicates the count of the PQDS-specific miRNAs which were validated targeting to the corresponding node gene. The node genes-enriched pathways were specially marked in the generated interaction network. ***Abbreviations***: PQDS, chronic atrophic gastritis patients with Pi-qi-deficiency syndrome.

**References:**

1. Fang JY, Liu WZ, Li ZK, et al: China Chronic Gastritis Consensus (2012, Shanghai). Chin J Front Med Sci 5: 44-55, 2013.

2. Zheng XY: Guiding Principle for Clinical Research on New Drugs of Traditional Chinese Medicine. China Medical Science Press, Beijing, 2002.

3. Patel RK and Jain M: NGS QC Toolkit: a toolkit for quality control of next generation sequencing data. Plos One 7: e30619, 2012.

4. Altschul SF, Madden TL, Schäffer AA, et al: Gapped BLAST and PSI-BLAST: a new generation of protein database search programs. Nucleic acids research 25: 3389-3402, 1997.

5. Daub J, Eberhardt RY, Tate JG and Burge SW: Rfam: annotating families of non-coding RNA sequences. Methods in molecular biology (Clifton, N.J.) 1269: 349-363, 2015.

6. An JY, Lai J, Lehman ML and Nelson CC: miRDeep*: an integrated application tool for miRNA identification from RNA sequencing data. Nucleic acids research 41: 727-737, 2013.

7. Vlachos IS, Paraskevopoulou MD, Karagkouni D, et al: DIANA-TarBase v7.0: indexing more than half a million experimentally supported miRNA:mRNA interactions. Nucleic Acids Res. 43: D153-159, 2015.
